# Bioactive (3*Z*,5*E*)-11,20-Epoxybriara-3,5-dien-7,18-olide Diterpenoids from the South China Sea Gorgonian *Dichotella gemmacea*

**DOI:** 10.3390/md9081403

**Published:** 2011-08-16

**Authors:** Cui Li, Ming-Ping La, Peng Sun, Tibor Kurtan, Attila Mandi, Hua Tang, Bao-Shu Liu, Yang-Hua Yi, Ling Li, Wen Zhang

**Affiliations:** 1 Research Center for Marine Drugs, School of Pharmacy, Second Military Medical University, 325 Guo-He Road, Shanghai 200433, China; E-Mails: licuiwan@163.com (C.L.); lmp12@163.com (M.-P.L.); sunp918@hotmail.com (P.S.); tanghua0309@126.com (H.T.); liubaoshu@126.com (B.-S.L.); yiyanghua@hotmail.com (Y.-H.Y.); 2 Department of Organic Chemistry, University of Debrecen, POB 20, 4010 Debrecen, Hungary; E-Mails: kurtant@tigris.klte.hu (T.K.); mandia@delfin.klte.hu (A.M.)

**Keywords:** briarane diterpenoids, biological activity, structure elucidation, gorgonian, *Dichotella gemmacea*

## Abstract

Six new (3*Z*,5*E*)-11,20-epoxybriara-3,5-dien-7,18-olide diterpenoids, gemmacolides N–S (**1**–**6**), were isolated together with four known analogues, juncenolide D, and juncins R, S and U (**7**–**10**), from the South China Sea gorgonian *Dichotella gemmacea*. The structures of the new compounds were elucidated by the detailed analysis of spectroscopic data in combination with the comparison with reported data. The absolute configuration of **1** was determined by a TDDFT calculation of its solution ECD spectrum, affording the determination of absolute configuration of other analogues by simply comparing their ECD spectra with that of **1**. The cytotoxic and antimicrobial activities of these compounds were evaluated. In preliminary *in vitro* bioassays, compounds **4**, **5**, **6**, **8** and **9** showed cytotoxicity against A549 and MG63, while compounds **1**, **2**, **4**, **7**–**10** showed antimicrobial activity against the fungus *Septoria tritici* and the bacterium *Escherichia coli*.

## 1. Introduction

Briarane-type diterpenoids are a group of highly oxidized secondary metabolites reported from marine organisms, particularly from octocorals [1]. These kinds of molecules are derived from cembrane diterpenoids by 3,8-cyclization, constructing of a fused six- and 10-membered bicyclic carbon skeleton, and also display an additional γ-lactone ring fused with the 10-membered ring [2]. The first example of such kind of metabolites, briarein A, was isolated from the Caribbean octocoral *Briareum asbestinum* in 1977 [3]. Since then, a series of briarane diterpenoids have been continuously reported in an increasing number due to the structural complexity and interesting biological activities, such as cytotoxic [4–8], anti-inflammatory [9–11], antiviral [9,12,13], antifouling [14,15], insecticidal [16,17], and immunomodulatory effects [18]. Briarane diterpenoids with the chemical feature of 11,20-epoxy-3,5(6)-diene moiety have only been reported so far from two species of gorgonian of the family Ellisellidae, *i.e.*, *Junceella gemmacea* (*Dichotella gemmacea*) [19] collected from Pohnpei of Micronesia [20], and *Junceella juncea* collected from Tuticorin [21] and the South China Sea [14,22]. Some of these compounds exhibited weak cytotoxicity against human liver carcinoma (HEPA 59T/VGH) and oral epidermoid (KB) carcinoma cell lines [23] and, in addition, antifouling activities against the larval settlement of barnacle *Balanus amphitrite* [14]. However, the absolute configurations of these compounds have not been determined yet, due to the flexibility of the 10-membered ring and the complexity of the molecules.

**Chart 1.**
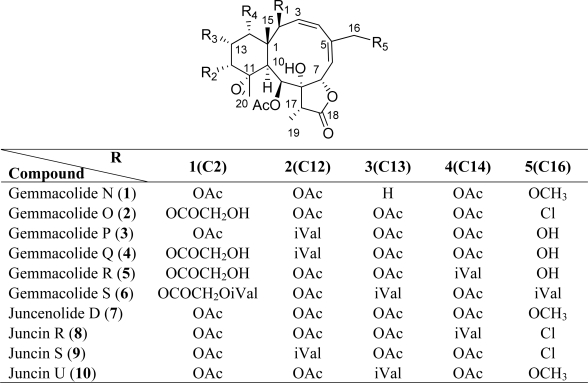
Structures of compounds **1**–**10**.

In the course of our ongoing screening for biologically active secondary metabolites from marine sources [24–27], we made a collection of the gorgonian *Dichotella gemmacea* off the coast of Beihai, China. The species of *D. gemmacea* was identified previously as *J. gemmacea* [19]. Early chemical studies on the species resulted in the isolation and characterization of 18 briarane diterpenoids, including gemmacolides A–F [20], dichotellides A–E, praelolide, juncin P, juncin ZI, junceellin A [28], and three unnamed analogues [29]. Preliminary investigation of the crude extract of *D. gemmacea* in our laboratory led to the isolation and structural determination of seven new bioactive diterpenoids, namely gemmacolides G–M [30]. Our continuous investigation on the trace compounds of the crude extract has now resulted in the isolation of additional six new members of this family, namely gemmacolides N–S (**1**–**6**), together with four known analogues, juncenolide D (**7**) [23], and juncins R, S and U (**8**–**10**) [14] (Chart 1). Structures of the new compounds were elucidated by detailed analysis of their spectroscopic data aided by the comparison with reported data of related derivatives. The absolute configuration of **1** was determined by time-dependent density functional theory electroniccircular dichroism (TDDFT ECD) calculation of the computed solution conformers. This method allowed for the configurational assignment of additional five new and four known analogues. Gemmacolide N (**1**) was chosen as an ECD reference in the determination of absolute configuration for this group of metabolites.

## 2. Results and Discussion

Freshly collected specimens of *D. gemmacea* were immediately frozen to −20 °C and stored at this temperature before extraction. The EtOAc-soluble portion of the combined extracts of acetone and methanol was subsequently partitioned between MeOH and *n*-hexane. The MeOH extract was subjected to sequential column chromatography on silicagel, Sephadex LH-20, and the RP-HPLC to afford 10 diterpenoids (**1**–**10**). The structures and relative stereochemistry of the known compounds **7**–**10** were determined by extensive spectroscopic analysis combined with careful comparison with reported data. Juncenolide D (**7**) was once reported from the Taiwanese gorgonian *Junceella juncea* with its structure being elucidated on the basis of FABMS, UV, IR and NMR techniques [23]. Structures of juncins R (**8**), S (**9**) and U (**10**), previously obtained from the South China Sea gorgonian *Junceella juncea*, were also established by extensive spectroscopic analysis [14]. The absolute configuration, however, of **7**–**10** remained unsolved.

Gemmacolide N (**1**) was isolated as a white amorphous powder (1.1 mg). Its molecular formula was established as C_29_H_38_O_13_ by HRESI-MS, indicating 11 degrees of double bond equivalence. The IR spectrum showed absorption bands of hydroxyl (3475 cm^−1^), a γ-lactone (1776 cm^−1^), and ester (1739 cm^−1^) functionalities. This observation was in agreement with the signals in the ^13^C NMR and DEPT spectra (Table 1) for nine sp^2^ carbon atoms (5× OC═O, CH═CH, CH═C) at lower field (accounting for seven double bond equivalents) and twenty sp^3^ carbon atoms at higher field (1× C, 2× CH, 1× CH_2_, 6× CH_3_, 2× OC, 5× OCH, 2× OCH_2_, 1× OCH_3_). The remaining double bond equivalents were due to the presence of four rings in the molecule.

**Figure 1.**
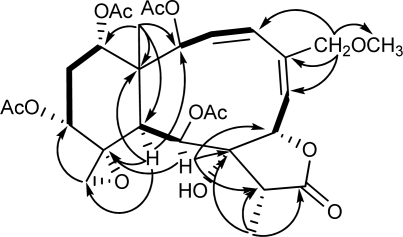
Key HMBC (arrow H→C) and COSY (bond) spin coupling systems for compound **1**.

**Figure 2.**
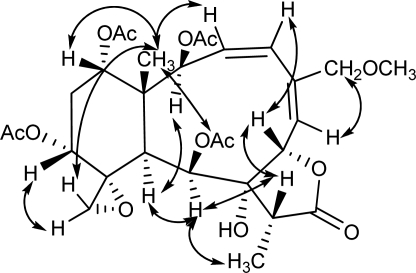
Key NOESY correlations for compound **1**.

^1^H and ^13^C NMR spectra of **1** showed great similarity to those of juncenolide D (**7**) [23], except that one of the acetoxy groups in **7** (δ_H_ 1.96, s; δ_C_ 169.6, s, 20.5, q; see Experimental) was replaced by a hydrogen in **1**. This proton was assigned as part of CH_2_-13 (δ_H_ 2.31, 1.92, each, ov., δ_C_ 28.5, t; Tables 1 and 2) due to the proton sequence of H-12/H_2_-13/H-14, established by the ^1^H-^1^H COSY experiment (Figure 1). The established planar structure of **1** was further supported by the ^1^H-^1^H COSY and HMBC spectra as shown in Figure 1. The relative configuration of **1** at the chiral centers was proved the same as that of **7** by a NOESY experiment (Figure 2), showing a β configuration of H-7, H-12, H-14, Me-15, H-17, and CH_2_-20, and a α configuration of H-2, H-9, H-10, and Me-19. The geometry of the Δ^3^ double bond was assigned as (*Z*) based on the proton coupling constant between H-3 and H-4 (*J* = 10.7 Hz) while that of Δ^5^ was determined as (*E*) due to the NOESY correlation between H-6 and H_2_-16. Assignments of NMR signals for structure of **1** were strongly supported by comparison with the reported data of frajunolide D, an analogue isolated from the gorgonian *Junceella fragilis* [31]. Frajunolide D differs from **1** only in the functional group at C-16, having an acetoxy group instead of a methoxy group. The relative stereochemistry of **1** was thus determined as (1R*,2S*,7S*,8S*,9S*,10S*,11R*,12R*,14S*,17R*).

**Figure 3.**
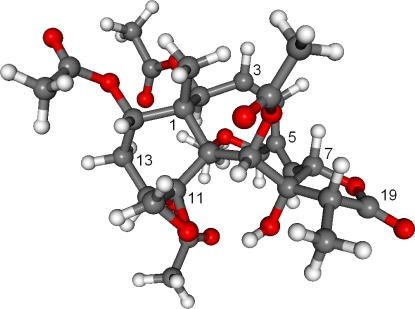
Structure of the lowest-energy conformer (43.4% population) of the (1*R*,2*S*,7*S*,8*S*,9*S*,10*S*,11*R*,12*R*,14*S*,17*R*)-enantiomer of **1**.

**Figure 4.**
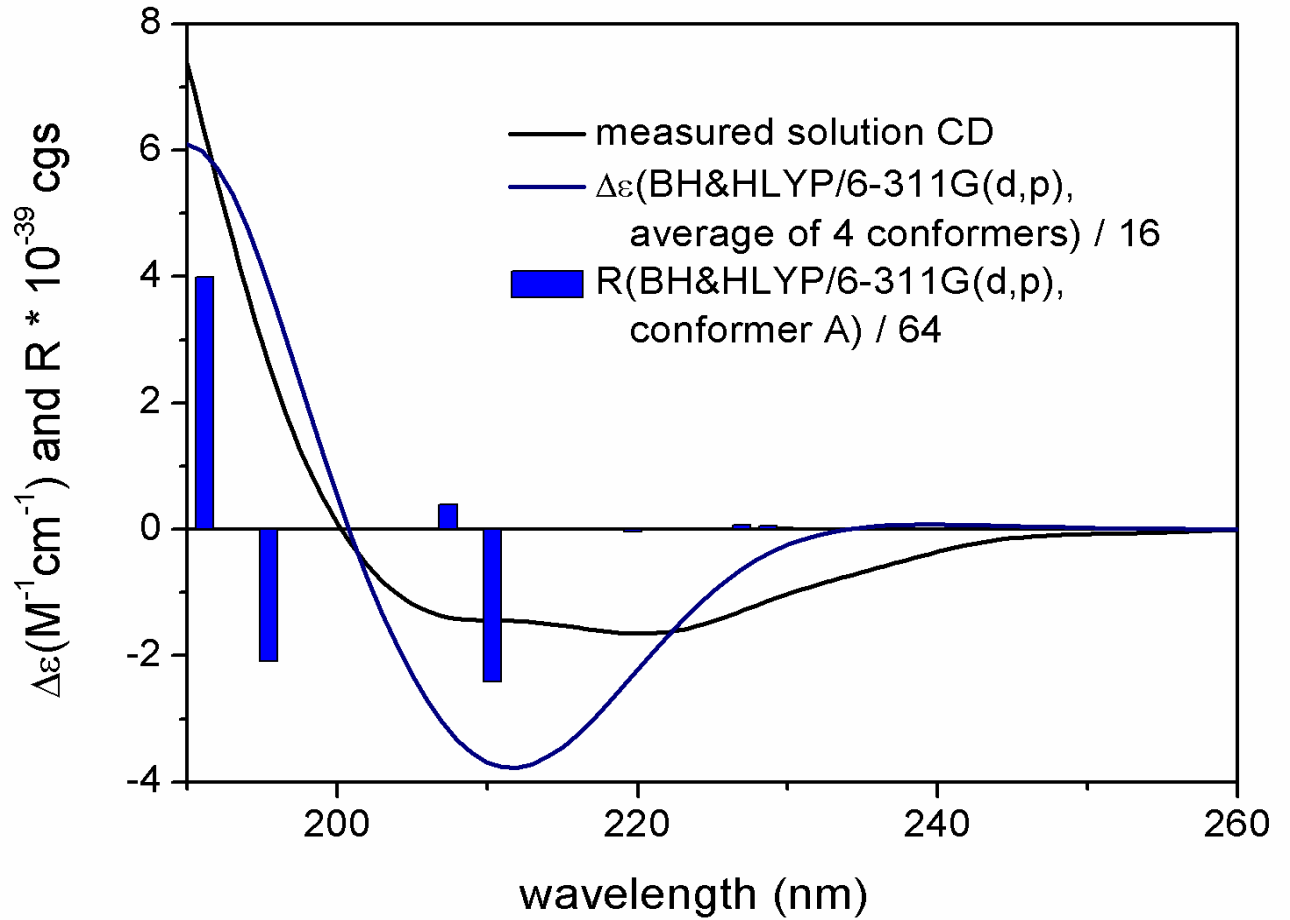
Experimental solution ECD spectrum of gemmacolide N (**1**) compared with the Boltzmann-weighted BH&HLYP/6-311G(d,p) ECD spectrum calculated for four conformational isomers of the (1*R*,2*S*,7*S*,8*S*,9*S*,10*S*,11*R*,12*R*,14*S*,17*R*)-enantiomer of **1**. Bars represent the rotational strength of the lowest-energy conformers with the BH&HLYP/6-311G(d,p) method.

The absolute configuration of **1** was determined by TDDFT ECD calculation of the solution conformers. Although gemmacolide N (**1**) has zero specific rotation in chloroform, it showed a distinct solution ECD spectrum with a negative ECD transition in the region 250–200 nm and a positive band below 200 nm, which derives from the lactone and diene chromophores. The MMFF conformational search of **1** followed by B3LYP/6-31G(d) optimization provided four slightly different conformers (43.4%, 17.4% 14.8% and 13.1% populations, see Figure 3 and Supporting Information, Figure S1) above the population of 5%. They differed in the orientation of the methoxymethyl and C-12 acetoxy groups and only slightly in the conformation of the skeleton. It is also noteworthy that the C-12 and C-14 acetoxy groups of **1** adopted the usually unfavorable near *syn-antiperiplanar* conformation for the methine proton and the carbonyl oxygen in all the conformers (projection angles ω_12H,12C,C═O,C═O_ = 160.7°, ω_14H,14C,C═O,C═O_ = 163.8° in the lowest-energy conformer). In contrast, the 2- and 9-acetoxy groups of **1** have the expected near *syn-coplanar* conformation for the methine proton and the carbonyl oxygen (projection angles ω_2H,2C,C═O,C═O_ = 12.1°, ω_9H,9C,C═O,C═O_ = −12.4° in the lowest-energy conformer). The unexpected conformation of the ester groups in **1** is due to steric crowding, which may lead to erroneous configurational assignment by the Mosher’s MTPA or MPA ester NMR method when not supported by a careful conformational analysis. The interatomic distances observed in the calculated conformers corroborated well the observed NOE effects and thus the relative configuration (Figure 2). The 2-OAc group has *pseudoequatorial* and 9-OAc *pseudoaxial* orientation in agreement with the measured coupling constants of H-2 (*J* = 9.7 Hz) and H-9 (*J* = 4.6 Hz). The 8-OH was hydrogen-bonded to the epoxide oxygen. The ECD spectra were then calculated for the four conformers with three different functionals (B3LYP, BH&HLYP, PBE0) and 6-311G(d,p) basis set. The four conformers gave nearly identical ECDs and the three functionals consistently reproduced the experimental ECD with BH&HLYP showing the best agreement (Figure 4). Since the Boltzmann-weighted BH&HLYP/6-311G(d,p) ECD spectrum calculated for the (1*R*,2*S*,7*S*,8*S*,9*S*,10*S*,11*R*,12*R*,14*S*,17*R*)-enantiomer gave a good agreement with the experimental solution ECD, the absolute configuration of gemmacolide N (**1**) was unambiguously determined as identical to the calculated value.

Comparison of the ^1^H and ^13^C NMR spectra of gemmacolides O–S (**2**–**6**) with those of **1** immediately revealed that they are 11,20-epoxy-3,5-diene-type briarane analogues. Their structural determinations were aided by comparison of their spectra with those of **1**. However, detailed NMR studies (including HSQC, HMBC, ^1^H-^1^H COSY and NOESY spectra) on each new compound were performed in order to unambiguously determine their structures and to assign all the proton and carbon resonances. As the briarane derivatives **2**–**10** contained the same lactone and diene chromophores as gemmacolide N (**1**) and they differed only in the nature of the ester groups and R_5_, the ECD spectrum of gemmacolide N (**1**) could be used as an ECD reference for the configurational assignment of derivatives **2**–**10**.

**Figure 5.**
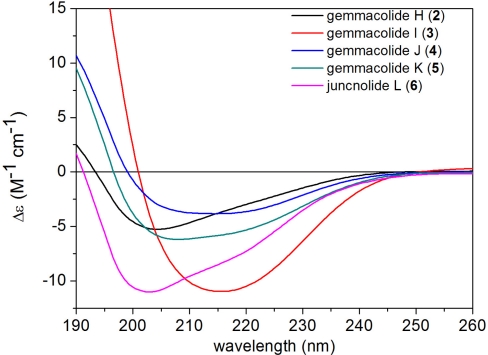
ECD spectra of gemmacolides O–S (**2**–**6**) in acetonitrile.

Gemmacolide O (**2**), a white amorphous powder (1.4 mg), had a molecular formula of C_30_H_37_O_15_Cl as established by HRESI-MS. An isotopic ratio of 3:1 observed in the molecular ion peak at *m/z* 695/697 ([M + Na]^+^) confirmed the appearance of a chlorine atom in the molecule, and the ^1^H and ^13^C NMR spectra data (Tables 1 and 2) of **2** resembled those of **7**. The C-2 glycoloyl group of **2** (δ_H_ 4.15, d, *J* = 16.9 Hz and 4.03, d, *J* = 16.9 Hz; δ_C_ 172.2, s, 61.1, t) was distinct from the analogously positioned acetoxy group of **7** (δ_H_ 1.96, s; δ_C_ 168.7, s and 21.3, q). In addition, the upfield-shifted C-16 signal of **2** (δ_C_ 44.5) in the ^13^C-NMR spectrum compared to that of **7** (δ_C_ 72.2) together with the absence of the methoxy group (δ_H_ 3.46, δ_C_ 58.5) indicated the position of a chlorine atom at C-16 [32]. The location of the glycoloyl group at C-2 was confirmed by the HMBC correlations of both H-2′ and H-2 with C-1′. The relative configuration of **2** was also proved the same as that of **7** by a NOESY experiment. Similarly to gemmacolide N (**1**), gemmacolide O (**2**) gave a negative ECD band at 202 nm and a positive one below 200 nm (Figure 5) and thus its absolute configuration was determined as (−)-(1*S*,2*S*,3*Z*,5*E*,7*S*,8*S*,9*S*,10*S*,11*R*,12*R*,13*R*,14*R*,17*R*) by its ECD spectrum.

Gemmacolide P (**3**) was obtained as a white amorphous powder (1.7 mg). Its HRESI-MS demonstrated the same molecular formula as C_33_H_44_O_15_ by HRESI-MS. ^1^H and ^13^C NMR spectroscopic data of **3** were almost identical to those of **10** except for the absence of the signals of the oxygenated methyl group (δ_H_ 3.45, s; δ_C_ 58.5, q). This fact suggested a primary hydroxyl group at C-16 in **3**. Detailed analysis of ^1^H-^1^H COSY and HMBC spectra clarified the isovaleryl group attached to C-12 in **3**. The structure of **3** was thus determined as (−)-(1*S*,2*S*,3*Z*,5*E*,7*S*,8*S*,9*S*,10*S*,11*R*,12*R*,13*R*,14*R*,17*R*) based on the ECD spectrum (Figure 5).

Gemmacolide Q (**4**) was isolated as a white amorphous powder (2.0 mg) and found to have a molecular formula of C_33_H_44_O_16_ established by HRESI-MS. ^1^H and ^13^C NMR spectra of **4** were similar to those of compound **3** but one of the acetyl groups in **3** was replaced by a glycoloyl group in **4**. This hydroxylated acetyl group was attached at C-2 as supported by the HMBC correlations of H-2′ and H-2 with C-1′. The relative and absolute configuration of **4** was shown to be the same as that of **3** by the analysis of NOESY and ECD spectra (Figure 5).

Gemmacolide R (**5**), a white amorphous powder (1.4 mg), showed the same molecular formula of C_33_H_44_O_16_ as that of **4** as deduced from its HRESI-MS. ^1^H and ^13^C NMR spectroscopic data of **5** were almost identical to those of **4** (Tables 1 and 2), showing the same functional groups for both compounds. The isovaleryl group, however, was found to be attached to C-14 of **5** instead of C-12 based on the analysis of HMBC spectra. The structure of **5** had the same relative and absolute stereochemistry as that of **4** assessed by the NOESY and ECD measurements (Figure 5).

Gemmacolide S (**6**) was obtained as a white amorphous powder (2.1 mg) with a molecular formula of C_43_H_60_O_18_ as established by HRESI-MS. Comparison of overall ^1^H and ^13^C NMR data of **6** and those of **2** revealed similarities (Tables 1 and 2). However, the primary alcohol of the glycoloyl group in **2** was further isovalerylated in **6** while the two acetyl groups at C-12 and C-14 remained intact due to the obvious HMBC correlations from the secondary alcohol protons to the respective ester carbonyl groups. The presence of two isovaleryl groups were assigned at C-13 and C-16 by the HMBC correlations from H-13 and H_2_-16 to the carbonyl atom of both isovaleryl groups [δ_C_ 171.7 (s), 172.0 (s)], respectively. The relative and absolute configuration of all the chiral centers was found the same as that of **2** by the NOESY and ECD analysis (Figure 5).

**Figure 6.**
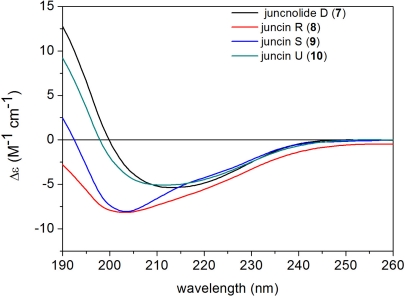
ECD spectra of juncnolide D, juncins R, S and U (**7**–**10**) in acetonitrile.

In briarane-type diterpenoids, the flexible bicyclo[8.4.0]tetradecane skeleton [3] and the complexity due to substitution pattern lead a great challenge in the stereochemical studies of the chiral centers. In this paper, the absolute configuration of **1** was solved by a TDDFT calculation of the solution ECD spectrum. The measured negative ECD band at about 215 nm and the positive one below 200 nm were correlated with (1*S*,2*S*,7*S*,8*S*,9*S*,10*S*,11*R*,12*R*,13*R*,14*R*,17*R*) absolute configuration as proved by TDDFT ECD calculations. The example of gemmacolide N (**1**) serves as a reference for the configurational assignment of related compounds considering the similar ECD curves (Figures 5 and 6) recorded for the new analogues **2**–**6** and the known compounds **7**–**10** (Figures 5 and 6), respectively. The research work may hold as a basis for the determination of absolute configuration for such complex marine natural products using an ECD method.

The cytotoxic activity and antimicrobial activity of compounds **1**–**10** were evaluated. In *in vitro* bioassays, compounds **4**, **5**, **6**, **8** and **9** showed cytotoxicity against human lung adenocarcinoma cells (A549) and human osteosarcoma cell (MG63). Compound **7** showed cytotoxicity against A549 and no activity toward MG63. Compounds **1**, **3**, and **10** showed no cytotoxic activity toward A549 and MG63 at 50.5, 44.1 and 43.2 μM, respectively (Table 3). Compounds **1**, **4**, **7**–**10** exhibited weak antifungal activity against *Septoria tritici* while compounds **1**, **2**, **4**, **7**–**10** showed potential antibacterial activity against the Gram negative bacterium *Escherichia coli*. None of the compounds were active toward the fungus *Microbotryum violaceum* and the Gram positive bacterium *Bacillus megaterium* (Table 4).

## 3. Experimental Section

### 3.1. General Experimental Procedures

Commercial silicagel (Yantai, China, 200–300; 400–500 mesh) and RP silicagel (Merck, Germany, 43–60 μm) were used for column chromatography (CC). Precoated silicagel plates (Yantai, China, HSGF-254) and RP silicagel (Macherey-Nagel, Germany, RP-18 F254) were used for analytical thin-layer chromatography (TLC). Spots were detected on TLC under UV or by heating after spraying with anisaldehyde-sulphuric acid reagent. The NMR spectra were recorded at 300 K on a Bruker drx 400 spectrometer. Chemical shifts are reported in parts per million (δ), with use of the residual CHCl_3_ signal (δ_H_ 7.26 ppm) as an internal standard for ^1^H NMR and CDCl_3_ (δ_C_ 77.0 ppm) for ^13^C NMR; Coupling constants (*J*) are reported in Hz. ^1^H NMR and ^13^C NMR assignments were complemented ^1^H-^1^H COSY, HSQC, HMBC and NOESY experiments. The following abbreviations are used to describe spin multiplicity: s = singlet, d = doublet, t = triplet, q = quartet, m = multiplet, br s = broad singlet, dd = doublet of doublets, ov = overlaped signals. Optical rotations were measured in CHCl_3_ with an Autopol IV polarimeter at the sodium D line (590 nm). Infrared spectra were recorded in thin polymer films on a Nexus 470 FT-IR spectrophotometer (Nicolet, USA); peaks are reported in cm^−1^. UV absorption spectra were recorded on a Varian Cary 100 UV-Vis spectrophotometer; peaks wavelengths are reported in nm. Circular dichroism spectra were recorded with a JASCO J-715 circular dichroism spectropolarimeter. The MS and HRMS were performed on a Q-TOF Micro mass spectrometer, resolution 5000. An isopropyl alcohol solution of sodium iodide (2 mg/mL) was used as a reference compound. Semi-preparative RP-HPLC was performed on an Agilent 1100 system equipped with a refractive index detector using an YMC Pack ODS-A column (particle size 5 μm, 250 × 10 mm).

### 3.2. Animal Material

The gorgonian coral *Dichotella gemmacea* (3.5 kg, wet weight) was collected from the South China Sea, in August 2007 and identified by Dr. Xiu-Bao Li, the South China Sea Institute of Oceanology, Academia Sinica. A voucher specimen was deposited in the Second Military Medical University, Shanghai, China.

### 3.3. Extraction and Isolation

The frozen specimen was extracted with acetone and methanol (3 × 1.5 L) by ultrasonication. The solvent was combined and removed *in vacuo*. The resultant residue was partitioned between H_2_O and EtOAc. The layers were separated, and then EtOAc was removed to afford 16.1 g of residue. The crude extract was further partitioned between MeOH and hexane, affording 11.2 g from the MeOH fraction. This residue was subjected to silica gel CC and eluted with hexane/acetone (from 100:0 to 0:100) as eluent. Fraction 13 was further fractionated by RP-silical gel CC (MeOH/H_2_O, 23:77 to 67:33, in 5% increments) to give three subfractions (A–C). Subfraction A was purified by HPLC (MeOH/H_2_O, 60:40, 1.5 mL min^−1^) to yield **1** (1.1 mg, 25.6 min) and **7** (2.1 mg, 28.7 min). Fraction 12 was further fractionated by silicagel CC (*n*-hexane/acetone, 80:20 to 50:50, in 2.5% increments) to give three subfractions (A–C). Subfraction A was purified by HPLC (MeOH/H_2_O, 65:35, 1.5 mL min^−1^), yielding **3** (1.7 mg, 37.5 min). Fraction 9 was purified by HPLC (MeOH/H_2_O, 67:33, 1.5 mL min^−1^) to give **2** (1.4 mg, 27.2 min), **5** (1.4 mg, 30.2 min) and **4** (2.0 mg, 32.1 min). Fraction 8 and 7 were repeatedly subjected to silicagel and Sephadex LH-20 CC, and then purified by HPLC (MeOH/H_2_O, 70:30, 1.5 mL min^−1^) to yield **8** (3.3 mg, 29.7 min), **9** (3.1 mg, 32.3 min), **10** (3.1 mg, 43.9 min). Fraction 4 was further fractionated by RP-silical gel CC (gradient elution from MeOH/H_2_O, 3:7 to MeOH, in 5% increments) and purified by HPLC (MeOH/H_2_O, 80:20, 1.5 mL min^−1^), yielding **6** (2.1 mg, 38.7 min).

**Gemmacolide N (1)**: white amorphous powder; ${\left[\alpha \right]}_{D}^{24}$ = 0 (*c* 0.035, CHCl_3_); UV (MeOH) 207, 273 nm; CD (CH_3_CN, *c* 4.2 × 10^−4^) λ_max_ (Δɛ) positive below 200 nm, 205 (−1.45), 223 (−1.35) nm; IR (film) ν_max_ 3475, 1776, 1739 cm^−1^; ^1^H NMR spectroscopic data, see Table 2; ^13^C NMR spectroscopic data, see Table 1; ESI-MS *m/z* 617 [M + Na]^+^; HRESI-MS *m/z* 617.2213 [M + Na]^+^ (calcd for C_29_H_38_O_13_Na, 617.2210).

**Gemmacolide O (2)**: white amorphous powder; ${\left[\alpha \right]}_{D}^{24}$ = − 30 (*c* 0.04, CHCl_3_); UV (MeOH) 206 nm; CD (CH_3_CN, *c* 4.3 × 10^−4^) λ_max_ (Δɛ) positive below 193 nm, 202 (−6.47) nm; IR (film) ν_max_ 3469, 1776, 1742 cm^−1^; ^1^H NMR spectroscopic data, see Table 2; ^13^C NMR spectroscopic data, see Table 1; ESI-MS *m/z* 695 [M + Na]^+^; HRESI-MS *m/z* 695.1721 [M + Na]^+^ (calcd for C_30_H_37_O_15_ClNa, 695.1719).

**Gemmacolide P (3)**: white amorphous powder; ${\left[\alpha \right]}_{D}^{24}$ = − 16 (*c* 0.08, CHCl_3_); UV (MeOH) 204 nm; CD (CH_3_CN, *c* 1.2 × 10^−4^) λ_max_ (Δɛ) positive below 200 nm, 216.5 (−12.77) nm; IR (film) ν_max_ 3479, 1775, 1743 cm^−1^; ^1^H NMR spectroscopic data, see Table 2; ^13^C NMR spectroscopic data, see Table 1; ESI-MS *m/z* 703 [M + Na]^+^; HRESI-MS *m/z* 703.2580 [M + Na]^+^ (calcd for C_33_H_44_O_15_Na, 703.2578).

**Gemmacolide Q (4)**: white amorphous powder; ${\left[\alpha \right]}_{D}^{24}$ = − 17 (*c* 0.065, CHCl_3_); UV (MeOH) 204 nm; CD (CH_3_CN, *c* 7.2 × 10^−4^) λ_max_ (Δɛ) positive below 199 nm, 217.5 (−4.22) nm; IR (film) ν_max_ 3456, 1772, 1742 cm^−1^; ^1^H NMR spectroscopic data, see Table 2; ^13^C NMR spectroscopic data, see Table 1; ESI-MS *m/z* 719 [M + Na]^+^; HRESI-MS *m/z* 719.2533 [M + Na]+ (calcd for C_33_H_44_O_16_Na, 719.2527).

**Gemmacolide R (5)**: white amorphous powder; ${\left[\alpha \right]}_{D}^{24}$ = − 18 (*c* 0.05, CHCl_3_); UV (MeOH) 204 nm; CD (CH_3_CN, *c* 3.3 × 10^−4^) λ_max_ (Δɛ) positive below 197 nm, 200.5 (−7.46) nm; IR (film) ν_max_ 3476, 1772, 1741 cm^−1^; ^1^H NMR spectroscopic data, see Table 2; ^13^C NMR spectroscopic data, see Table 1; ESI-MS *m/z* 719 [M + Na]^+^; HRESI-MS *m/z* 719.2529 [M + Na]^+^ (calcd for C_33_H_44_O_16_Na, 719.2527).

**Gemmacolide S (6)**: white amorphous powder; ${\left[\alpha \right]}_{D}^{24}$ = − 71 (*c* 0.055, CHCl_3_); UV (MeOH) 206 nm; CD (CH_3_CN, *c* 1.4 × 10^−4^) λ_max_ (Δɛ) positive below 191 nm, 197.5 (−15.11) nm; IR (film) ν_max_ 3478, 1778, 1744 cm^−1^; ^1^H NMR spectroscopic data, see Table 2; ^13^C NMR spectroscopic data, see Table 1; ESI-MS *m/z* 887 [M + Na]^+^; HRESI-MS *m/z* 887.3676 [M + Na]^+^ (calcd for C_43_H_60_O_18_Na, 887.3677).

**Juncenolide D (7)**: white amorphous powder; ${\left[\alpha \right]}_{D}^{24}$ = − 11 (*c* 0.10, CHCl_3_); CD (CH_3_CN, *c* 1.9 × 10^−4^) λ_max_ (Δɛ) positive below 200 nm, 214 (−6.26) nm.

**Juncin R (8)**: white amorphous powder; ${\left[\alpha \right]}_{D}^{24}$ = − 37 (*c* 1.14, CHCl_3_); CD (CH_3_CN, *c* 3.6 × 10^−4^) λ_max_ (Δɛ) 198 (−10.51) nm.

**Juncin S (9)**: white amorphous powder; ${\left[\alpha \right]}_{D}^{24}$ = − 33 (*c* 0.97, CHCl_3_); CD (CH_3_CN, *c* 1.8 × 10^−4^) λ_max_ (Δɛ) positive below 192 nm, 202 (−10.46) nm.

**Juncin U (10)**: white amorphous powder; ${\left[\alpha \right]}_{D}^{24}$ = − 20 (*c* 0.87, CHCl_3_); CD (CH_3_CN, *c* 1.8 × 10^−4^) *λ*_max_ (Δɛ) positive below 198 nm, 214 (−5.89) nm.

### 3.4. Computational Section

Mixed torsional/low mode conformational searches were carried out by means of the Macromodel 9.7.211 [33] software using Merck Molecular Force Field (MMFF) with implicit solvent model for chloroform. The MMFF analysis provided seventy two conformers in an 11 kJ/mol energy window, which were then reoptimized at the B3LYP/6-31G (d) level of theory affording four conformers. This was followed by TDDFT ECD calculations using various functionals (B3LYP, BH&HLYP, PBE0) and 6-311G (d,p) basis set implemented in the Gaussian 03 [34] package. Boltzmann distributions were estimated from the ZPVE corrected B3LYP/6-31G (d) energies of the optimized conformer geometries obtained at the same level of theory in the gas-phase calculations. ECD spectra were generated as the sum of Gaussians [35] with 3000 cm^−1^ half-height width (corresponding to ca. 15 nm at 220 nm), using dipole-velocity computed rotational strengths. The MOLEKEL [36] software package was used for visualization of the results.

### 3.5. Cytotoxicity Assay

Compounds **1**–**10** were evaluated for cytotoxicity against human lung adenocarcinoma (A549) and human osteosarcoma cell (MG63), using a modification of the 3-(4,5-dimethylthiazol-2-yl)-2, 5-diphenyltetrazolium bromide (MTT) colorimetric method [37]. Adriamycin was used as positive control.

### 3.6. Agar Diffusion Test for Biological Activity

Compounds **1**–**10** were dissolved in acetone at 2 mg/mL; 25 μL of the solution (0.05 mg) were pipetted onto a sterile filter disc (Schleicher & Schuell, 9 mm), which was placed onto an appropriate agar growth medium for the respective test organism and subsequently sprayed with a suspension of the test organisms. The test organisms were bacteria, gram-negative bacterium *Escherichia coli* and the gram positive bacterium *Bacillus megaterium* (both grown on NB medium), and fungi, *Microbotryum violaceum* and *Septoria tritici* (both grown on MPY medium). Reference substances were ketoconazole, penicillin and streptomycin. The radius of the zone of inhibition was measured in mm. These microorganisms were chosen because (a) they are non-pathogenic and (b) had in the past proved to be accurate initial test organisms for antibacterial and antifungal activities (each 0.05 mg). Commencing at the outer edge of the filter disc, the radius of the zone of inhibition was measured in mm.

## Supporting Information



## Figures and Tables

**Table 1.** ^13^C NMR data of gemmacolides N–S (**1**–**6**)^a^.

**Carbon**	**1**	**2**	**3**	**4**	**5**	**6**
1	47.1 s	46.5 s	46.5 s	46.6 s	46.6 s	46.4 s
2	74.3 d	75.2 d	75.6 d	77.2 d	76.7 d	75.5 d
3	131.6 d	131.1 d	131.2 d	130.7 d	130.6 d	131.4 d
4	128.3 d	129.1 d	129.6 d	130.1 d	130.1 d	128.5 d
5	141.8 s	139.7 s	144.7 s	144.2 s	144.3 s	139.7 s
6	122.4 d	126.3 d	123.7 d	123.9 d	123.6 d	122.4 d
7	79.0 d	78.5 d	78.7 d	78.7 d	78.3 d	78.6 d
8	81.1 s	81.0 s	81.0 s	80.1 s	80.1 s	81.1 s
9	64.0 d	63.6 d	63.8 d	63.8 d	63.8 d	63.8 d
10	32.8 d	32.6 d	32.8 d	32.7 d	32.6 d	32.6 d
11	59.0 s	58.1 s	58.4 s	58.2 s	58.3 s	58.4 s
12	73.1 d	73.0 d	72.8 d	72.7 d	73.1 d	73.2 d
13	28.5 t	66.4 d	66.4 d	66.3 d	66.4 d	66.4 d
14	73.6 d	73.8 d	73.7 d	73.8 d	73.5 d	73.8 d
15	14.0 q	14.3 q	14.5 q	14.4 q	14.5 q	14.5 q
16	72.3 t	44.5 t	63.9 t	63.8 t	63.8 t	62.7 t
17	44.2 d	44.0 d	44.1 d	44.1 d	44.1 d	44.1 d
18	175.6 s	174.9 s	175.2 s	175.2 s	175.1 s	175.2 s
19	6.3 q	6.3 q	6.3 q	6.3 q	6.3 q	6.3 q
20	49.0 t	48.9 t	49.0 t	49.1 t	49.1 t	48.8 t
9-OAc	170.2 s	170.1 s	170.2 s	170.2 s	170.2 s	170.2 s
	21.5 q	21.5 q	21.6 q	21.6 q	21.1 q	21.5 q
R_1_	169.2 s	172.2 s	170.9 s	172.9 s	172.4 s	166.6 s
	21.1 q	61.1 t	20.5 q	61.2 t	61.3 t	60.9 t
						172.4 s
						42.7 t
						25.6 d
						22.4 q (×2)
R_2_	170.0 s	169.6 s	see 1′–5′	see 1′–5′	169.8 s	169.7 s
	21.1 q	20.9 q			21.6 q	20.7 q
R_3_	n.o.	169.7 s	169.7 s	169.7 s	169.7 s	see 1′–5′
		20.5 q	21.5 q	20.5 q	20.6 q	
R_4_	169.8 s	170.6 s	170.1 s	170.7 s	see 1′–5′	170.5 s
	21.1 q	20.9 q	20.8 q	20.9 q		20.7 q
R_5_	58.4 q					see 1″–5″
1′			172.0 s	171.9 s	172.9 s	171.7 s
2′			43.5 t	43.4 t	43.1 t	42.6 t
3′			25.7 d	25.7 d	25.2 d	25.0 d
4′			22.3 q (×2)	22.4 q (×2)	22.5 q	22.3 q (×2)
5′					22.4 q	
1″						172.0 s
2″						43.3 t
3″						25.7 d
4″ and 5″						22.4 q (×2)

a 100 MHz, in CDCl_3_, assignments made by DEPT, HSQC, and HMBC.

**Table 2.** ^1^H NMR data for gemmacolides N–S (**1**–**6**)^a^.

**Proton**	**1**	**2**	**3**	**4**	**5**	**6**
2	5.71 (d, 9.7)	5.65 (ov)	5.63 (ov)	5.76 (d, 9.4)	5.69 (d, 9.6)	5.69 (ov)
3	5.60 (t, 10.5, 9.7)	5.64 (ov)	5.60 (ov)	5.63 (t, 9.4, 10.7)	5.63 (t, 9.6, 10.5)	5.63 (ov)
4	6.27 (d, 10.5)	6.42 (d, 9.7)	6.35 (d, 9.6)	6.39 (d, 10.7)	6.39 (d, 10.5)	6.34 (d, 10.6)
6	5.90 (d, 8.5)	6.07 (d, 9.0)	5.82 (d, 7.4)	5.86 (d, 8.9)	5.86 (d, 8.3)	5.69 (ov)
7	5.01 (d, 8.5)	4.95 (d, 9.0)	4.96 (d, 7.4)	4.95 (d, 8.9)	4.95 (d, 8.3)	4.96 (d, 8.6)
9	4.81 (br d, 4.6)	4.76 (br d, 4.5)	4.74 (br d, 4.8)	4.75 (br d, 4.5)	4.78 (br d, 4.7)	4.73 (br d, 4.9)
10	3.69 (br d, 4.6)	3.62 (ov)	3.61 (br d, 4.8)	3.63 (ov)	3.62 (ov)	3.63 (ov)
12	4.51 (ov)	4.88 (br d, 2.6)	4.92 (br d, 3.5)	4.93 (br d, 2.9)	4.90 (br d, 2.2)	4.88 (br s)
13*β*	1.92 (ov)	5.06 (dd, 2.6, 2.6)	5.09 (dd, 3.5, 3.5)	5.08 (dd, 2.9, 3.9)	5.09 (dd, 2.2, 2.2)	5.09 (br s)
13*α*	2.31 (ov)					
14	4.90 (br s)	5.17 (br d, 2.6)	5.23 (br d, 3.5)	5.19 (br d, 2.9)	5.24 (br d, 2.2)	5.20 (br s)
15	1.04 (s)	1.14 (s)	1.14 (s)	1.15 (s)	1.15 (s)	1.13 (s)
16a	4.48 (d, 15.2)	4.66 (d, 13.8)	4.51 (br s)	4.55 (d, 15.6)	4.54 (d, 15.0)	5.46 (d, 16.3)
16b	4.24 (d, 15.2)	4.56 (d, 13.8)		4.45 (d, 15.6)	4.47 (d, 15.0)	4.56 (d, 16.3)
17	2.32 (ov)	2.31 (q, 7.1)	2.30 (ov)	2.31 (ov)	2.31 (ov)	2.29 (ov)
19	1.16 (d, 7.1)	1.15 (ov)	1.13 (d, 7.2)	1.14 (d, 7.2)	1.16 (ov)	1.14 (d, 6.9)
20a	3.55 (br d, 2.4)	3.60 (ov)	3.63 (br d, 2.5)	3.62 (ov)	3.62 (br s)	3.61 (br s)
20b	2.78 (br d, 2.4)	2.93 (br s)	2.93 (br d, 2.5)	2.94 (br s)	2.93 (br s)	2.93 (br s)
9-OAc	2.19 (s)	2.19 (s)	2.19 (s)	2.20 (s)	2.19 (s)	2.18 (s)
R_1_	1.96 (s)	4.15 (d, 16.9)	1.99 (s)	4.15 (d, 17.2)	4.16 (d, 16.4)	4.52 (d, 15.5)
		4.03 (d, 16.9)		4.04 (d, 17.2)	4.03 (d, 16.4)	4.42 (d, 15.5)
						2.28 (ov)
						2.13 (ov)
						0.97 (d, 6.5) (×2 Me)
R_2_	2.10 (s)	2.16 (s)	see 2′–5′	see 2′–5′	2.17 (s)	2.16 (s)
R_3_	n.o.	1.95 (s)	1.94 (s)	1.94 (s)	1.94 (s)	see 2′–5′
R_4_	2.02 (s)	2.09 (s)	2.09 (s)	2.11 (s)	see 2′–5′	2.10 (s)
R_5_	3.45 (s)					see 2″–5″
2′a			2.35 (ov)	2.34 (m)	2.31 (m)	2.08 (ov) (×2)
2′b			2.27 (ov)	2.23 (m)	2.20 (m)	
3′			2.17 (m)	2.16 (m)	2.05 (m)	1.99 (m)
4′			0.99 (d, 6.3)	0.99 (d, 6.5)	0.98 (d, 7.1)	0.92 (d, 6.5) (×2 Me)
5′			1.01 (d, 6.3)	1.01 (d, 6.5)	1.00 (d, 7.1)	
2″						2.28 (ov)
3″						2.13 (ov)
4″ and 5″						0.98 (d, 6.1) (×2 Me)

a 400 MHz, in CDCl_3_, assignments made by ^1^H-^1^H COSY, HSQC, HMBC and NOESY.

**Table 3.** Cytotoxicity of compounds **1**–**10** against two tumor cell lines *in vitro* (IC_50_ in μM).

	**1**	**2**	**3**	**4**	**5**	**6**	**7**	**8**	**9**	**10**	**Adriamycin^a^**
A549	>50.5	>44.6	>44.1	21.6 ± 1.8	27.2 ± 2.4	16.4 ± 2.3	37.1 ± 4.2	13.9 ± 2.5	20.2 ± 2.3	>43.2	2.8 ± 0.32
MG63	>50.5	>44.6	>44.1	20.5 ± 2.1	23.7 ± 2.8	18.8 ± 3.9	>46.0	5.6 ± 1.2	16.5 ± 2.4	>43.2	3.2 ± 0.37

Results are presented as mean ± s.d. (*n* = 3).

a Positive control.

**Table 4.** Agar diffusion assays for antibacterial, and antifungal activities^a,b^.

	***M. violaceum***	***S. tritici***	***E. coli***	***B. megaterium***
**1**	0	7.5	12.5	0
**2**	6.0	6.5	13.0	6.0
**3**	6.0	6.5	7.5	5.5
**4**	6.5	7.5	10.0	5.5
**5**	0	5.5	5.5	6.0
**6**	7.0	0	6.0	0
**7**	6.0	7.5	12.5	6.0
**8**	7.5	7.5	14.0	6.0
**9**	5.5	7.0	10.0	0
**10**	0	7.5	11.0	8.0
penicillin	7.0	6.0	15.0	8.0
streptomycin	8.0	5.5	9.0	5.5
ketoconazole	15.0	12.5	9.0	11.0
acetone	0	0	0	0

a 0.05 mg of the test or control substances dissolved in acetone were applied to a filter disc and sprayed with the respective test organism.

b Radii of the zones of inhibition are given in mm.
