# Supplementary material for: Bioactive (3Z,5E)-11,20-Epoxybriara-3,5-dien-7,18-olide Diterpenoids from the South China Sea Gorgonian Dichotella gemmacea
Source: Mar Drugs. 2011 Aug 16;9(8):1403–18. doi: 10.3390/md9081403 (PMC3164382; doi:10.3390/md9081403)

Supporting Information

Index

1	Spectra of the new compound **1**.	S1–S8
	HR-ESIMS spectrum of the new compound **1**	S1
	^{1}H NMR spectrum of the new compound **1**	S2
	^{13}C NMR spectrum of the new compound **1**	S3
	DEPT spectrum of the new compound **1**	S4
	HSQC spectrum of the new compound **1**	S5
	^{1}H-^{1}H COSY spectrum of the new compound **1**	S6
	HMBC spectrum of the new compound **1**	S7
	NOESY spectrum of the new compound **1**	S8
2	Spectra of the new compound **2**	S9–S14
	HR-ESIMS spectrum of the new compound **2**	S9
	^{1}H NMR spectrum of the new compound **2**	S10
	^{13}C NMR spectrum of the new compound **2**	S11
	DEPT spectrum of the new compound **2**	S12
	HSQC spectrum of the new compound **2**	S13
	^{1}H-^{1}H COSY spectrum of the new compound **2**	S14
	HMBC spectrum of the new compound **2**	S15
	NOESY spectrum of the new compound **2**	S16
3	Spectra of the new compound **3**	S17–S24
	HR-ESIMS spectrum of the new compound **3**	S17
	^{1}H NMR spectrum of the new compound **3**	S18
	^{13}C NMR spectrum of the new compound **3**	S19
	DEPT spectrum of the new compound **3**	S20
	HSQC spectrum of the new compound **3**	S21
	^{1}H-^{1}H COSY spectrum of the new compound **3**	S22
	HMBC spectrum of the new compound **3**	S23
	NOESY spectrum of the new compound **3**	S24
4	Spectra of the new compound **4**	S25–S32
	HR-ESIMS spectrum of the new compound **4**	S25
	^{1}H NMR spectrum of the new compound **4**	S26
	^{13}C NMR spectrum of the new compound **4**	S27
	DEPT spectrum of the new compound **4**	S28
	HSQC spectrum of the new compound **4**	S29
	^{1}H-^{1}H COSY spectrum of the new compound **4**	S30
	HMBC spectrum of the new compound **4**	S31
	NOESY spectrum of the new compound **4**	S32
5	Spectra of the new compound **5**	S33–S38
	HR-ESIMS spectrum of the new compound **5**	S33
	^{1}H NMR spectrum of the new compound **5**	S34

	^{13}C NMR spectrum of the new compound **5**	S35
	DEPT spectrum of the new compound **5**	S36
	HSQC spectrum of the new compound **5**	S37
	^{1}H-^{1}H COSY spectrum of the new compound **5**	S38
	HMBC spectrum of the new compound **5**	S39
	NOESY spectrum of the new compound **5**	S40
6	Spectra of the new compound **6**	S41–S48
	HR-ESIMS spectrum of the new compound **6**	S41
	^{1}H NMR spectrum of the new compound **6**	S42
	^{13}C NMR spectrum of the new compound **6**	S43
	DEPT spectrum of the new compound **6**	S44
	HSQC spectrum of the new compound **6**	S45
	^{1}H-^{1}H COSY spectrum of the new compound **6**	S46
	HMBC spectrum of the new compound **6**	S47
	NOESY spectrum of the new compound **6**	S48
7	Figure S1. Four conformational isomers and populations of (1*R*,2*S*,7*S*,8*S*,9*S*, 10*S*,11*R*,12*R*, 14*S*,17*R*)-gemmacolide N (**1**) obtained by the B3LYP/6-31G(d) reoptimization of the seventy two MMFF conformers.	S49
8	Figures S2–S5, Individual calculated ECDs for four computed conformers used for the calculation of the Boltzmann-weighted ECD spectrum of **1**.	S50

S1. HR-ESIMS spectrum of the new compound **1**.

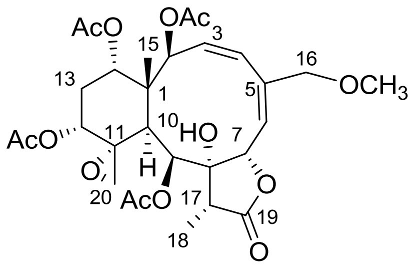

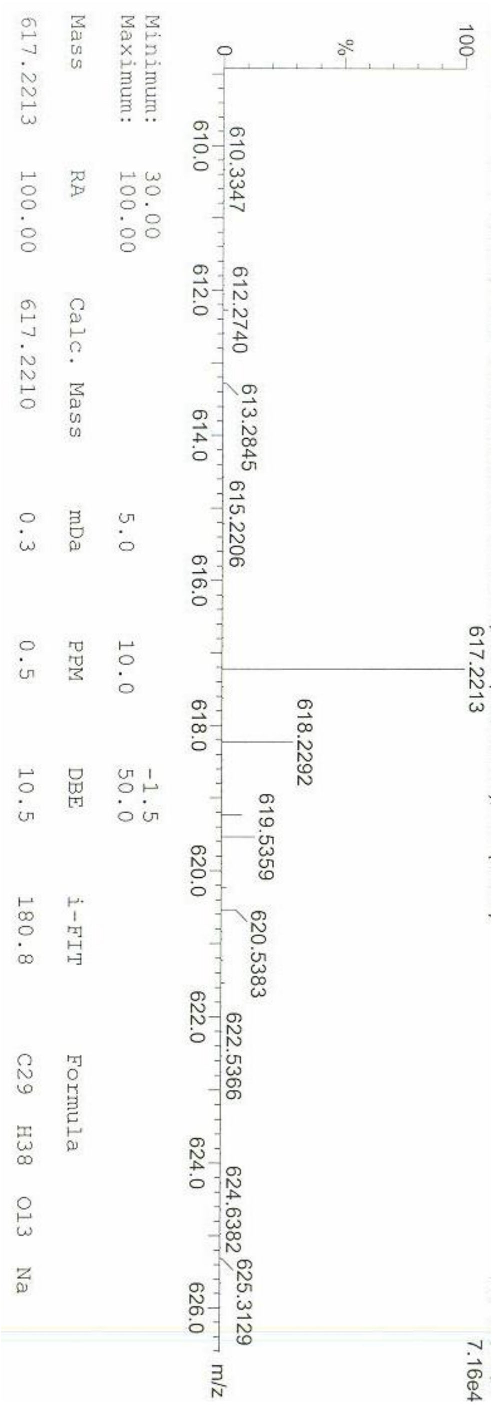

S2. ^{1}H NMR spectrum of the new compound **1**.

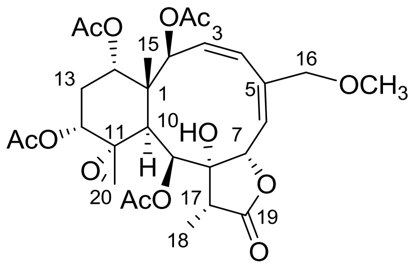

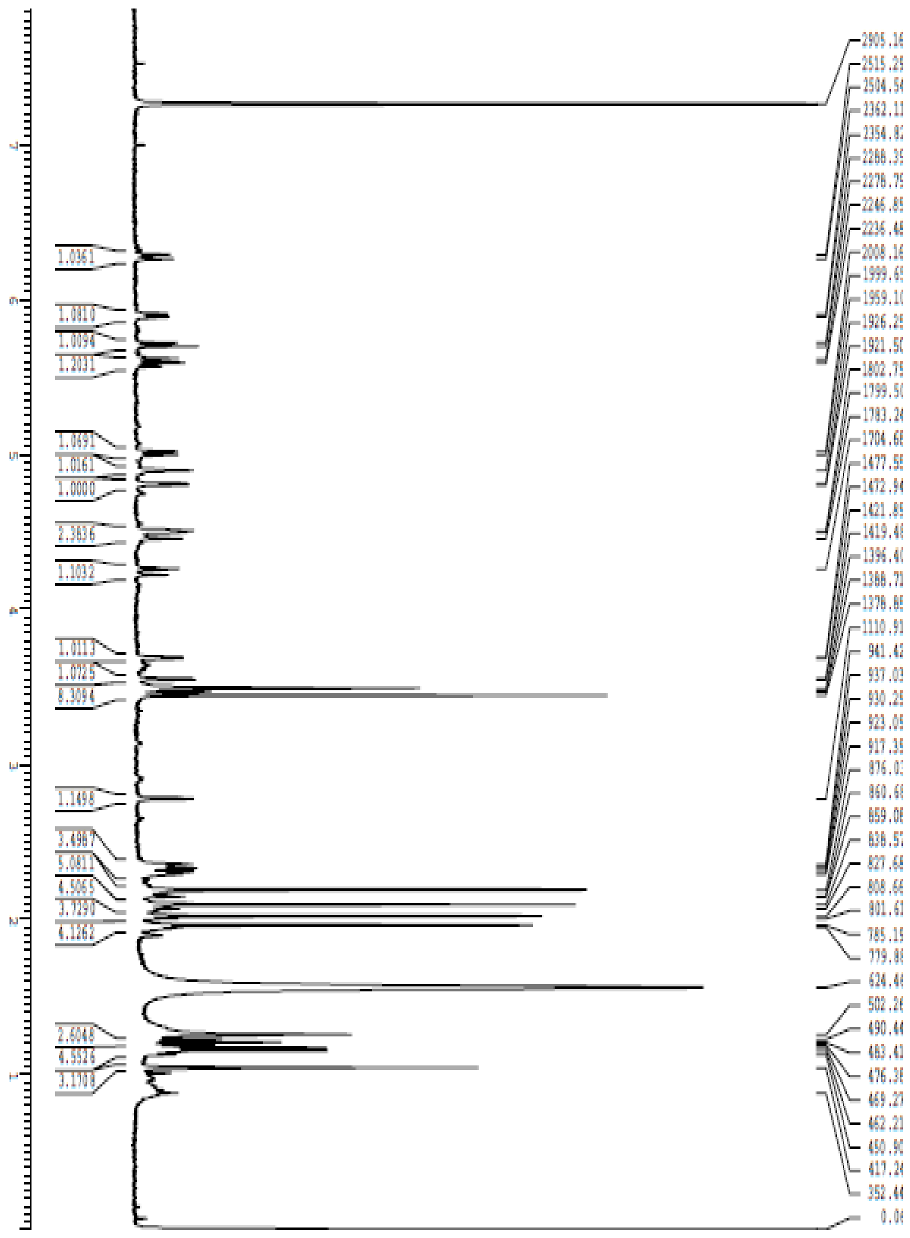

S3. ^{13}C NMR spectrum of the new compound **1**.

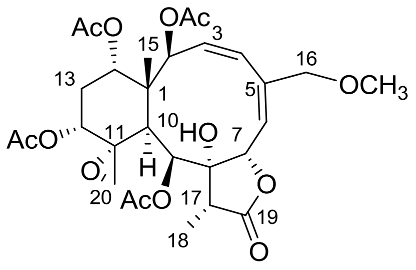

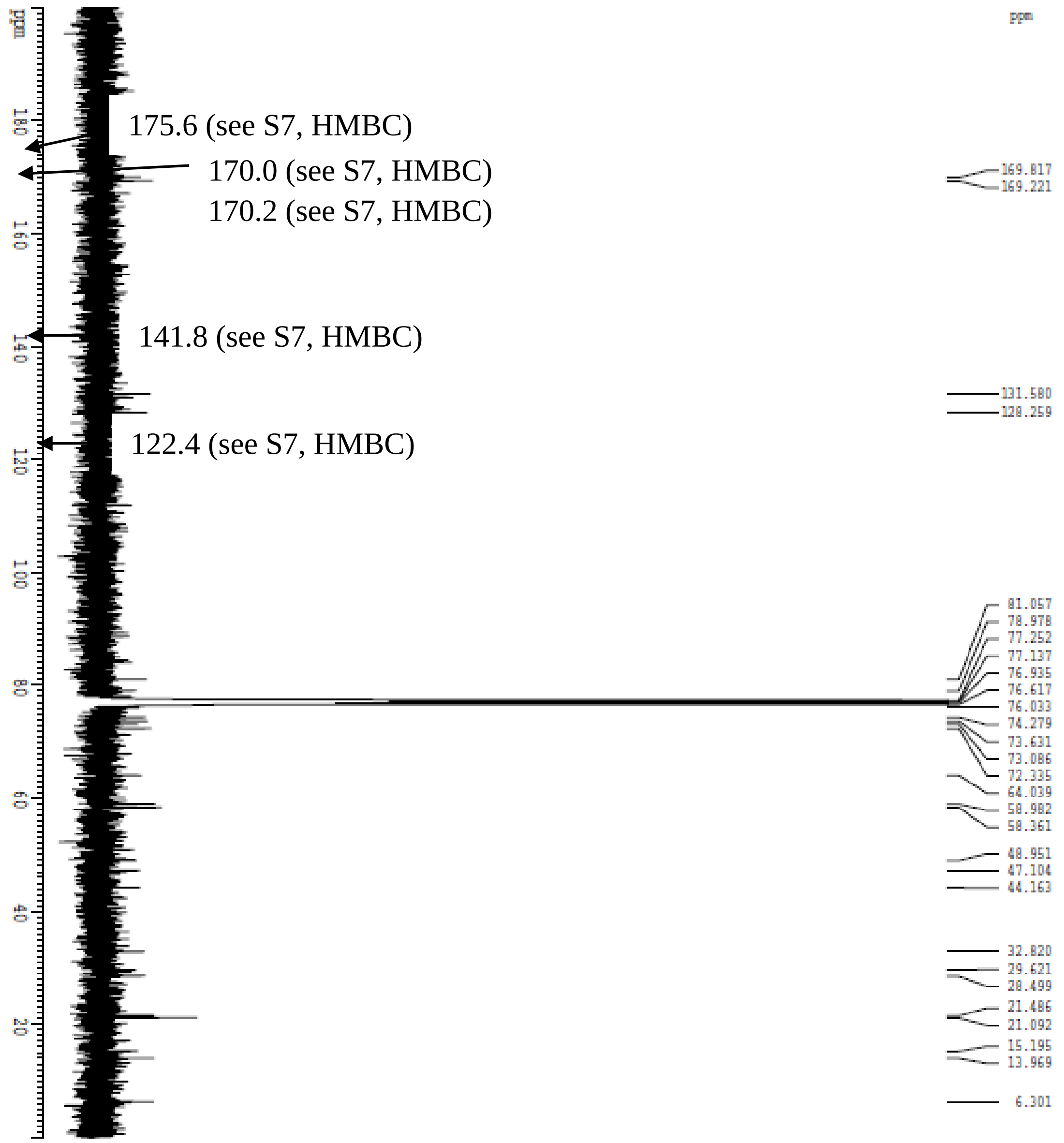

S4. DEPT spectrum of the new compound **1**.

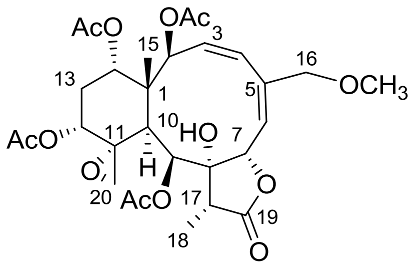

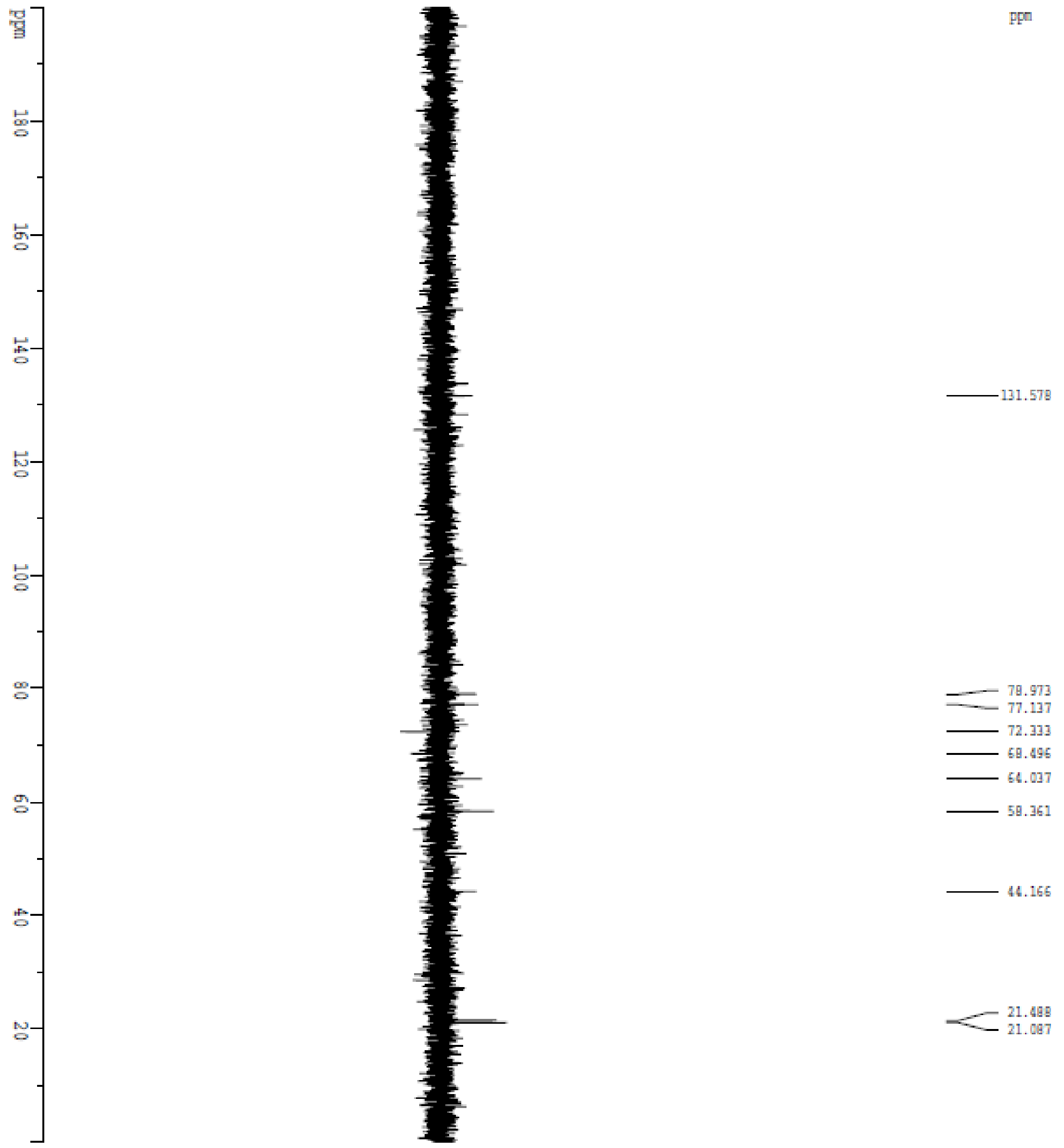

S5. HSQC spectrum of the new compound **1**.

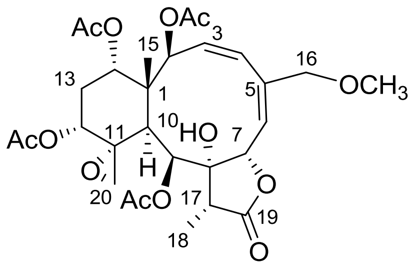

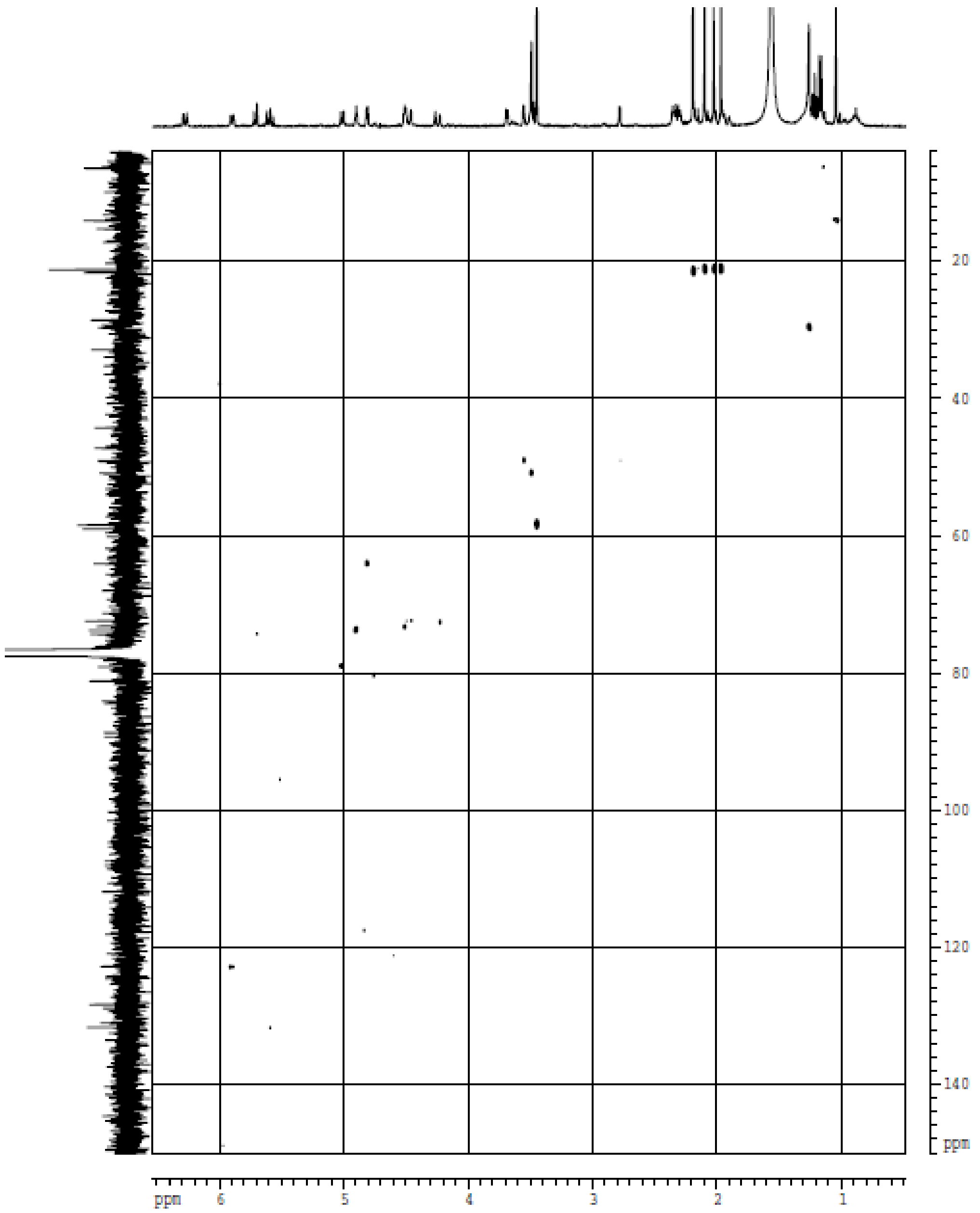

S6. ^{1}H-^{1}H COSY spectrum of the new compound **1**.

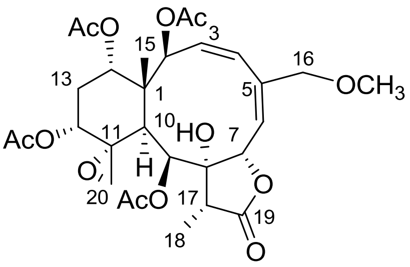

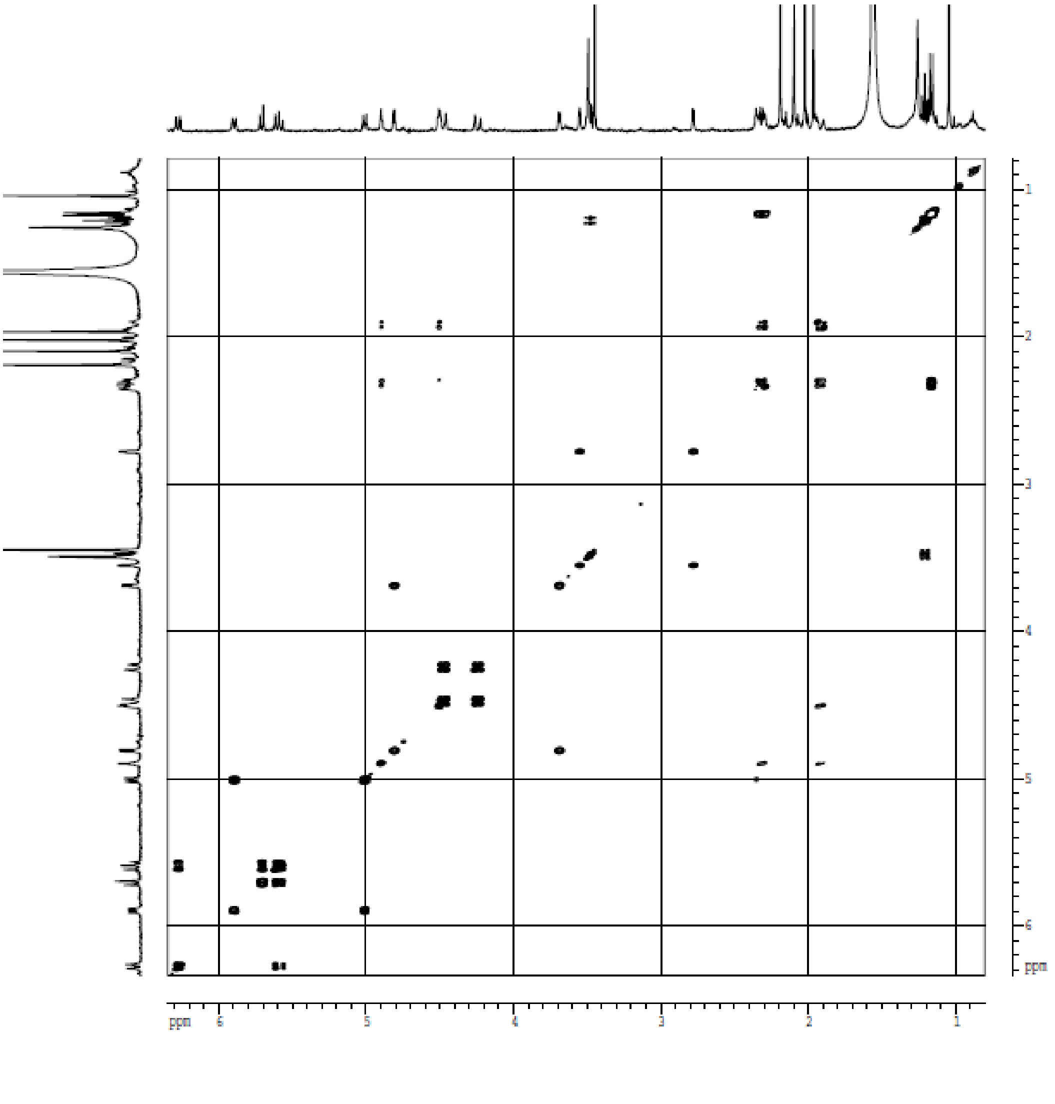

S7. HMBC spectrum of the new compound **1**.

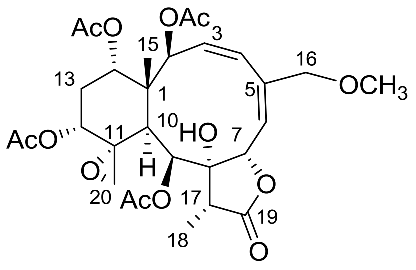

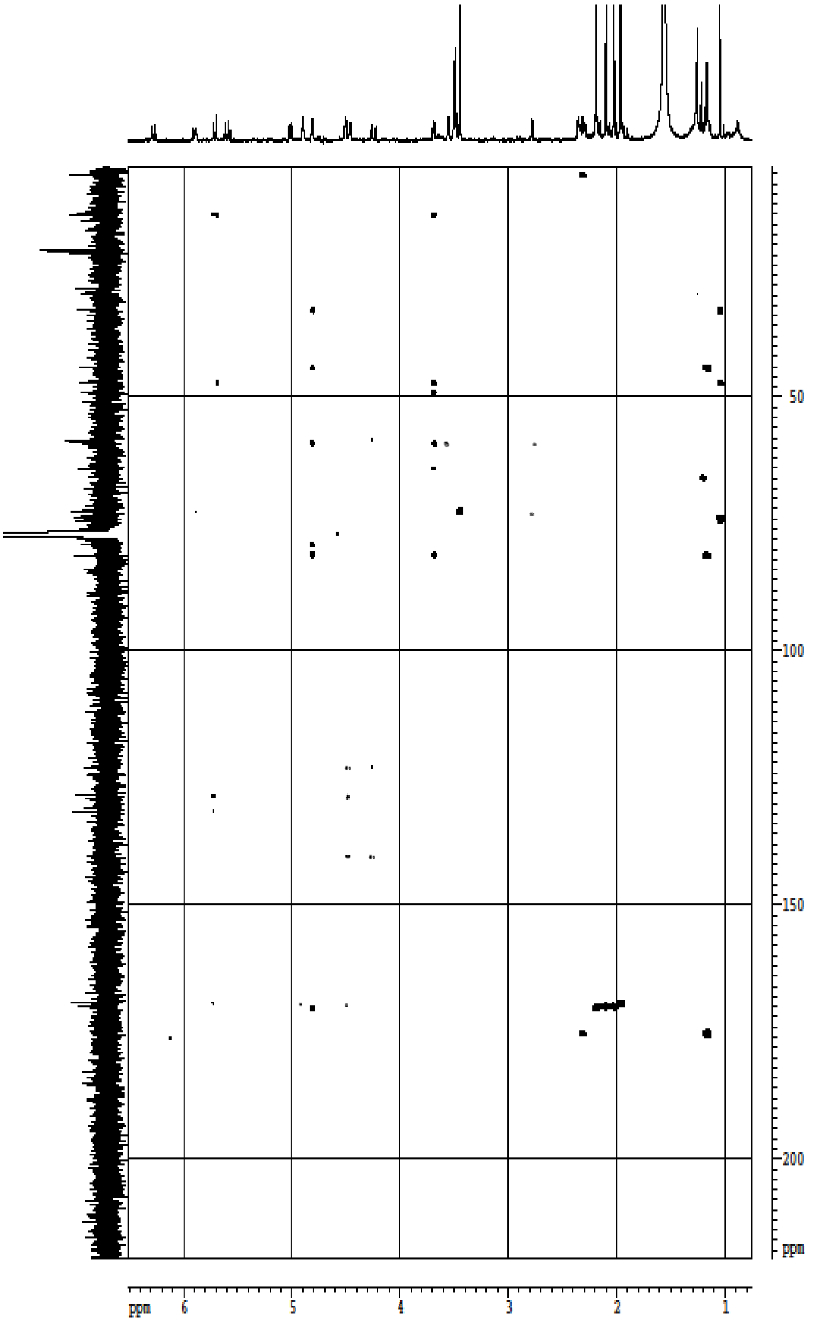

S8. NOESY spectrum of the new compound **1**.

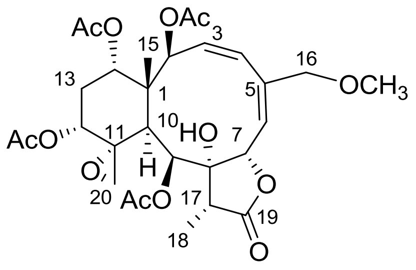

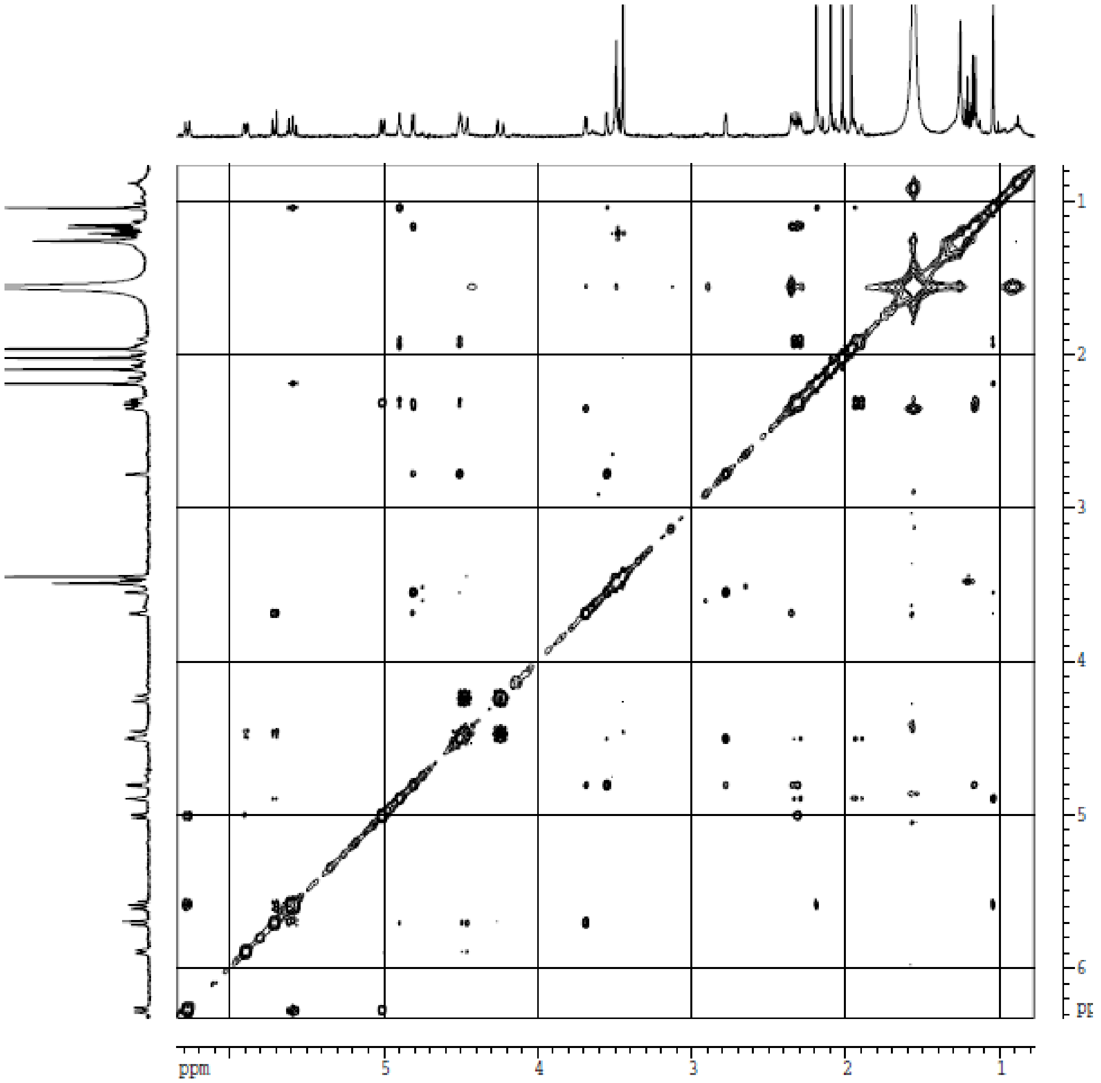

S9. HR-ESIMS spectrum of the new compound **2**.

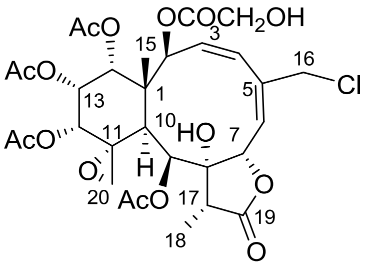

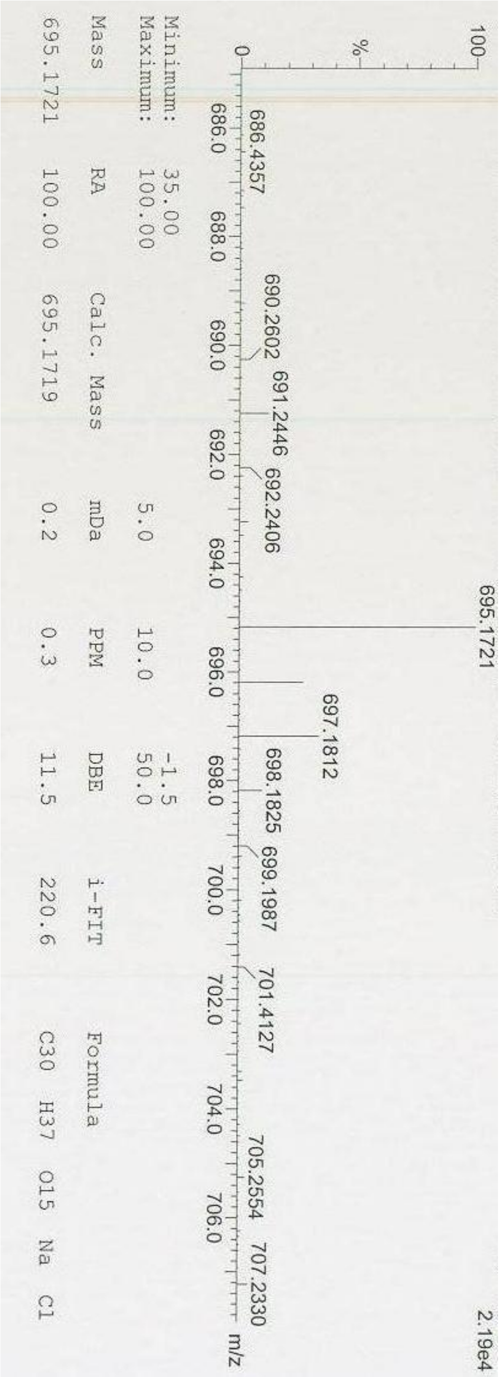

S10. ^{1}H NMR spectrum of the new compound **2**.

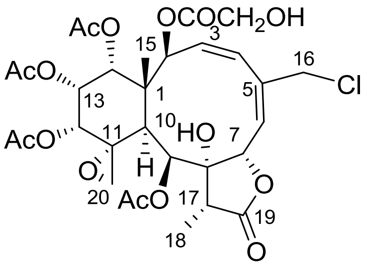

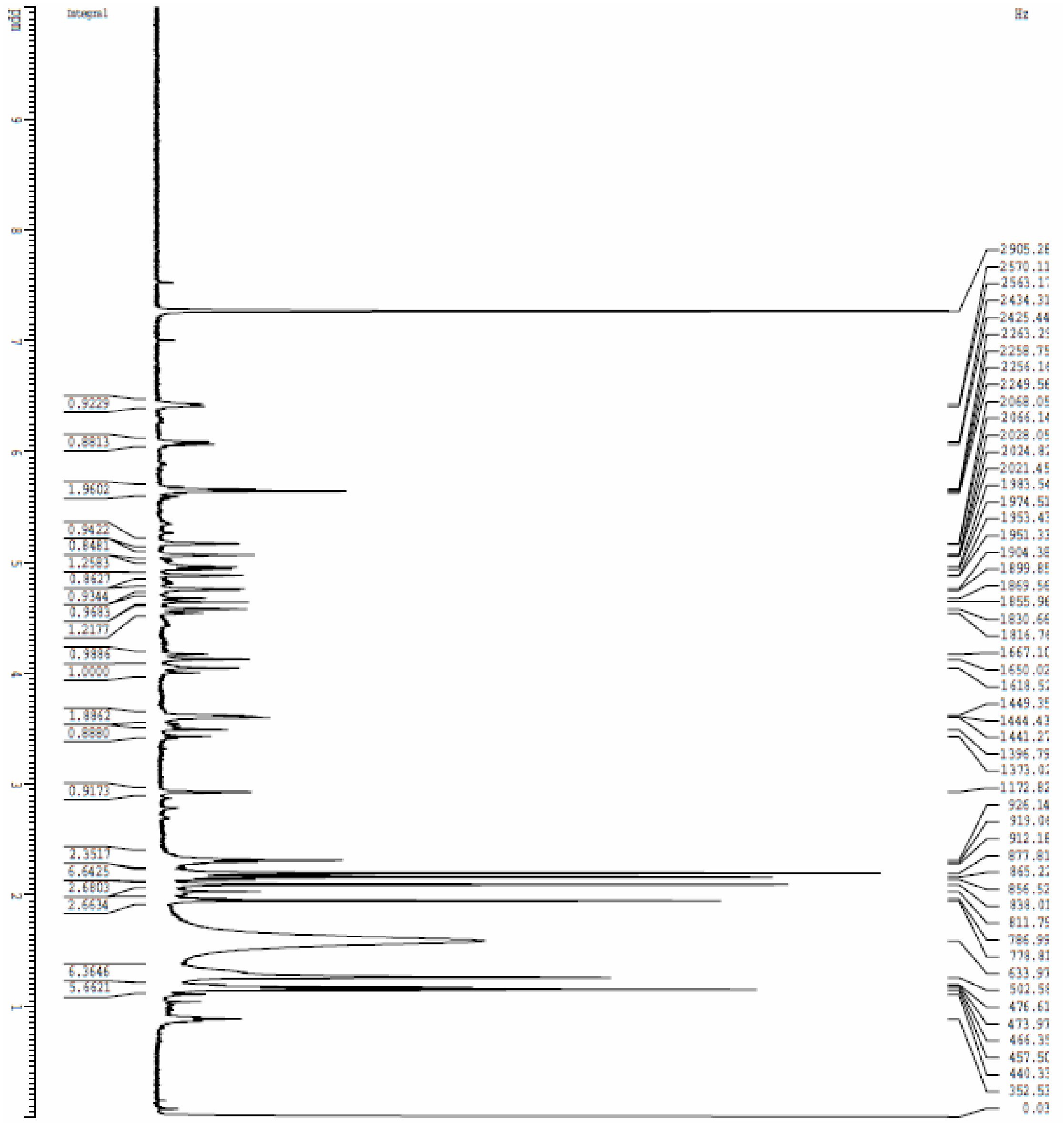

S11. ^{13}C NMR spectrum of the new compound **2**.

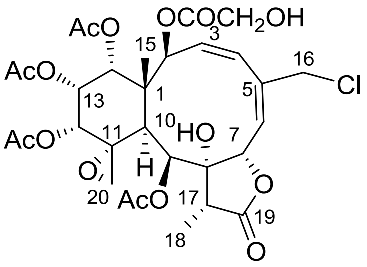

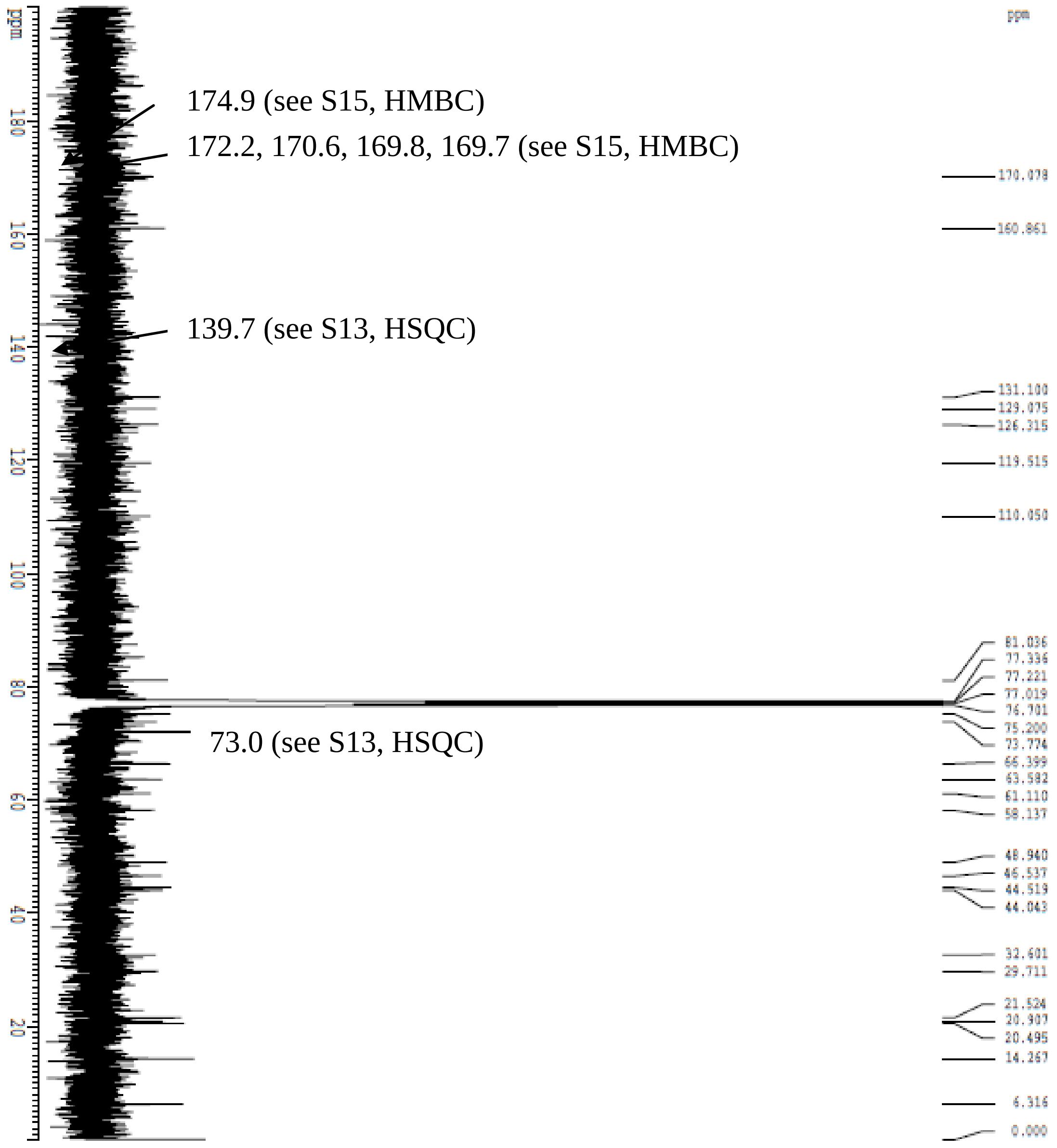

S12. DEPT spectrum of the new compound **2**.

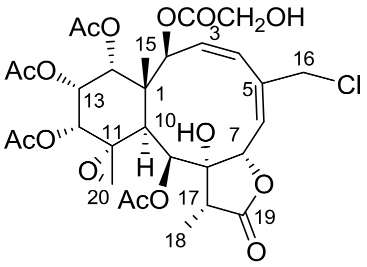

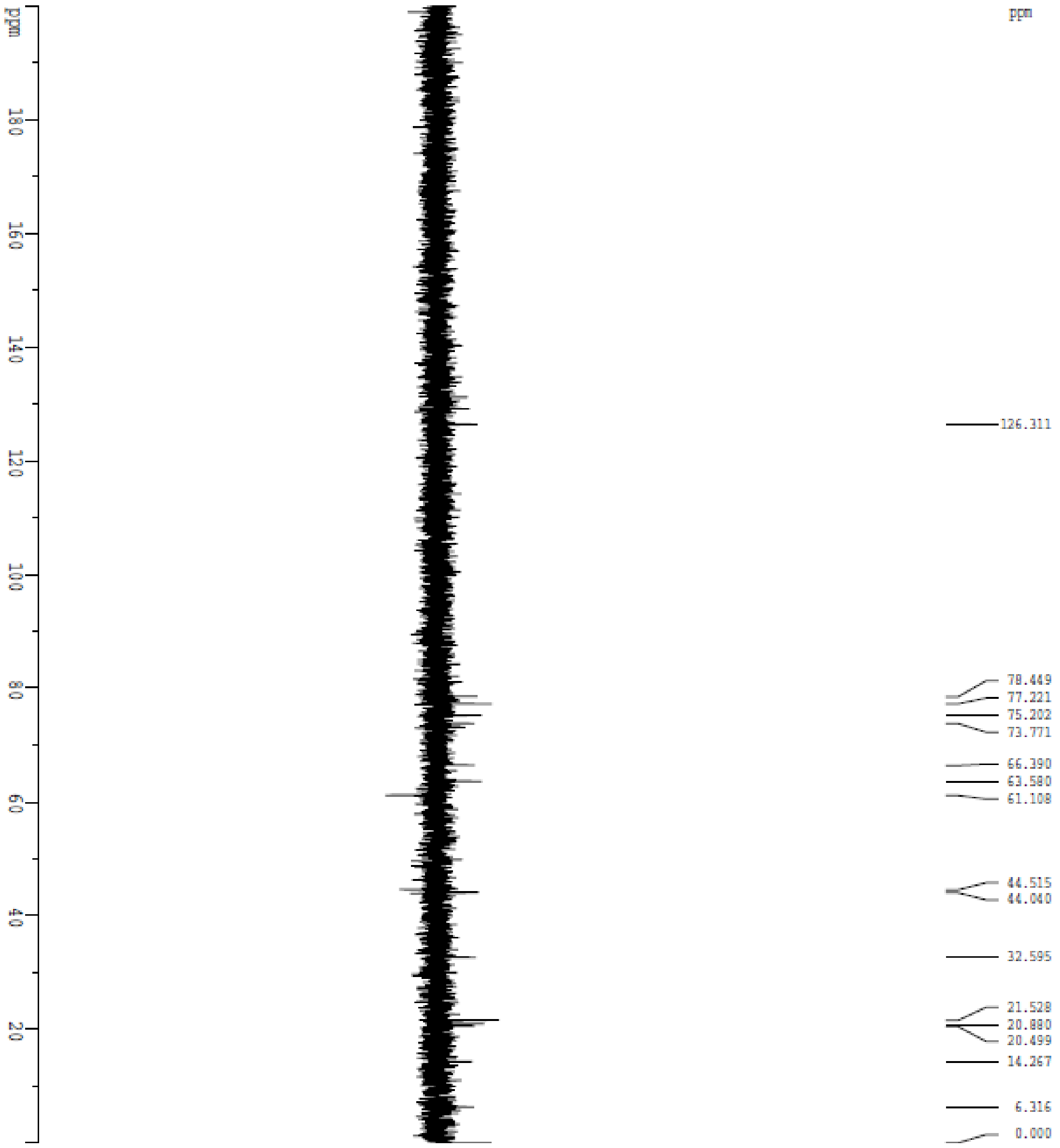

S13. HSQC spectrum of the new compound **2**.

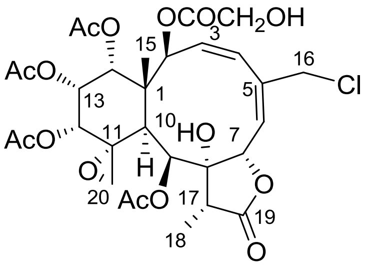

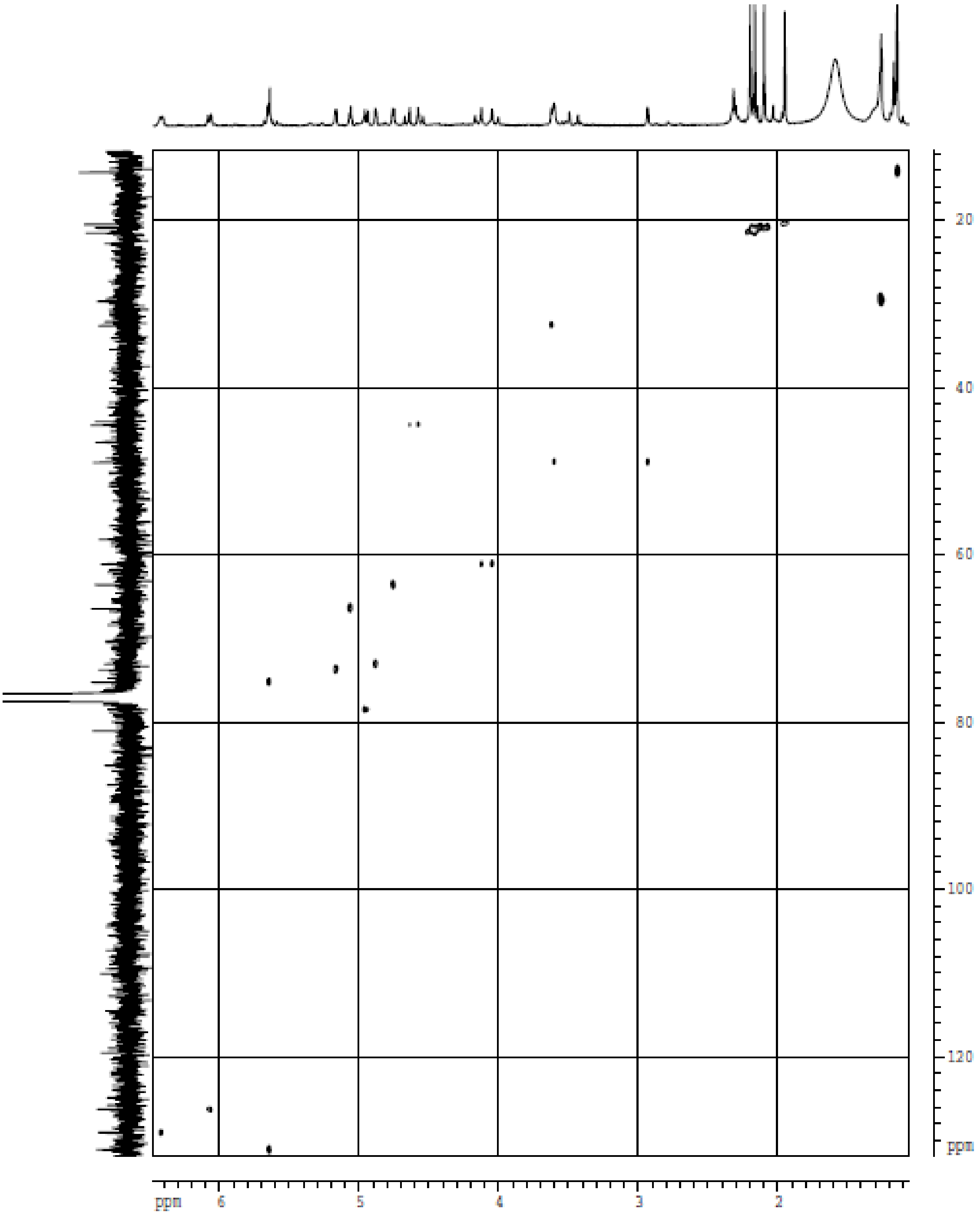

S14. ^{1}H-^{1}H COSY spectrum of the new compound **2**.

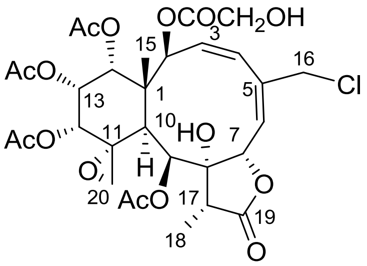

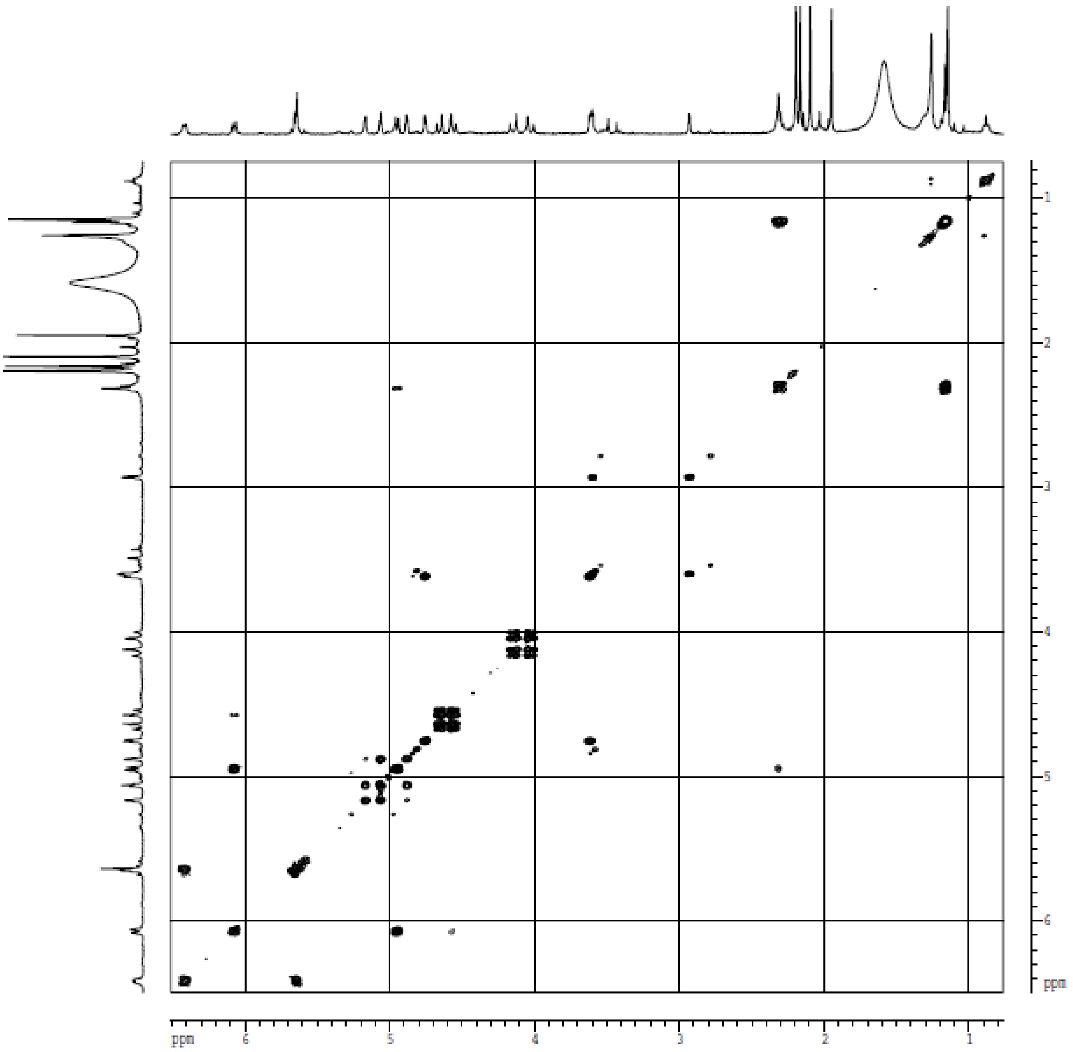

S15. HMBC spectrum of the new compound **2**.

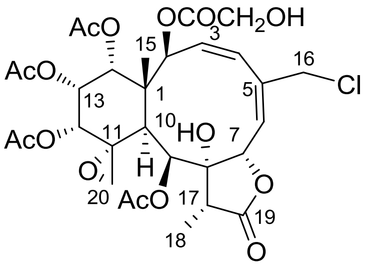

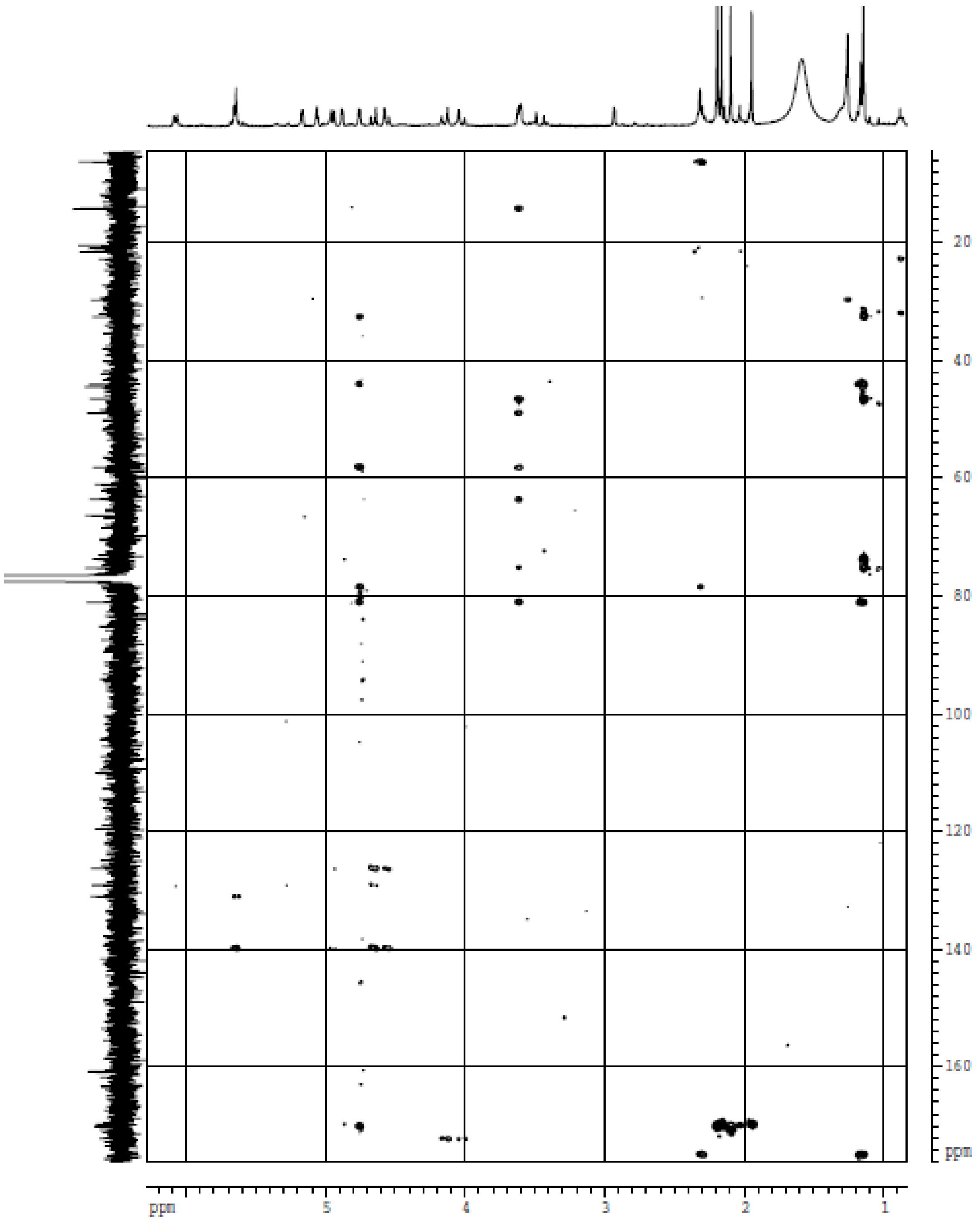

S16. NOESY spectrum of the new compound **2**.

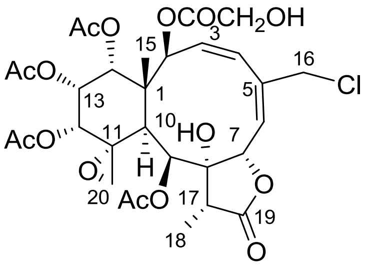

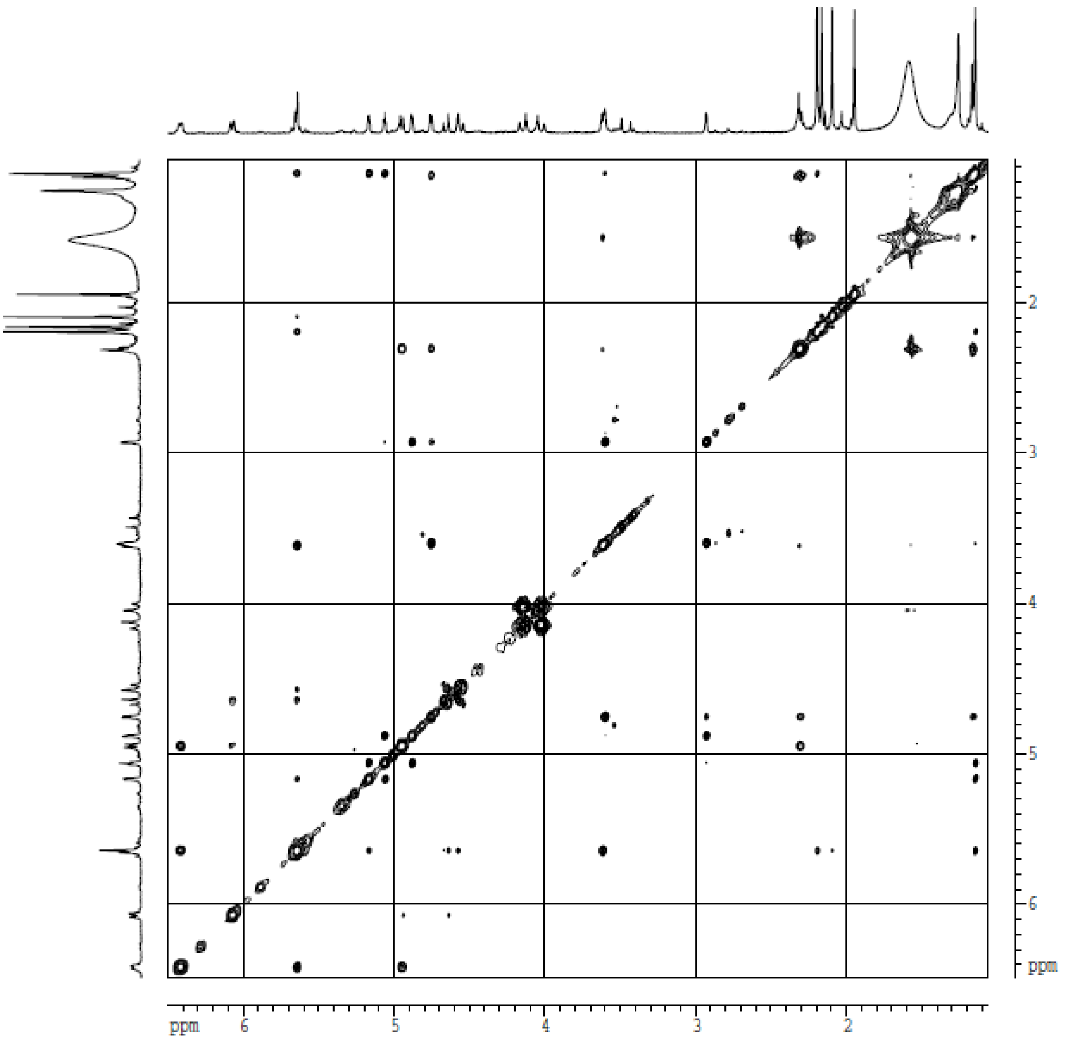

S17. HR-ESIMS spectrum of the new compound **3**.

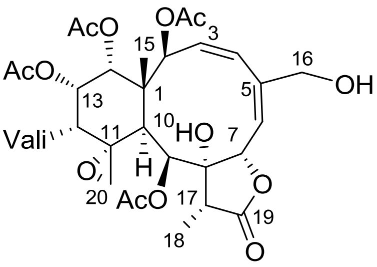

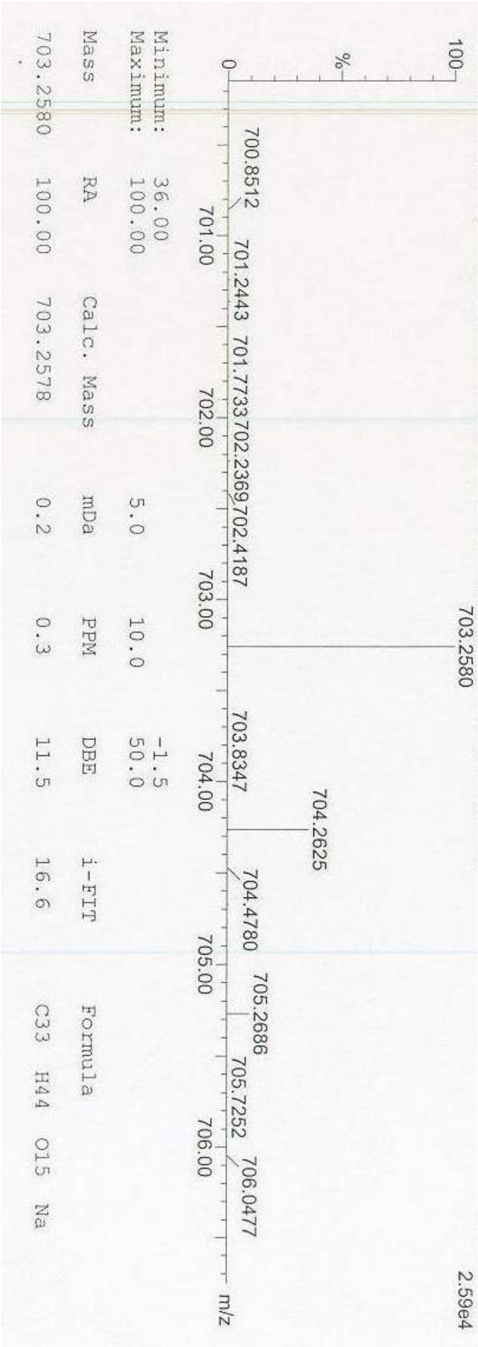

S18. ^{1}H NMR spectrum of the new compound **3**.

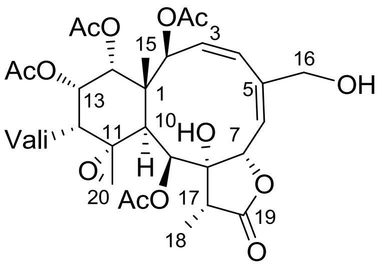

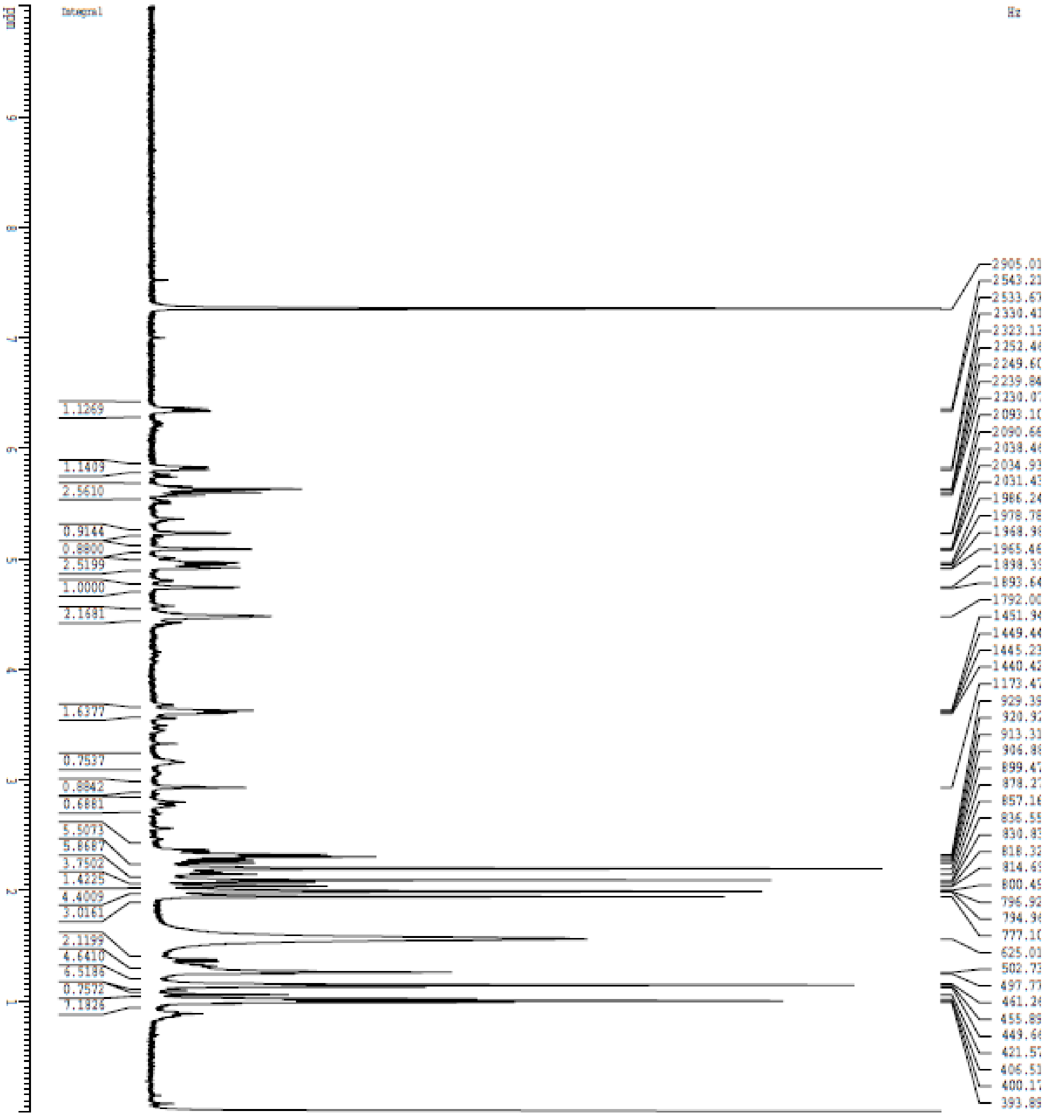

S19. ^{13}C NMR spectrum of the new compound **3**.

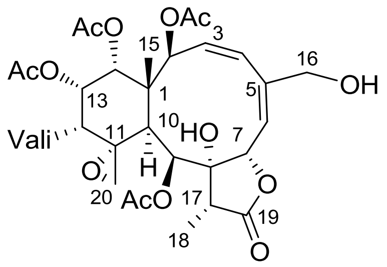

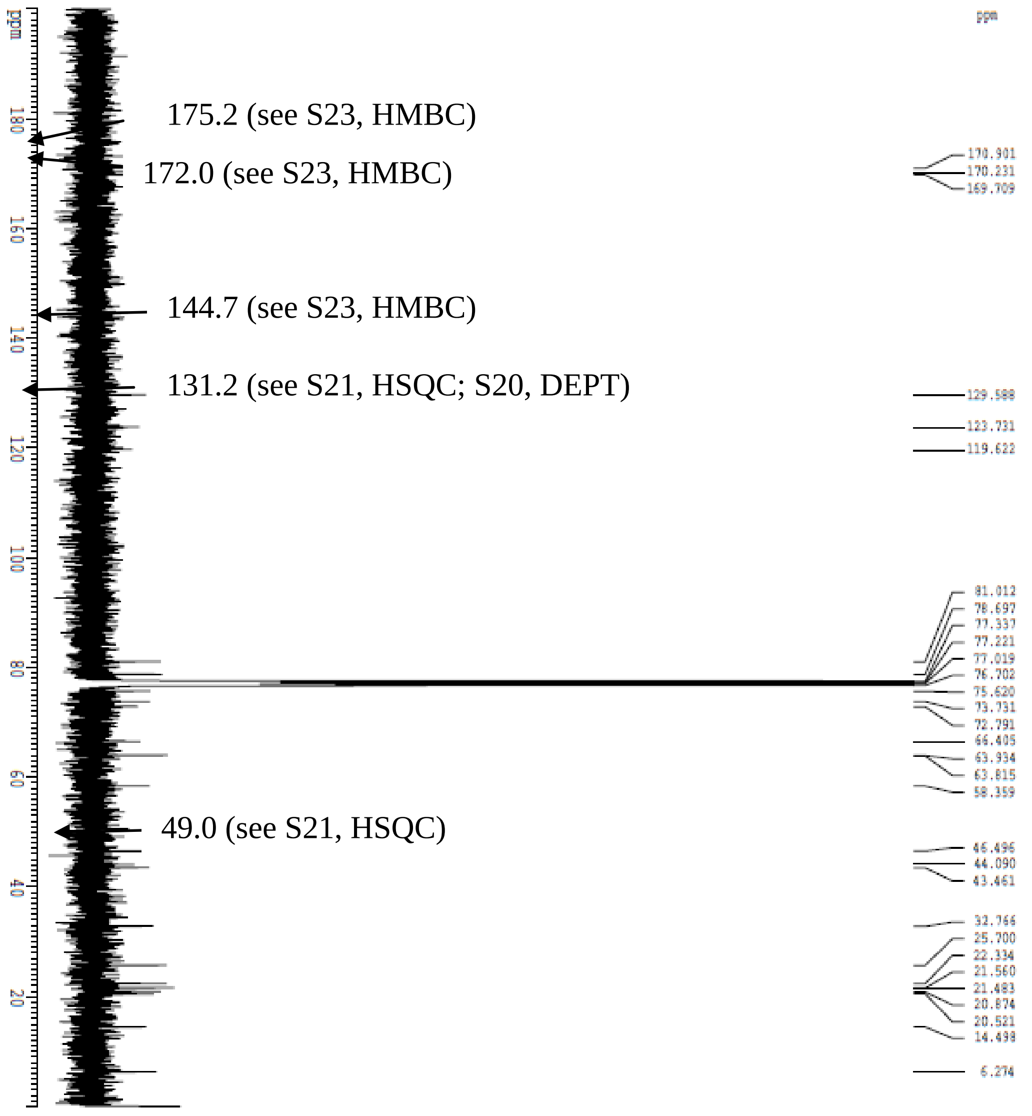

S20. DEPT spectrum of the new compound **3**.

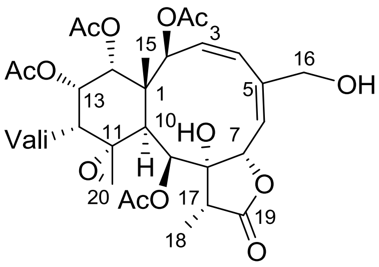

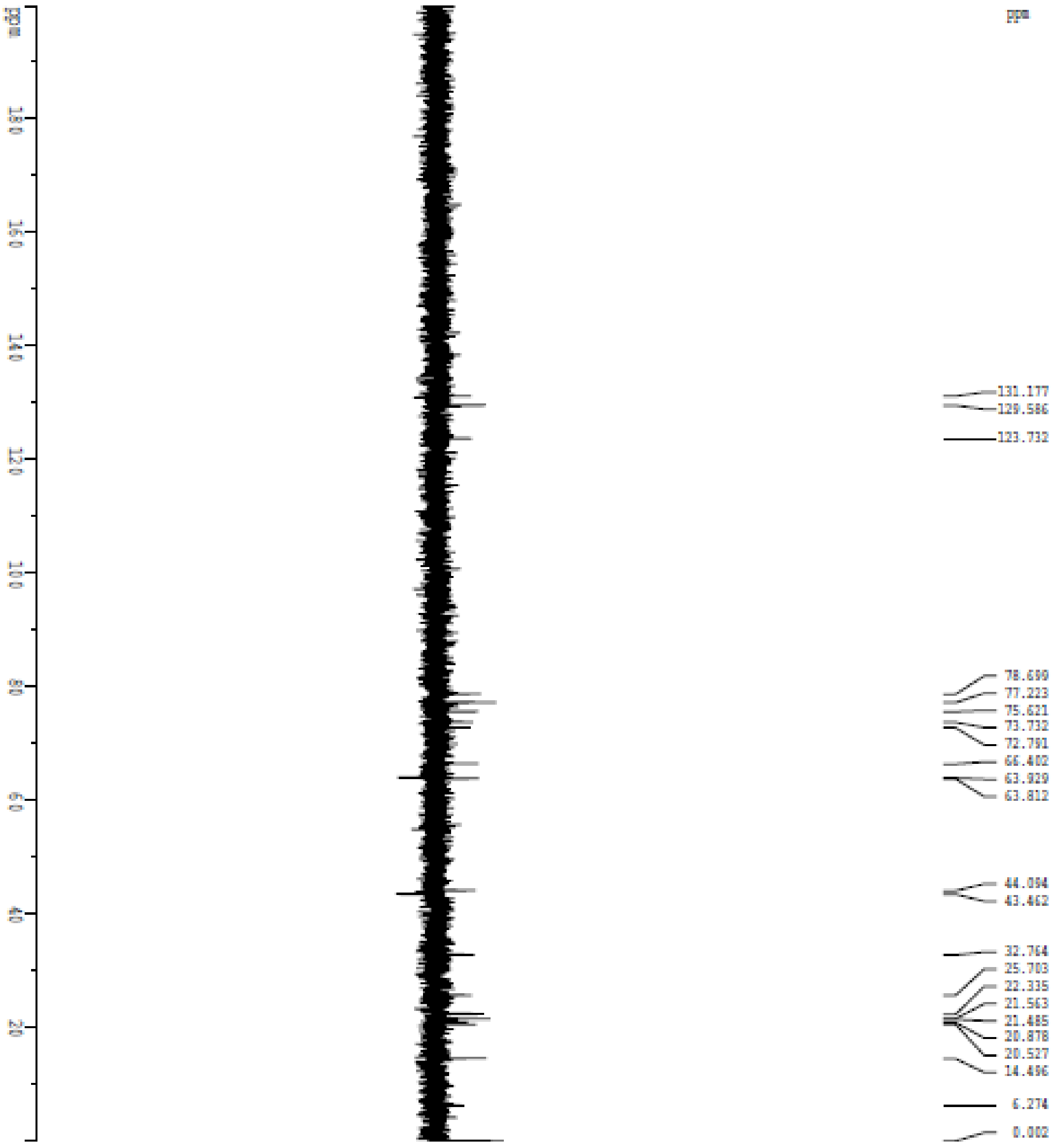

S21. HSQC spectrum of the new compound **3**.

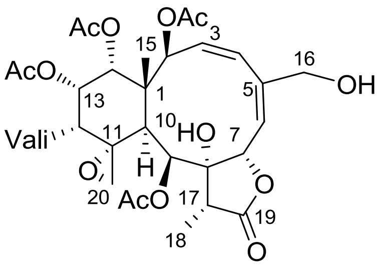

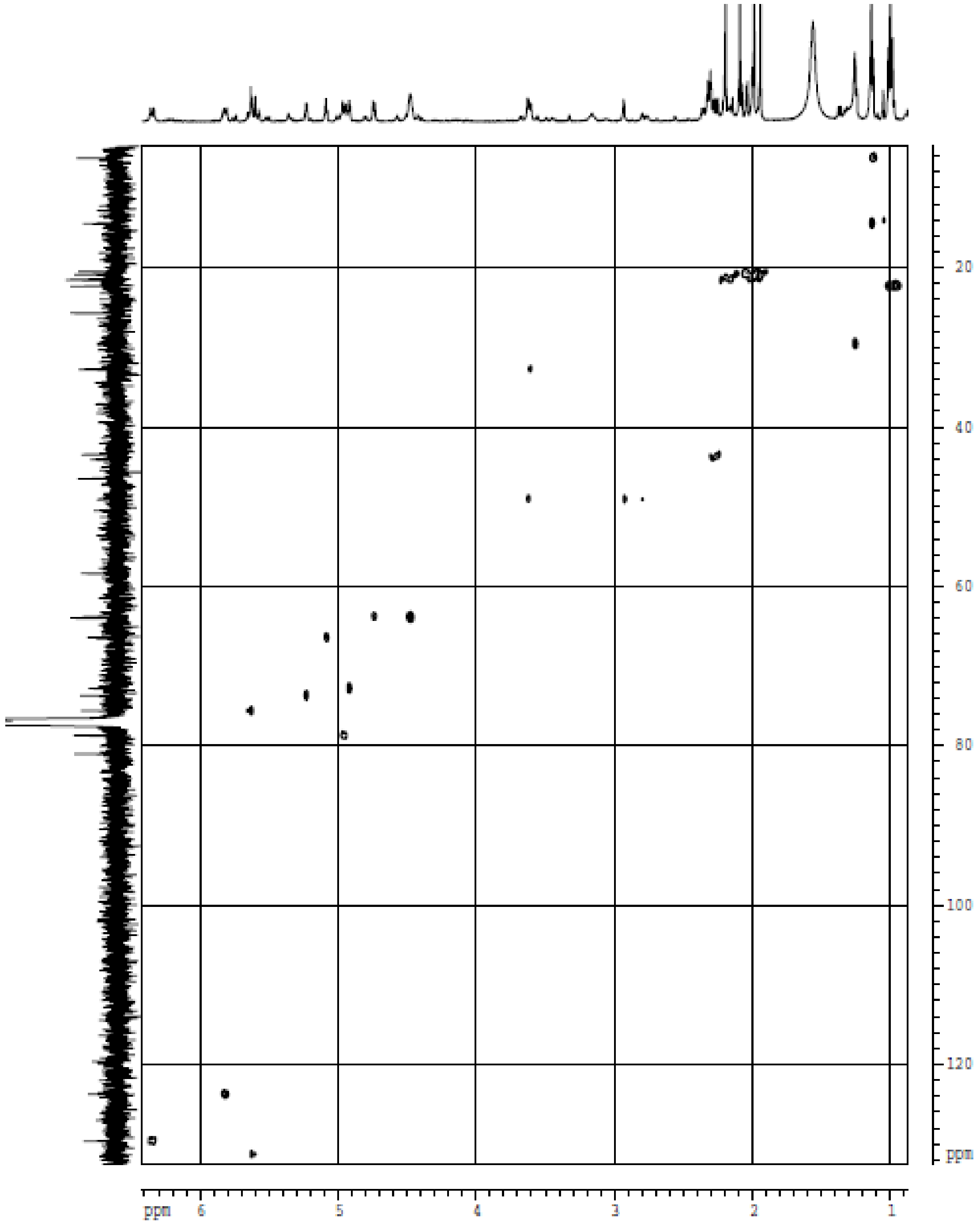

S22. ^1H-^1H COSY spectrum of the new compound **3**.

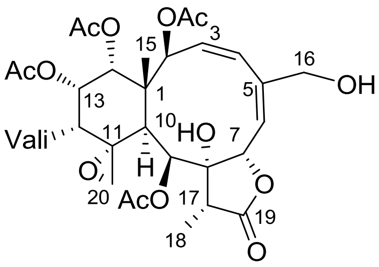

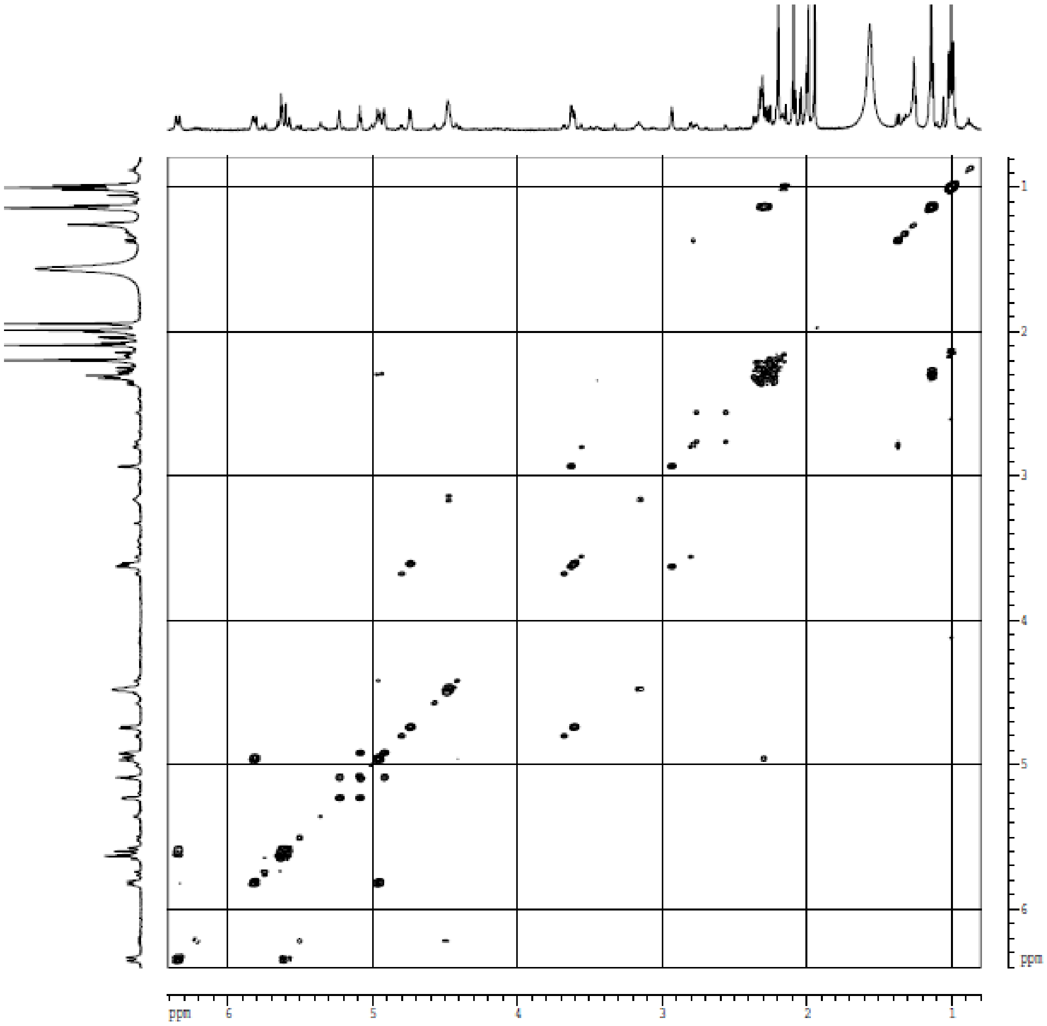

S23. HMBC spectrum of the new compound **3**.

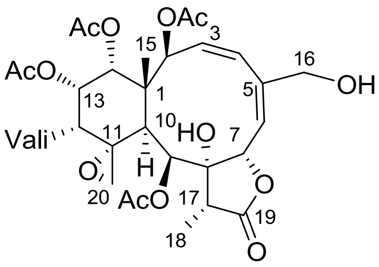

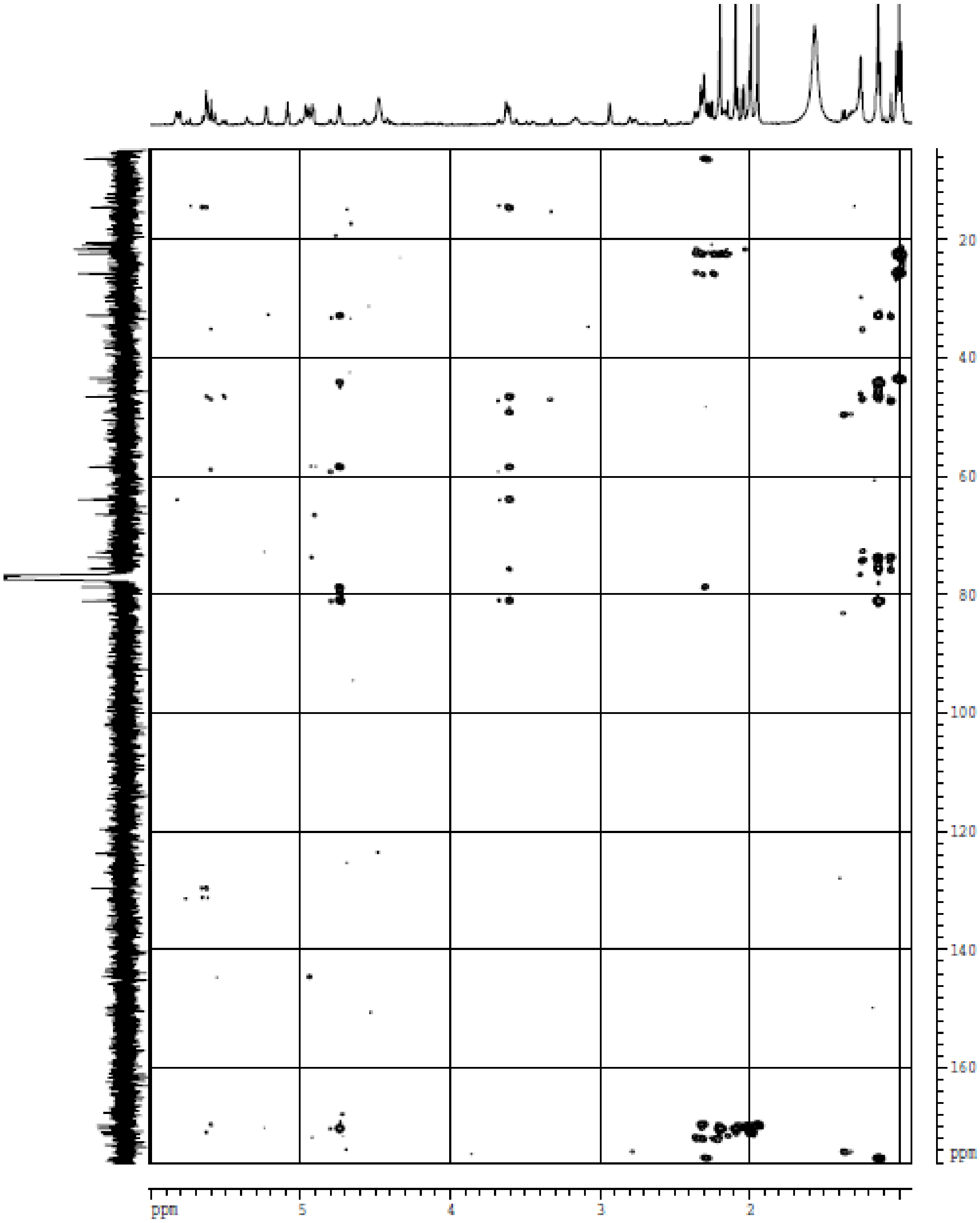

S24. NOESY spectrum of the new compound **3**.

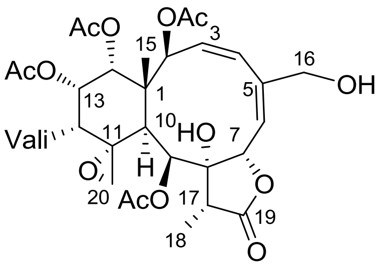

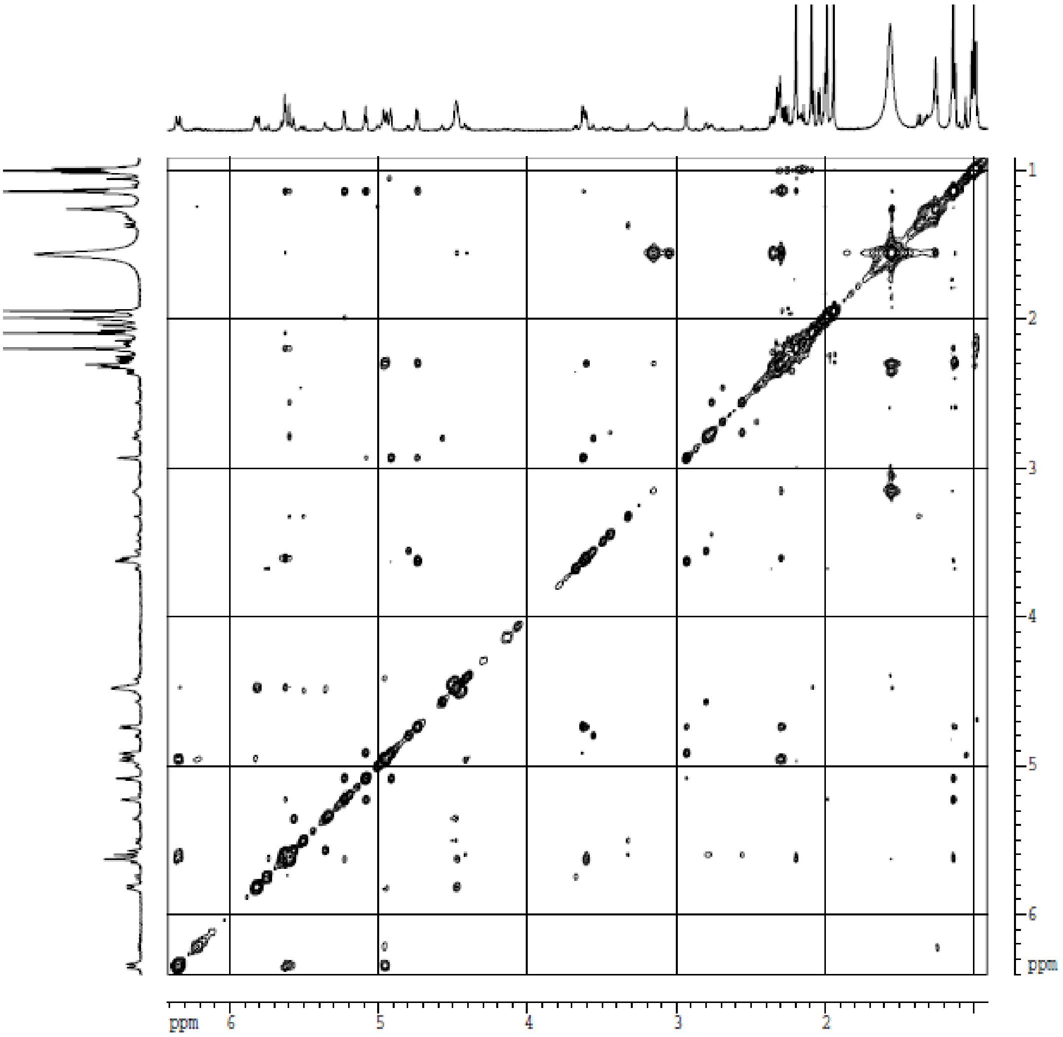

S25. HR-ESIMS spectrum of the new compound **4**.

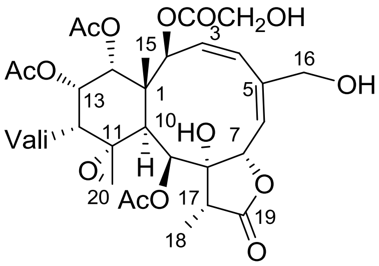

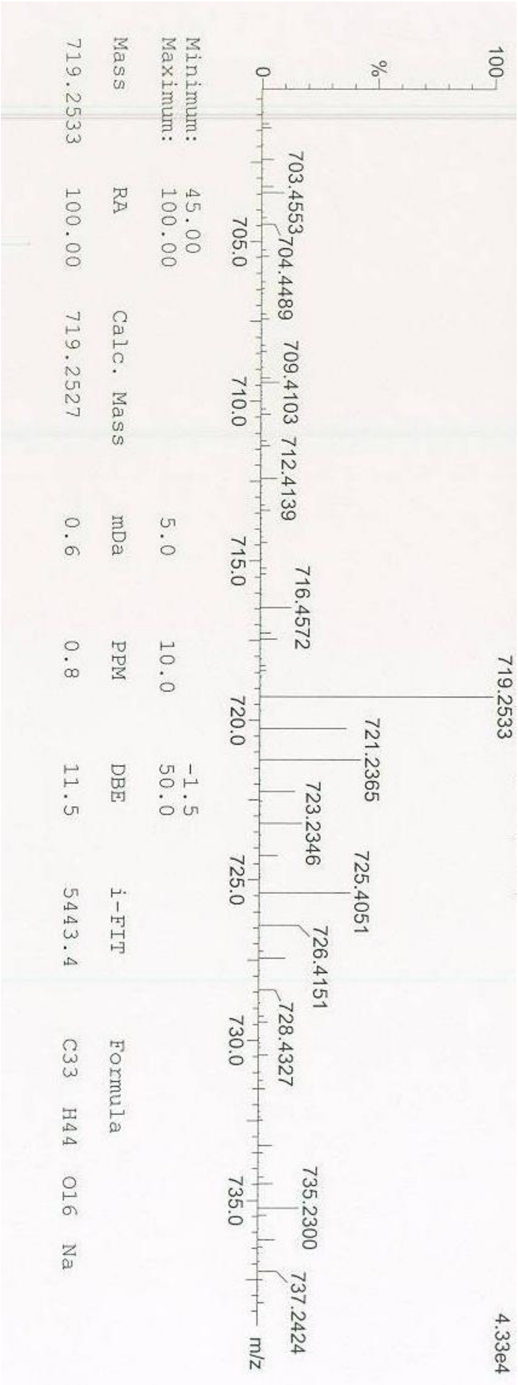

S26. ^{1}H NMR spectrum of the new compound **4**.

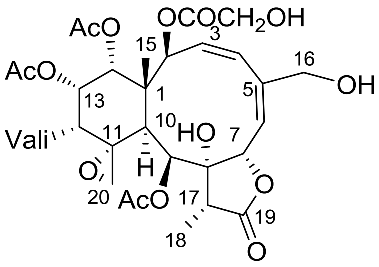

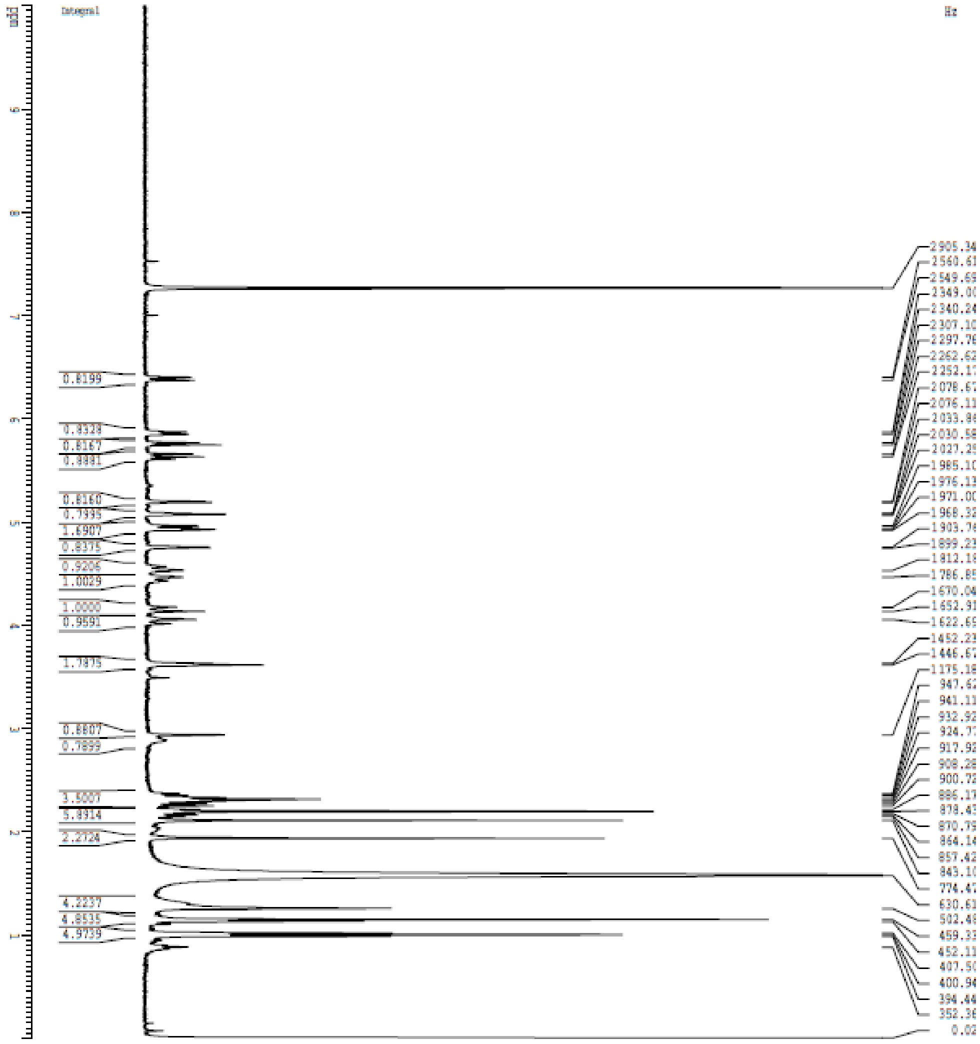

S27. ^{13}C NMR spectrum of the new compound **4**.

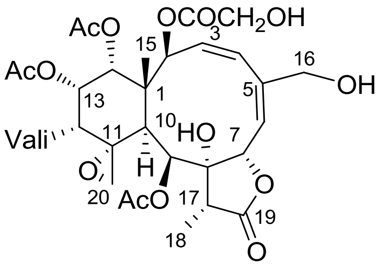

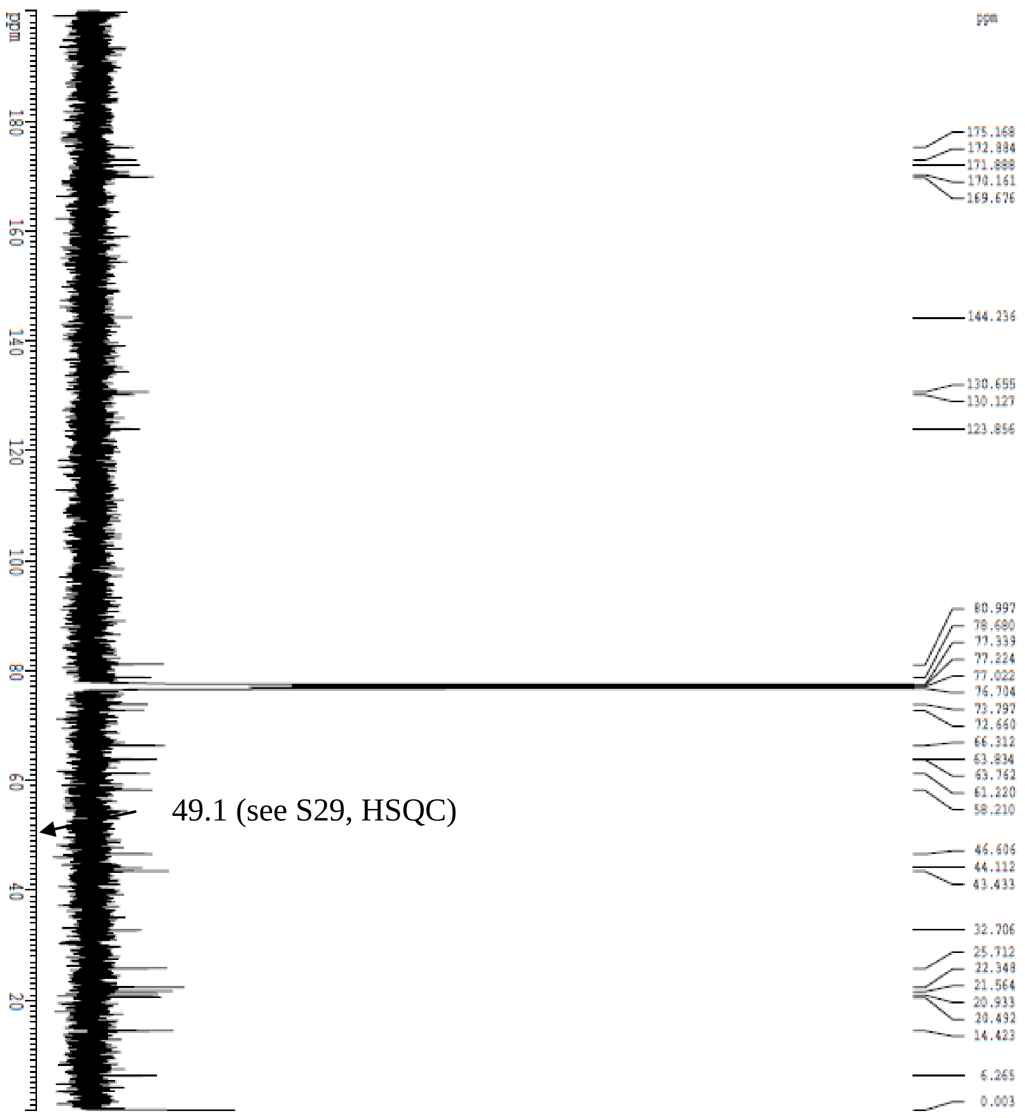

S28. DEPT spectrum of the new compound **4**.

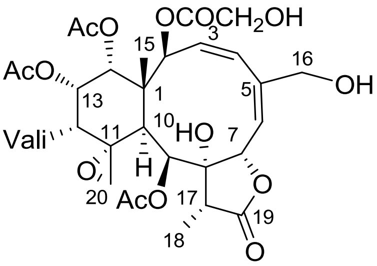

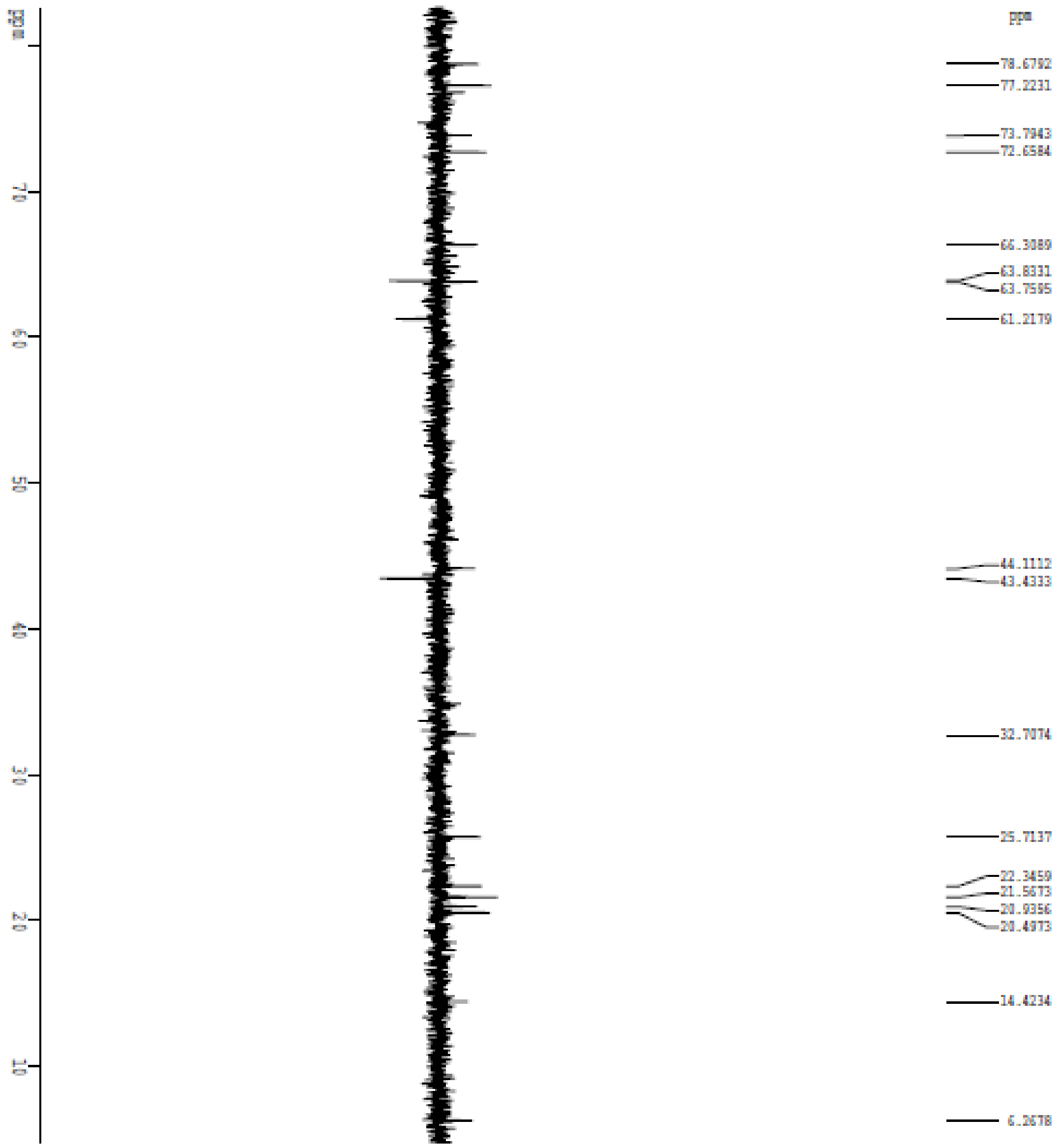

S29. HSQC spectrum of the new compound **4**.

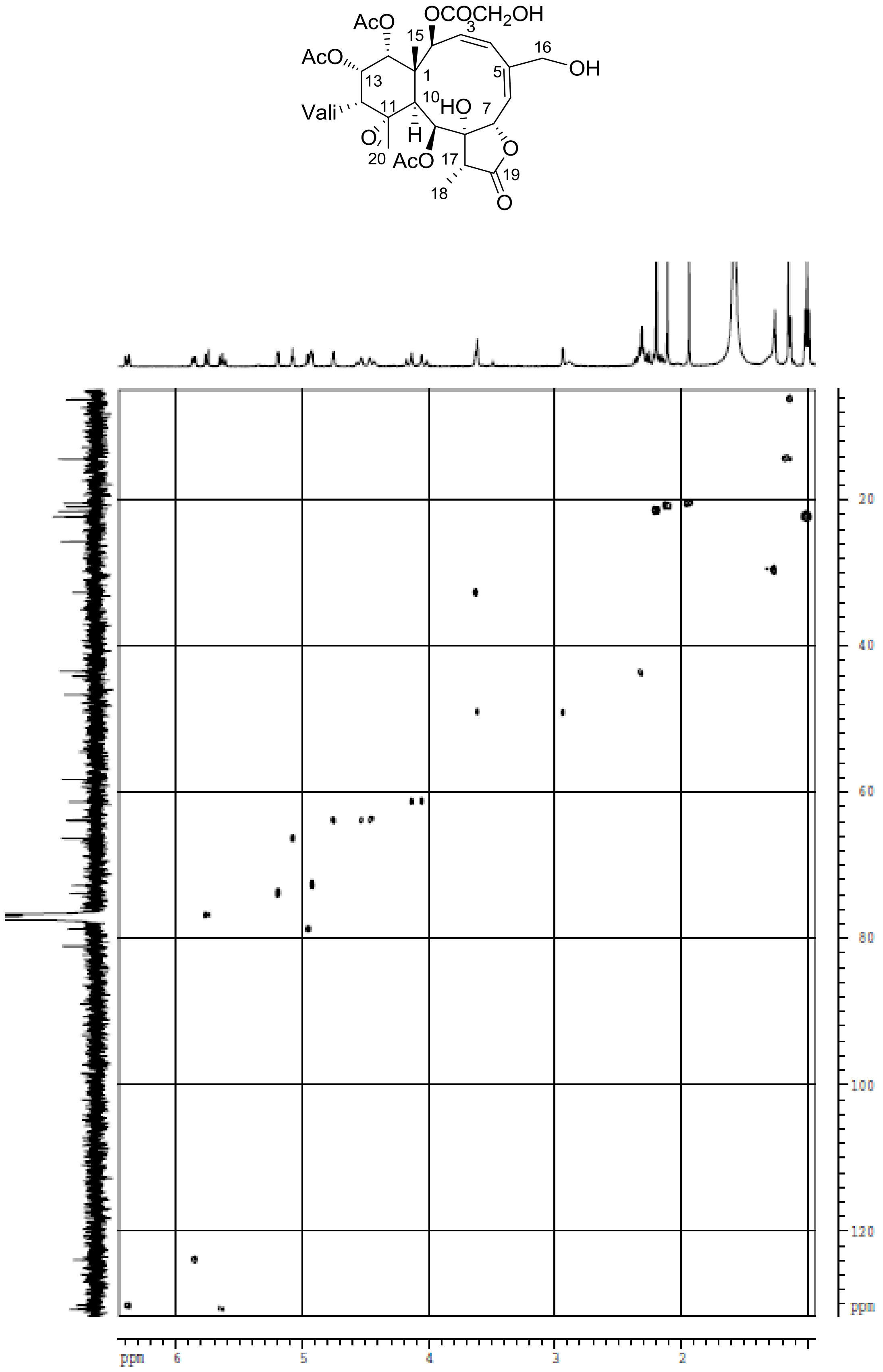

S30. ¹H-¹H COSY spectrum of the new compound **4**.

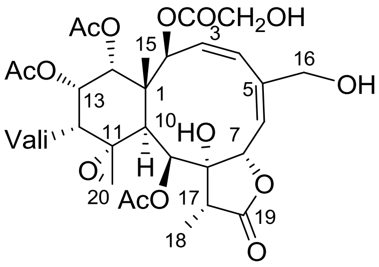

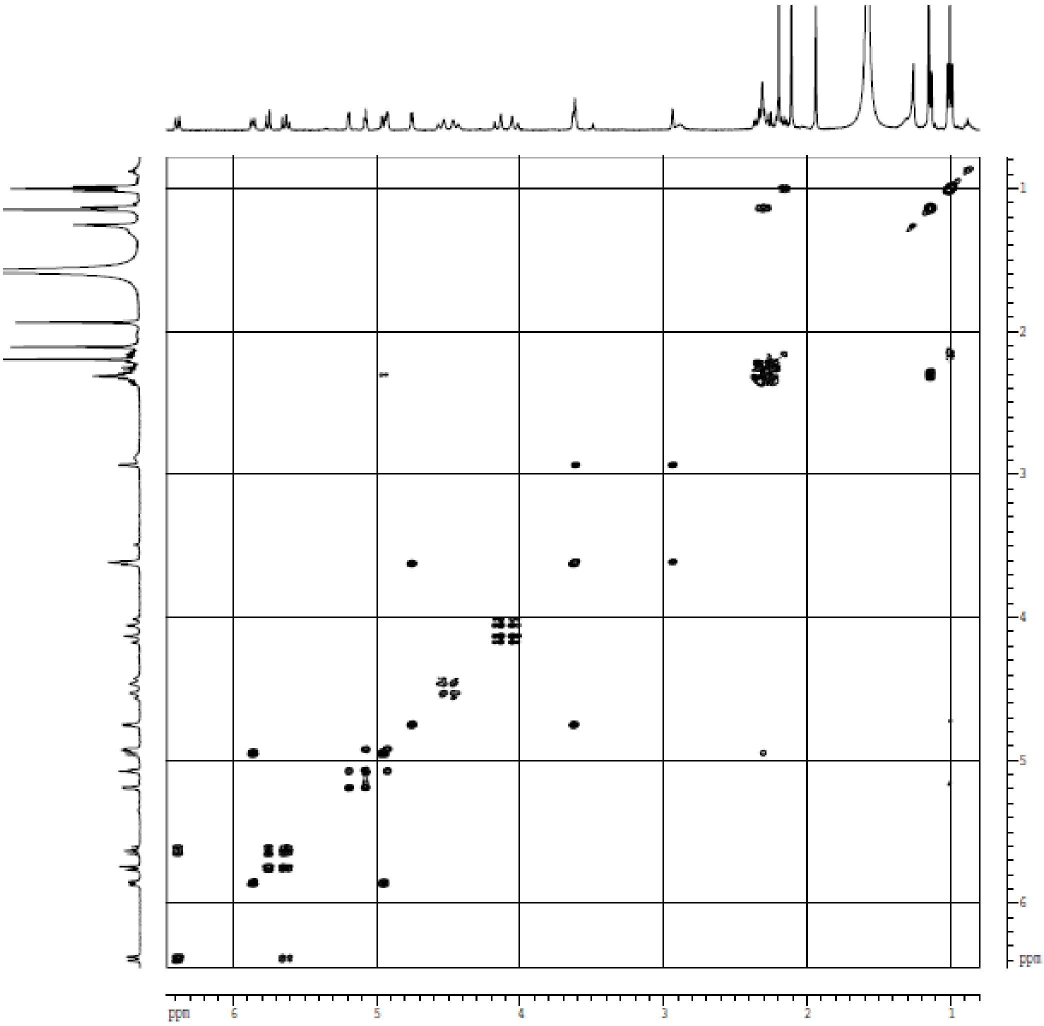

S31. HMBC spectrum of the new compound **4**.

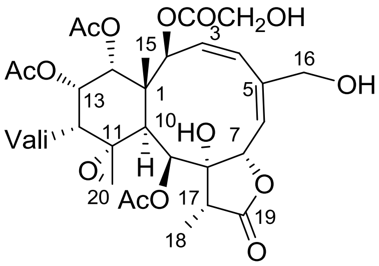

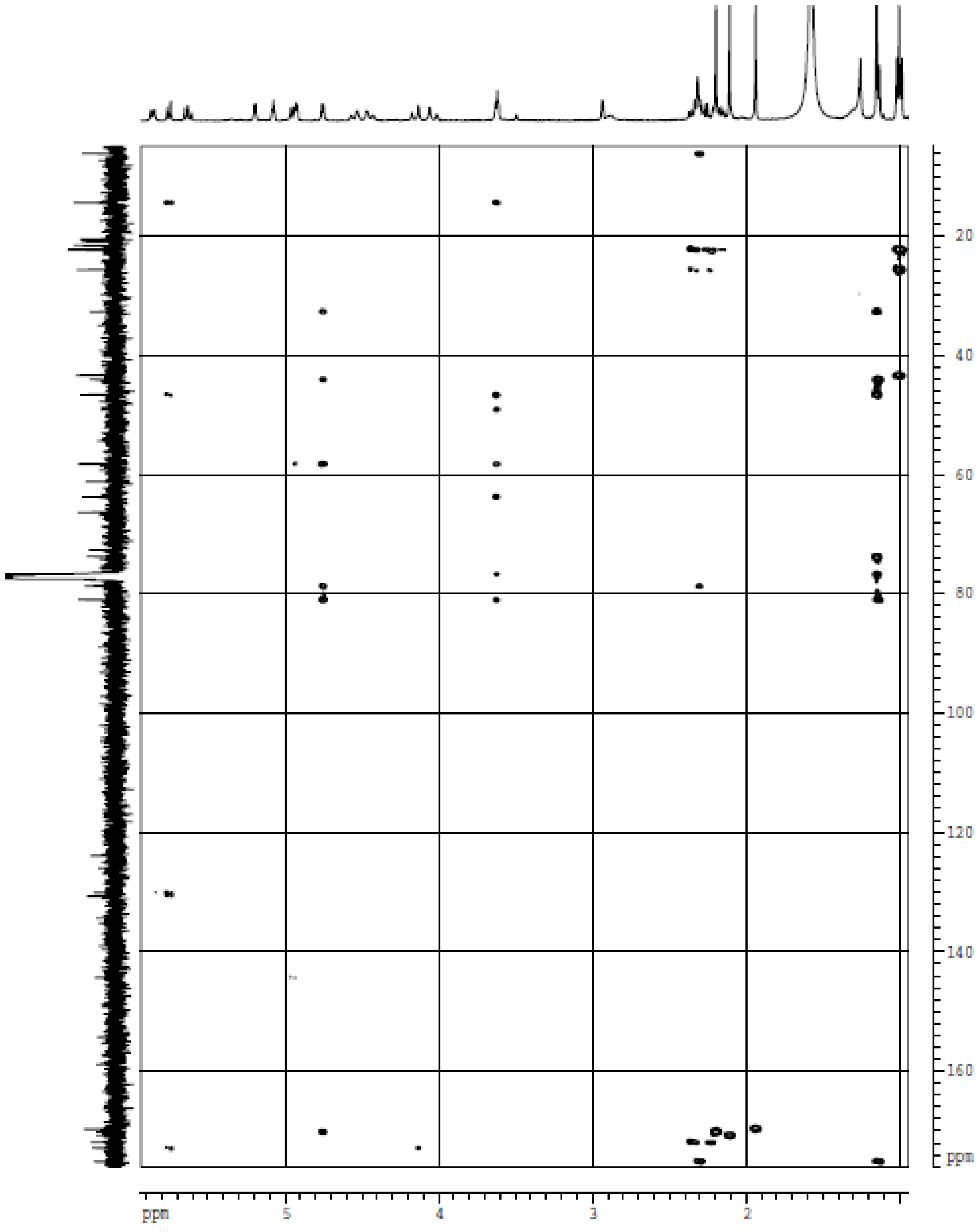

S32. NOESY spectrum of the new compound **4**.

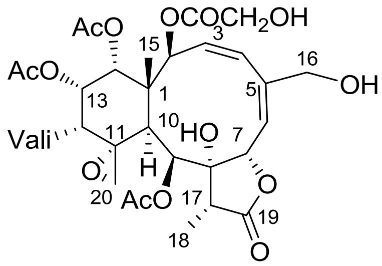

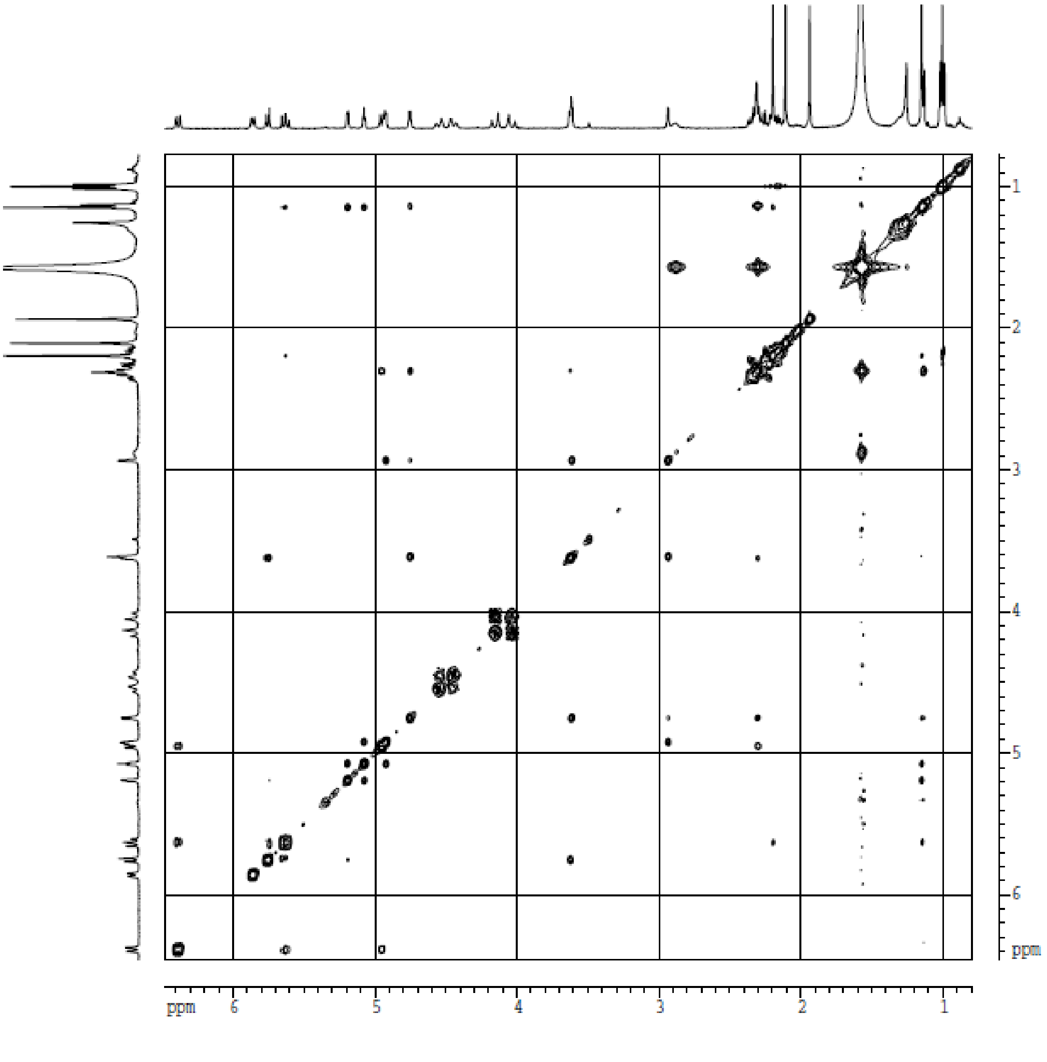

S33. HR-ESIMS spectrum of the new compound **5**.

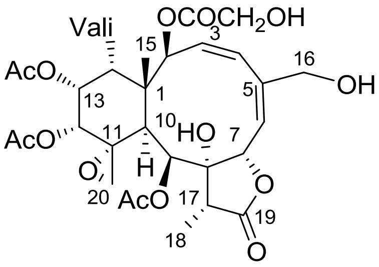

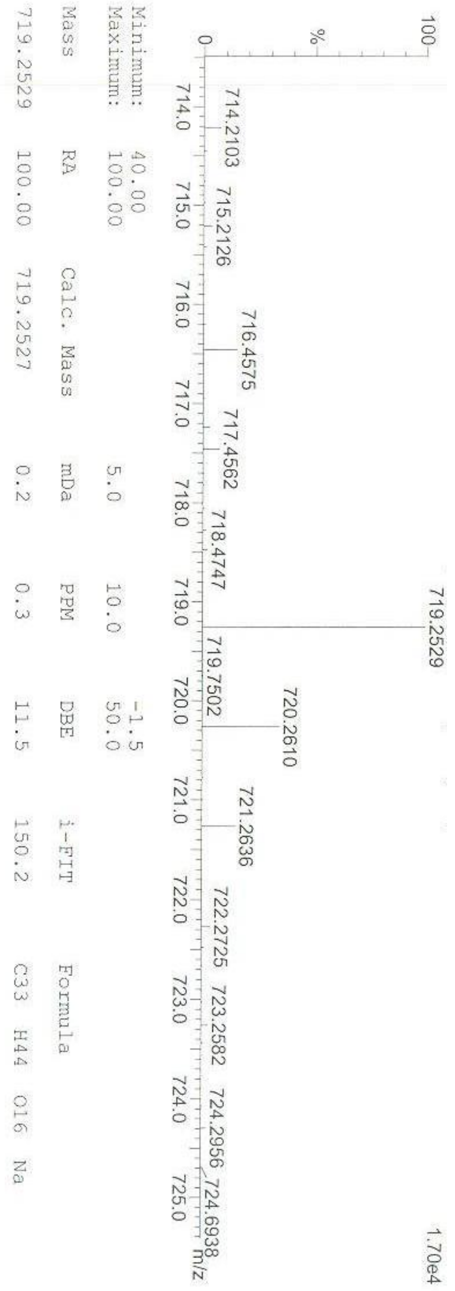

Minimum:	40.00				-1.5		
Maximum:	100.00		5.0	10.0	50.0		
Mass	RA	Calc. Mass	mDa	PPM	DBE	i-FIT	Formula
719.2529	100.00	719.2527	0.2	0.3	11.5	150.2	C33 H44 O16 Na

S34. ¹H NMR spectrum of the new compound **5**.

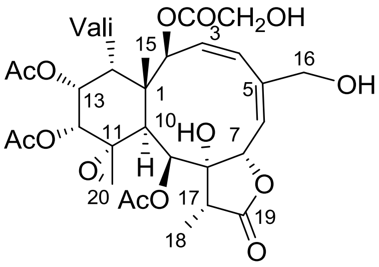

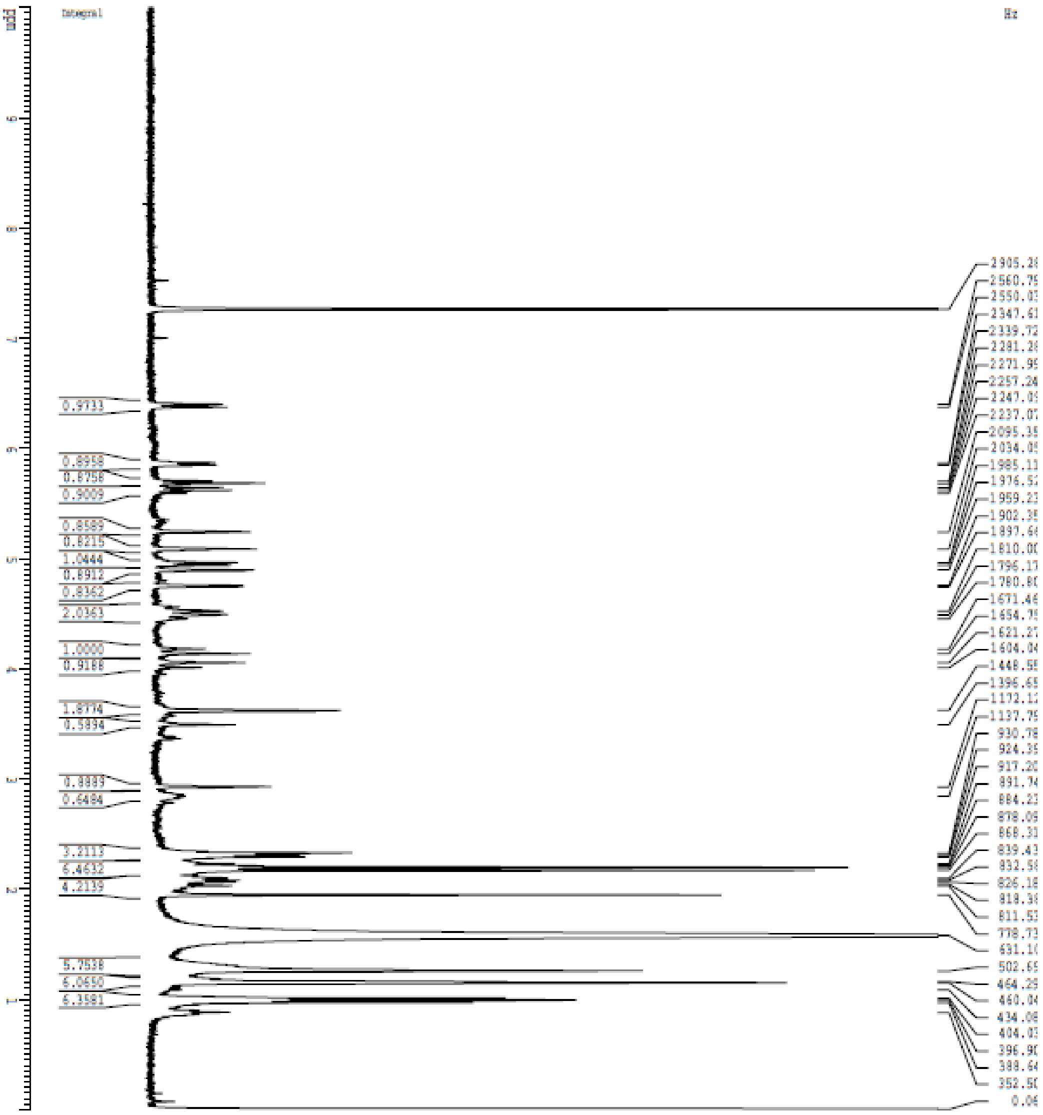

S35. ^{13}C NMR spectrum of the new compound **5**.

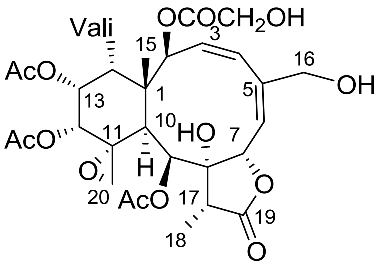

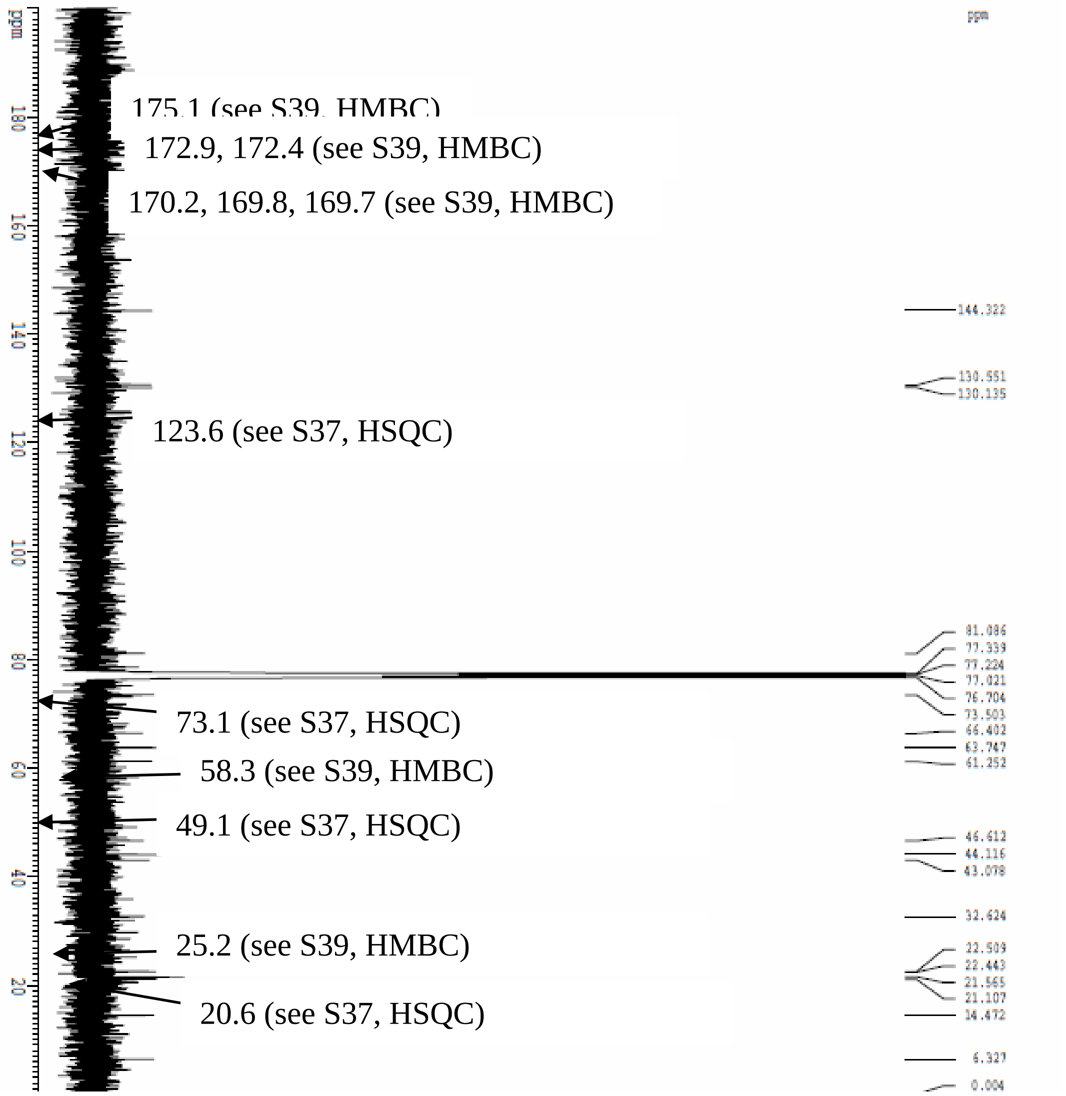

S36. DEPT spectrum of the new compound **5**.

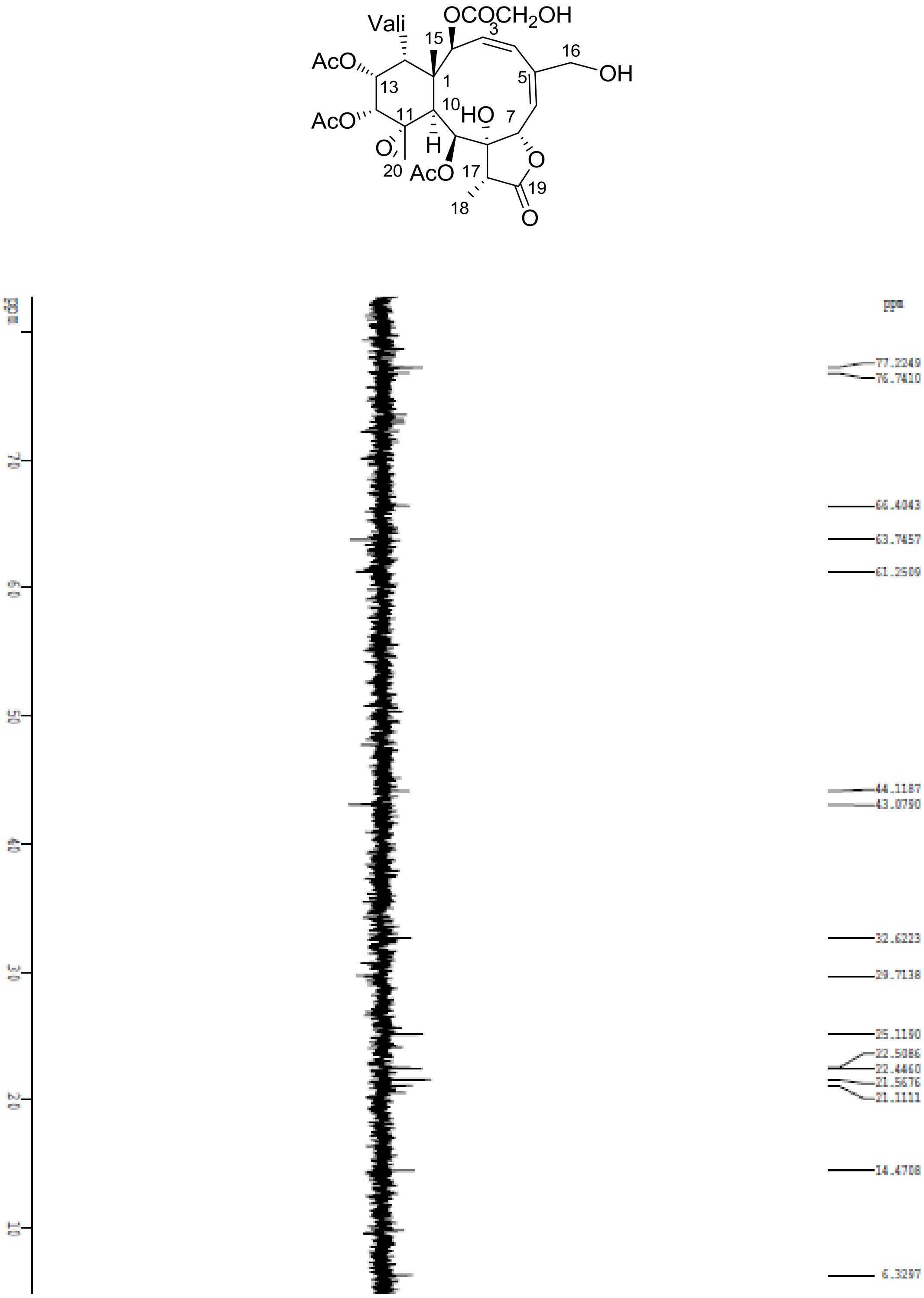

S37. HSQC spectrum of the new compound **5**.

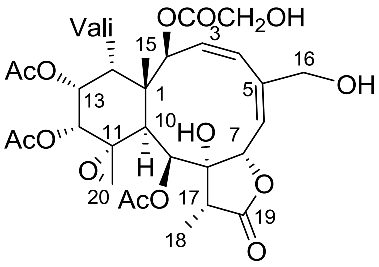

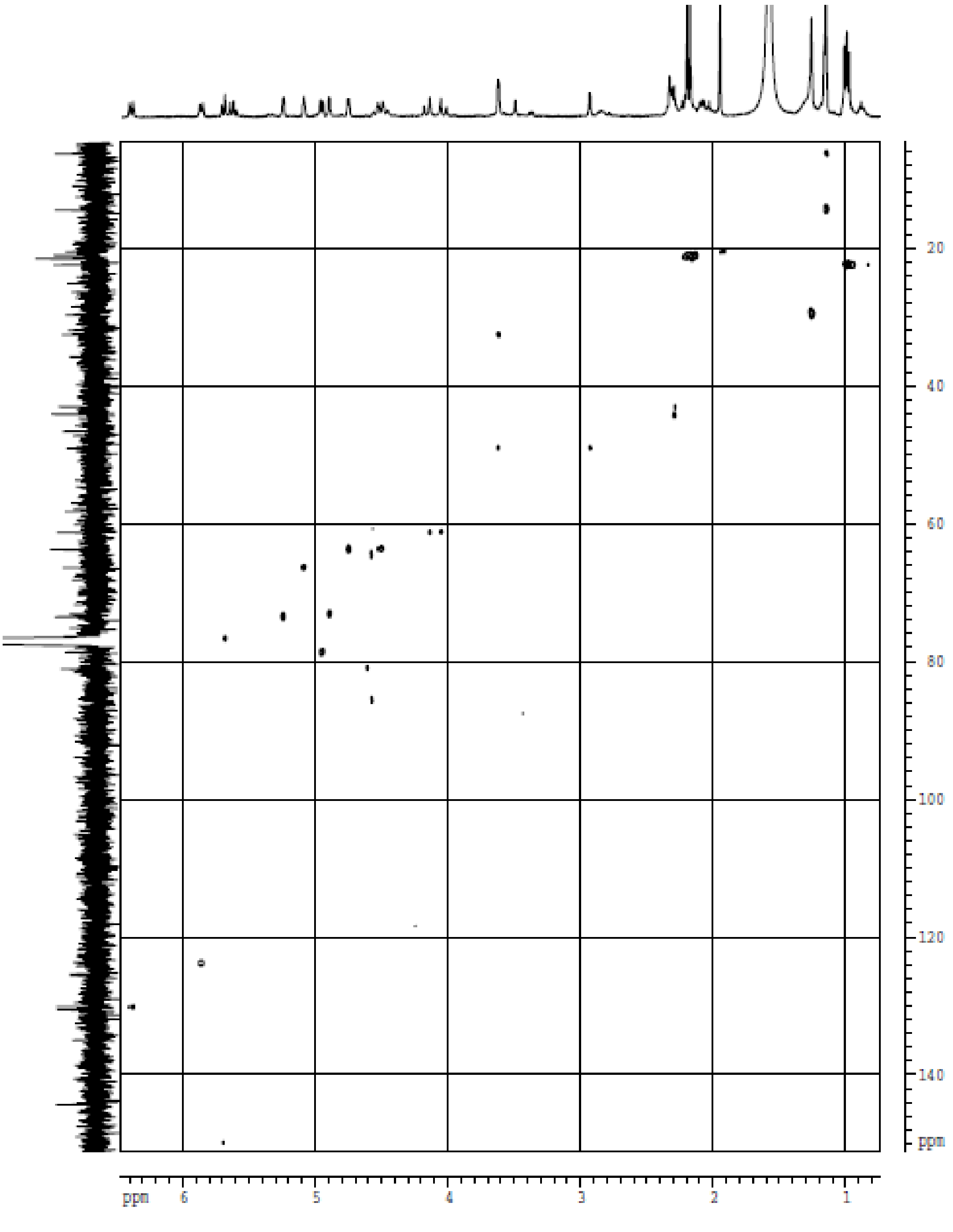

S38. ^{1}H-^{1}H COSY spectrum of the new compound **5**.

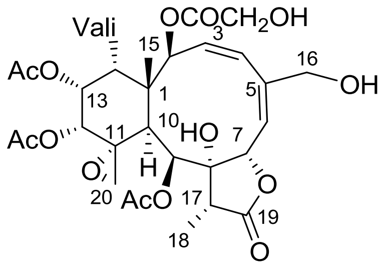

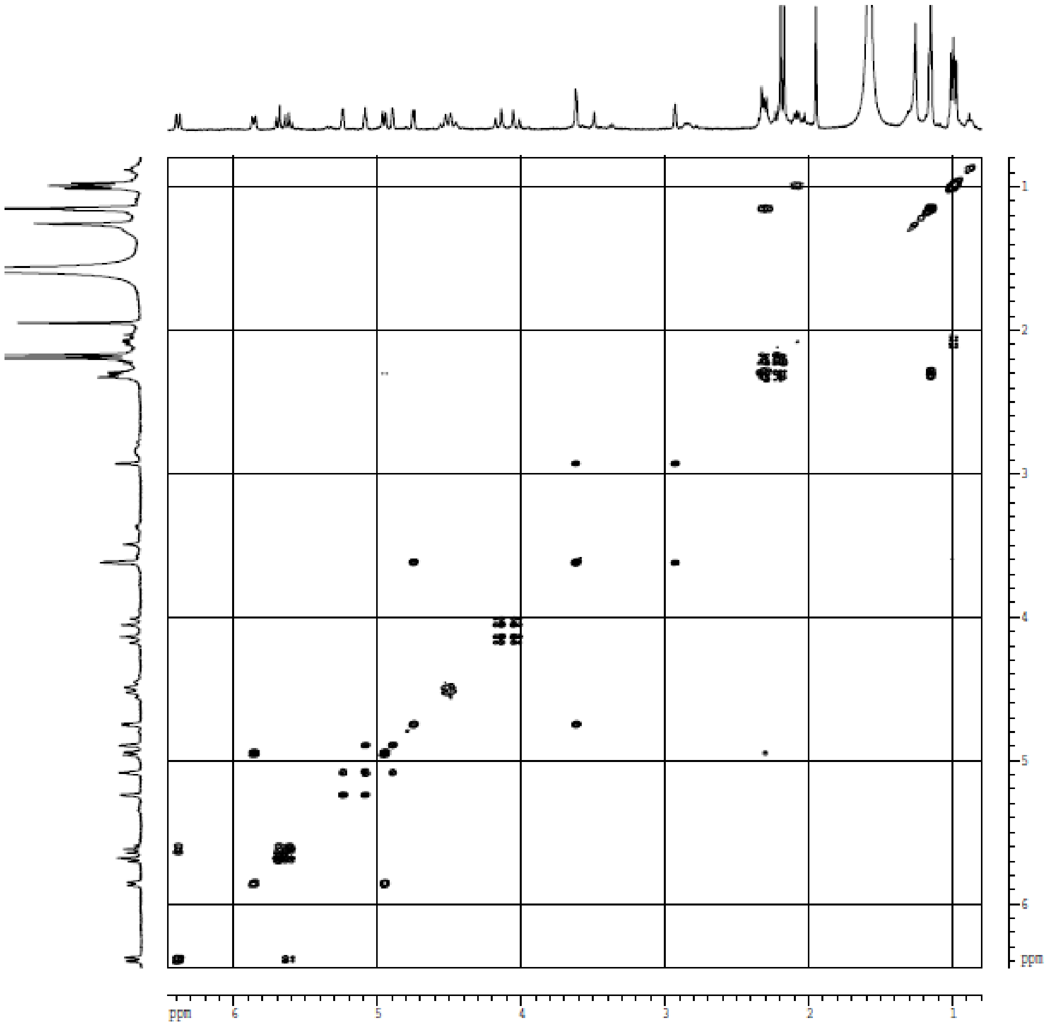

S39. HMBC spectrum of the new compound **5**.

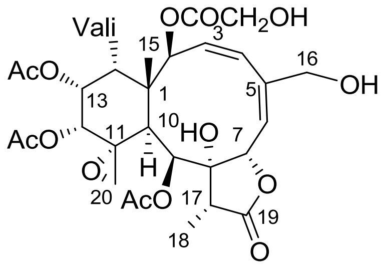

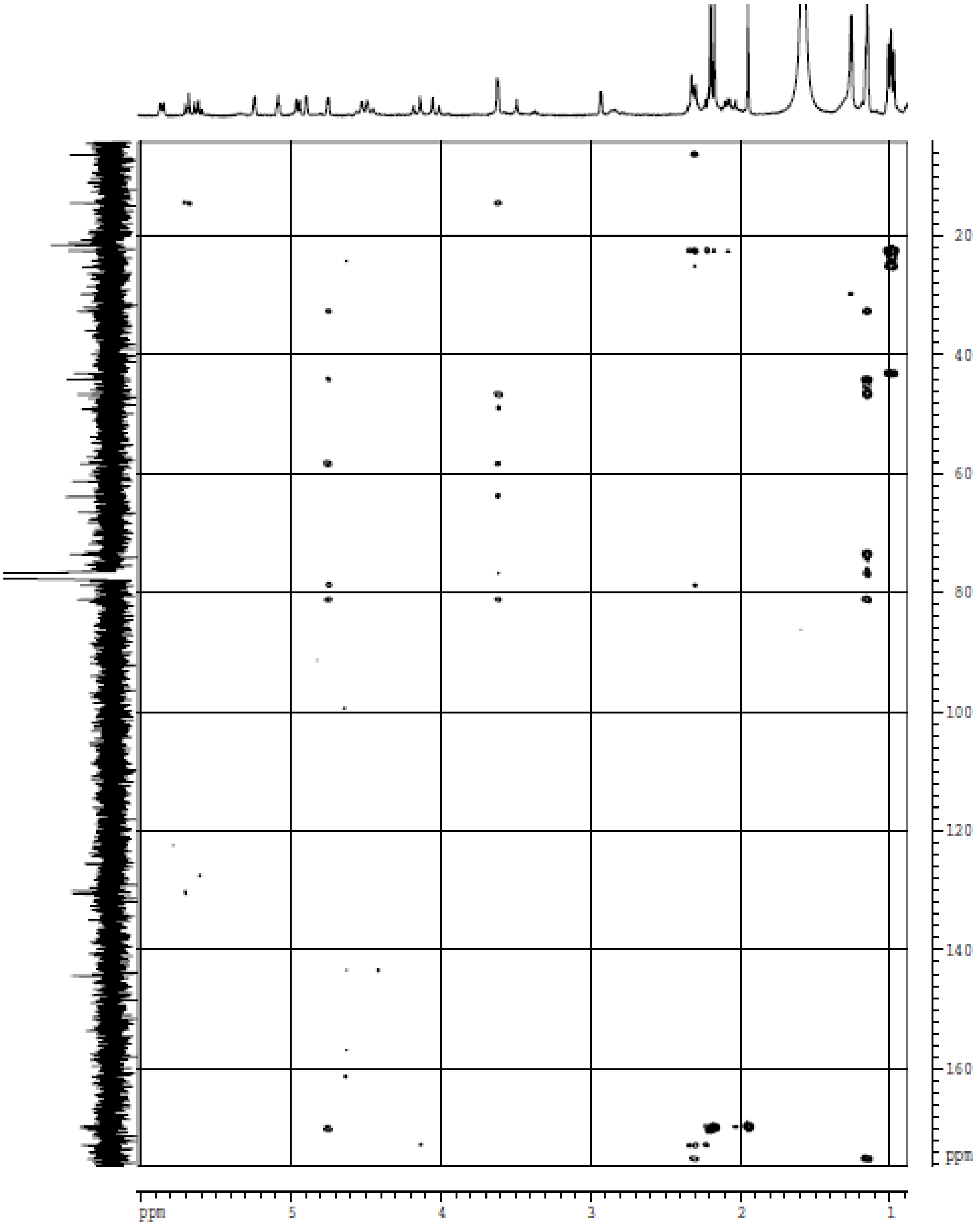

S40. NOESY spectrum of the new compound **5**.

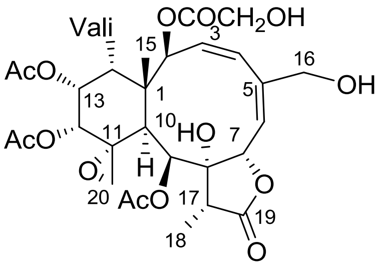

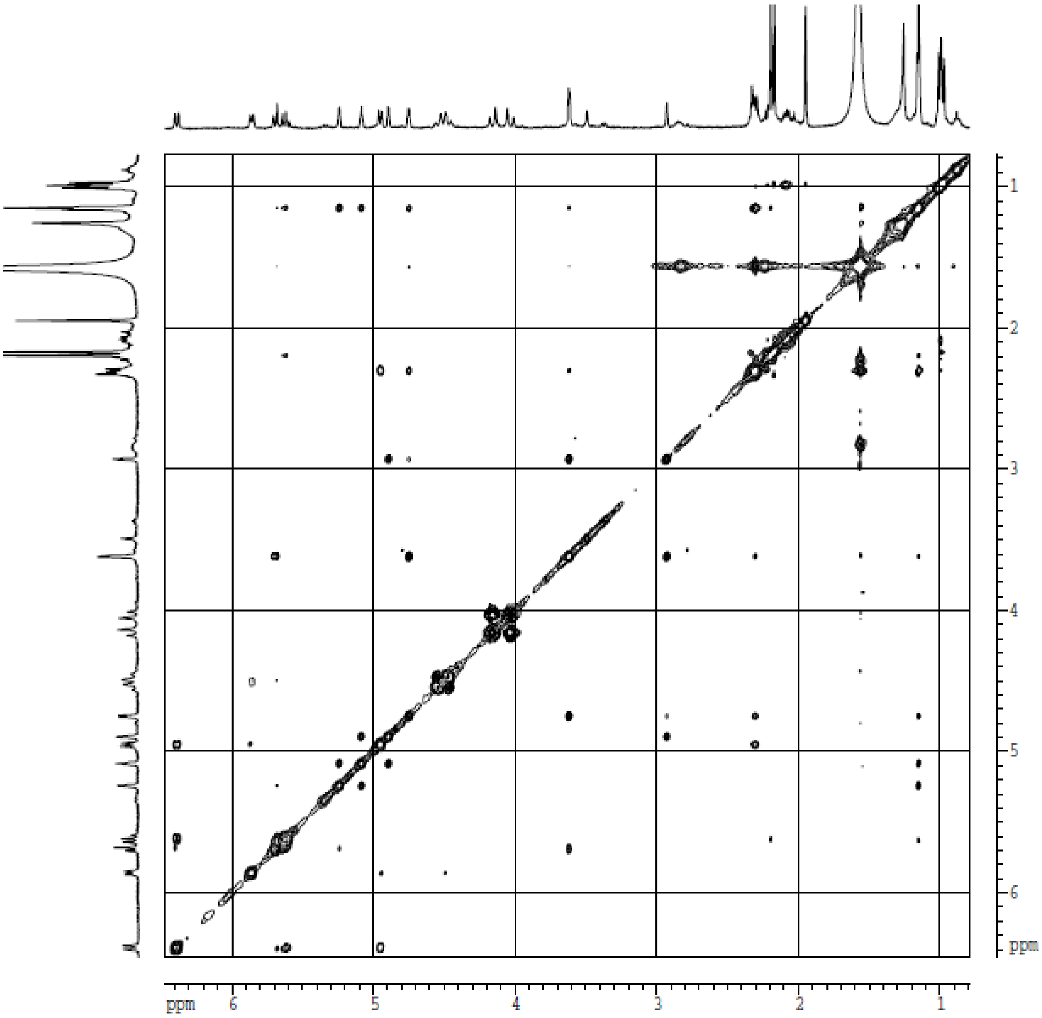

S41. HR-ESIMS spectrum of the new compound **6**.

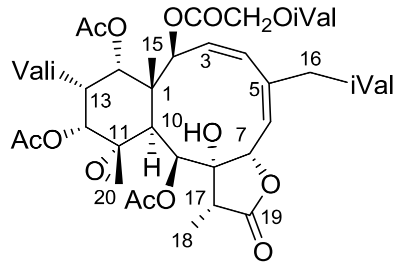

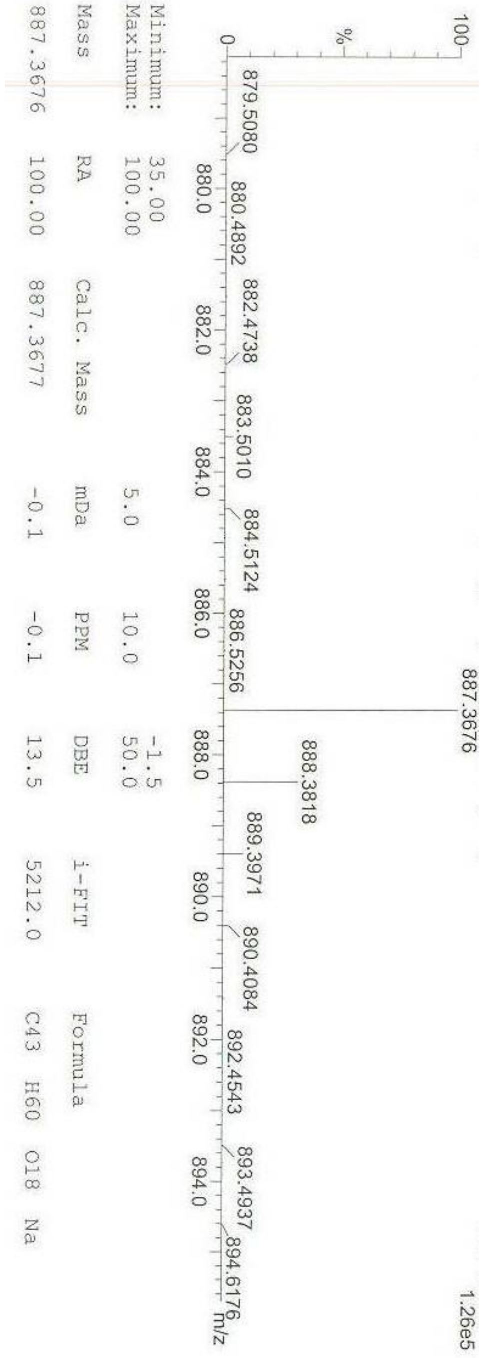

S42. ^{1}H NMR spectrum of the new compound **6**.

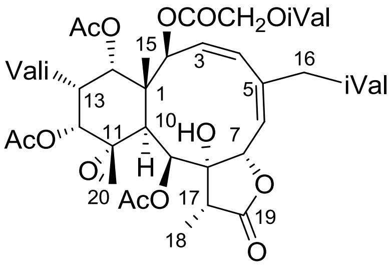

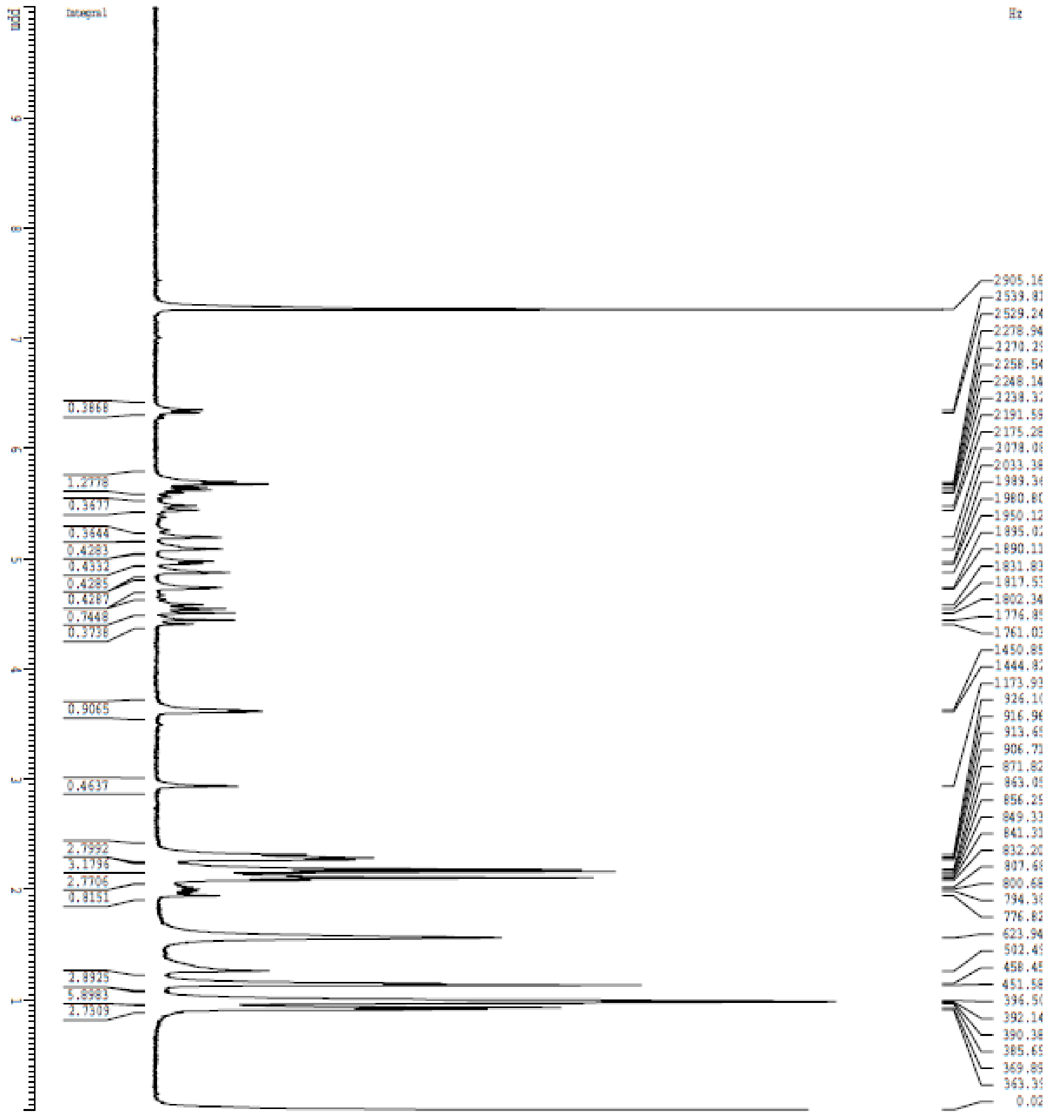

S43. ^{13}C NMR spectrum of the new compound **6**.

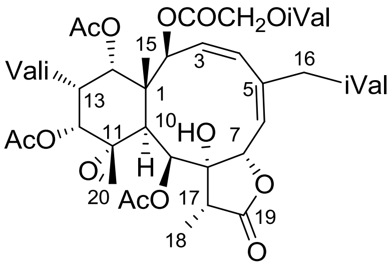

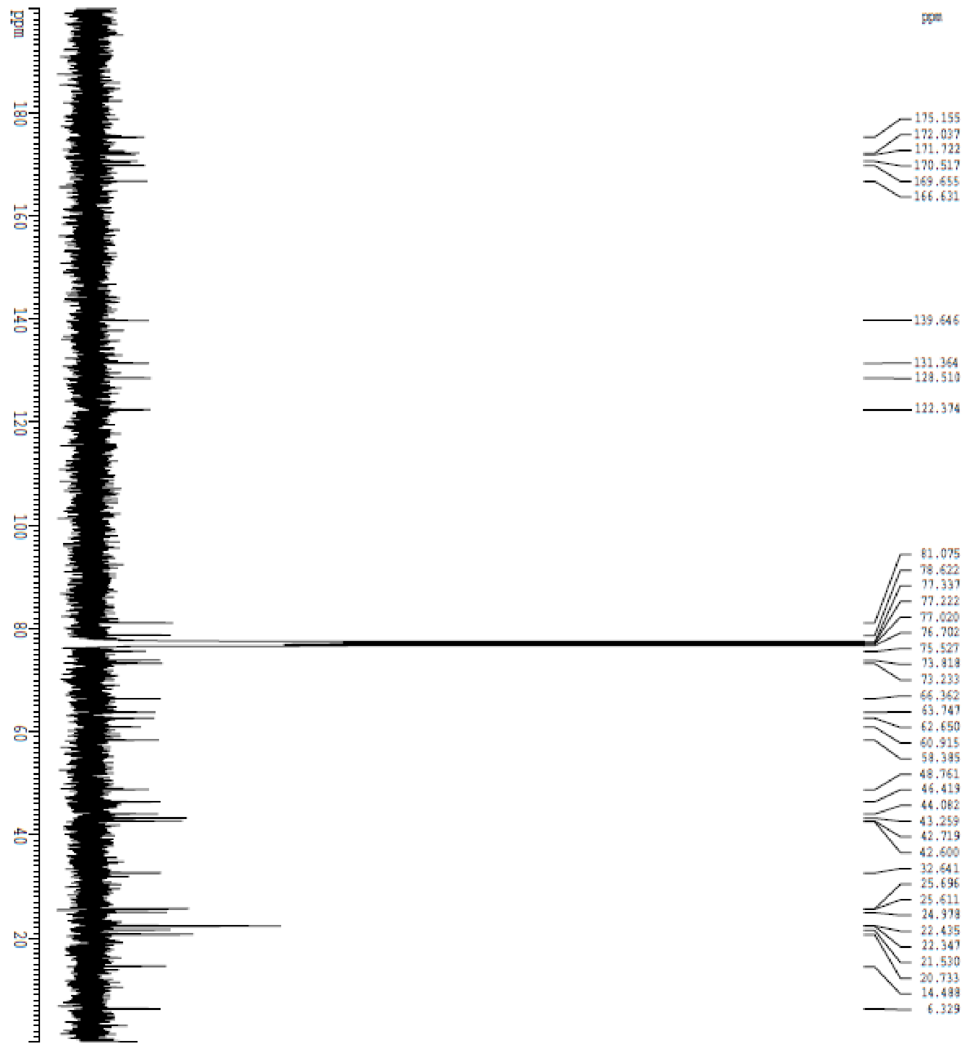

S44. [1] DEPT spectrum of the new compound **6**.

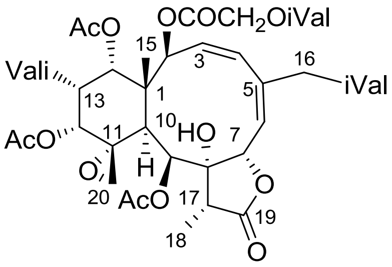

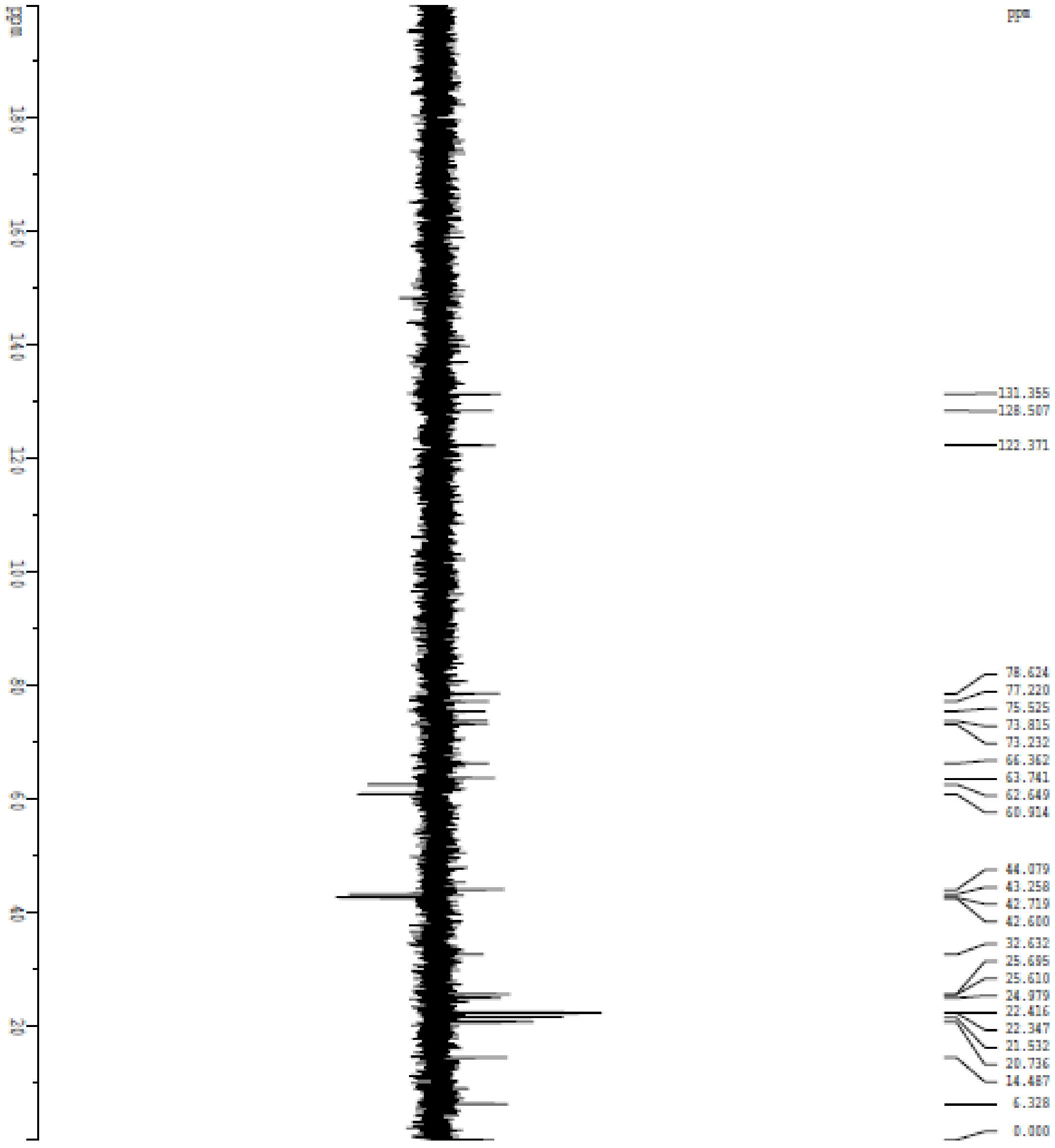

S45. HSQC spectrum of the new compound **6**.

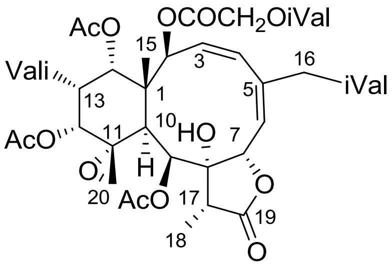

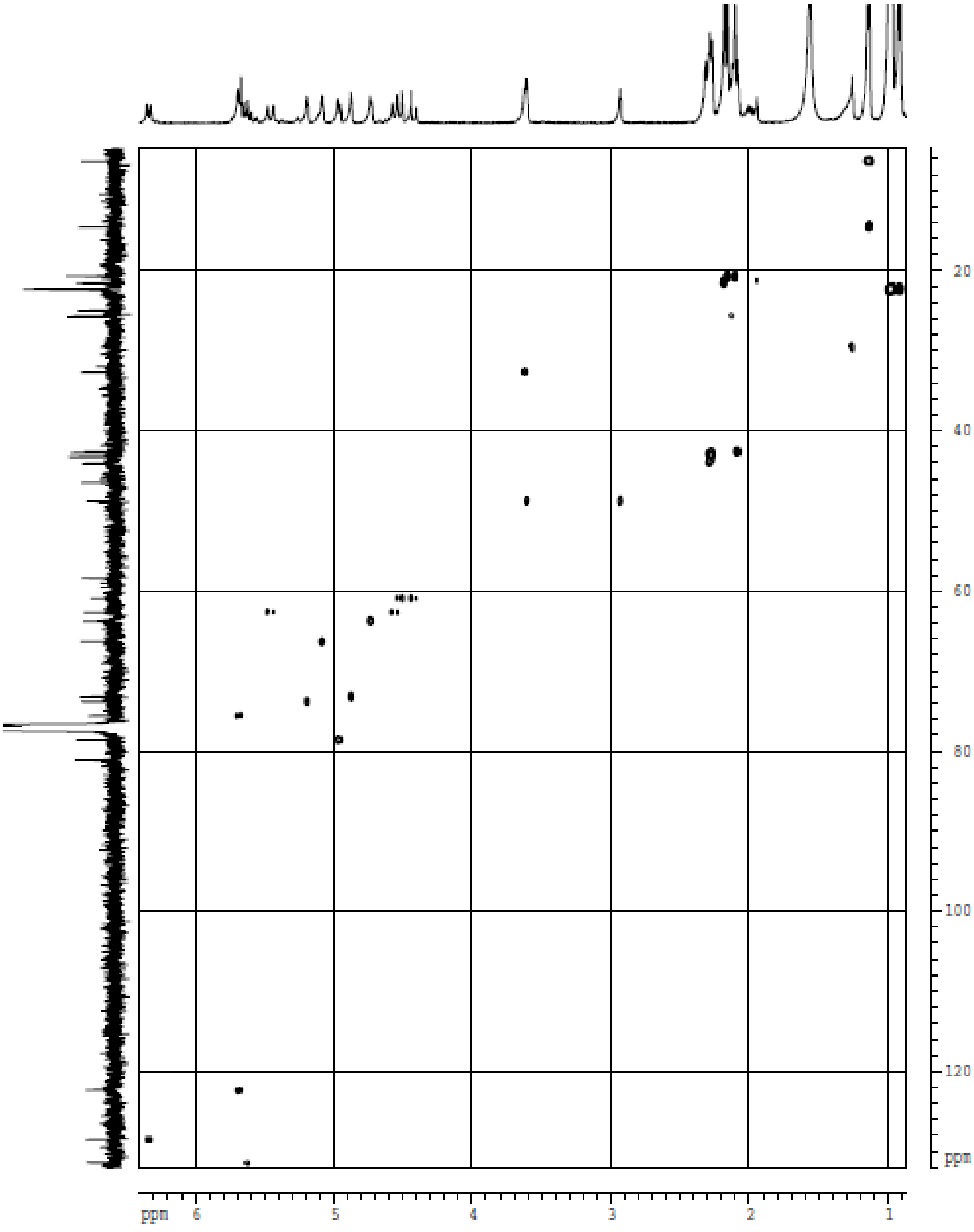

S46. ^{1}H-^{1}H COSY spectrum of the new compound **6**.

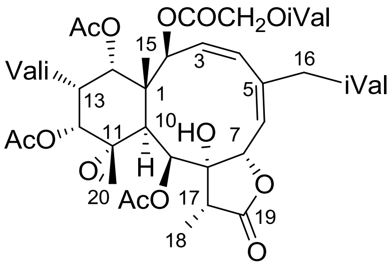

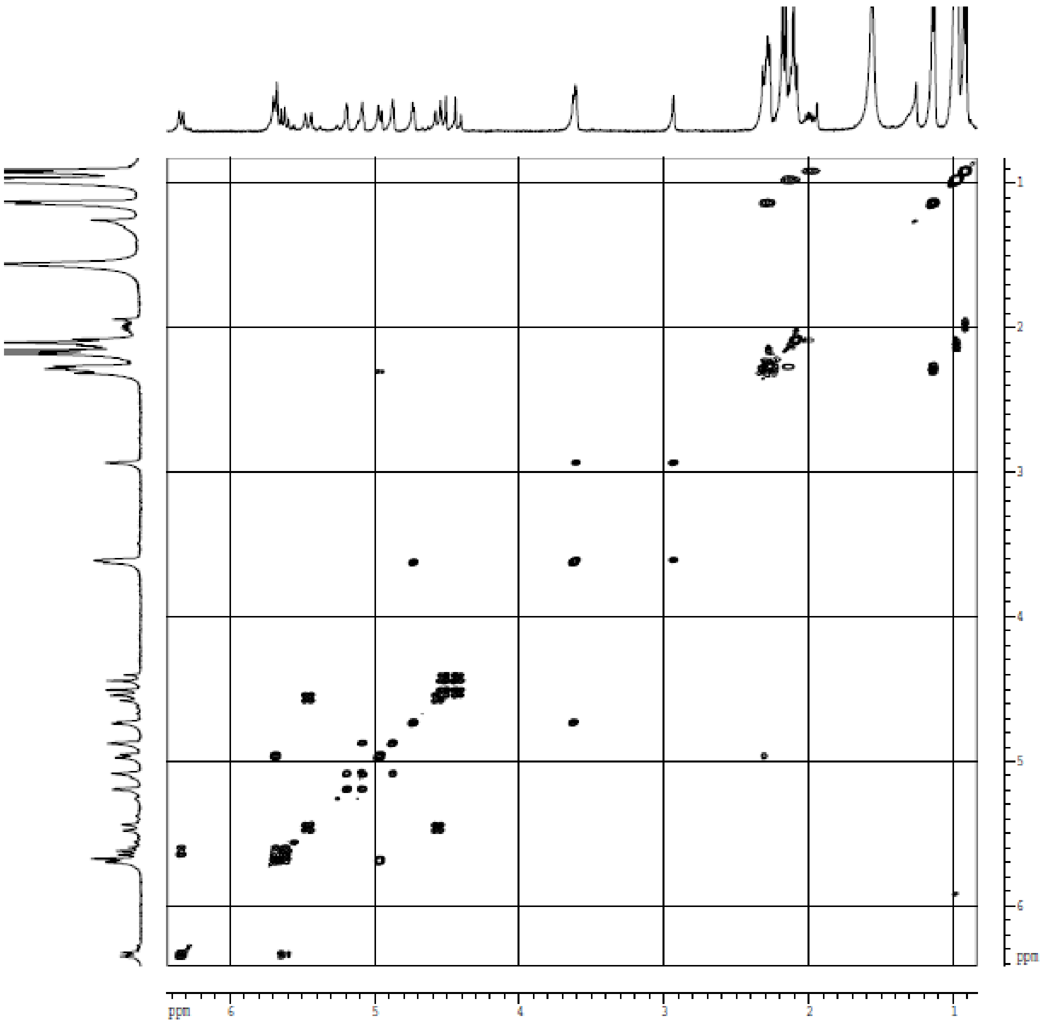

S47. HMBC spectrum of the new compound **6**.

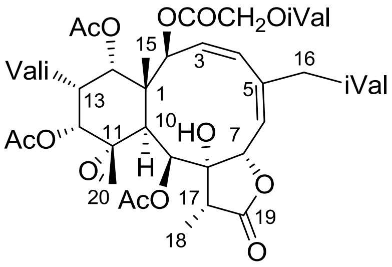

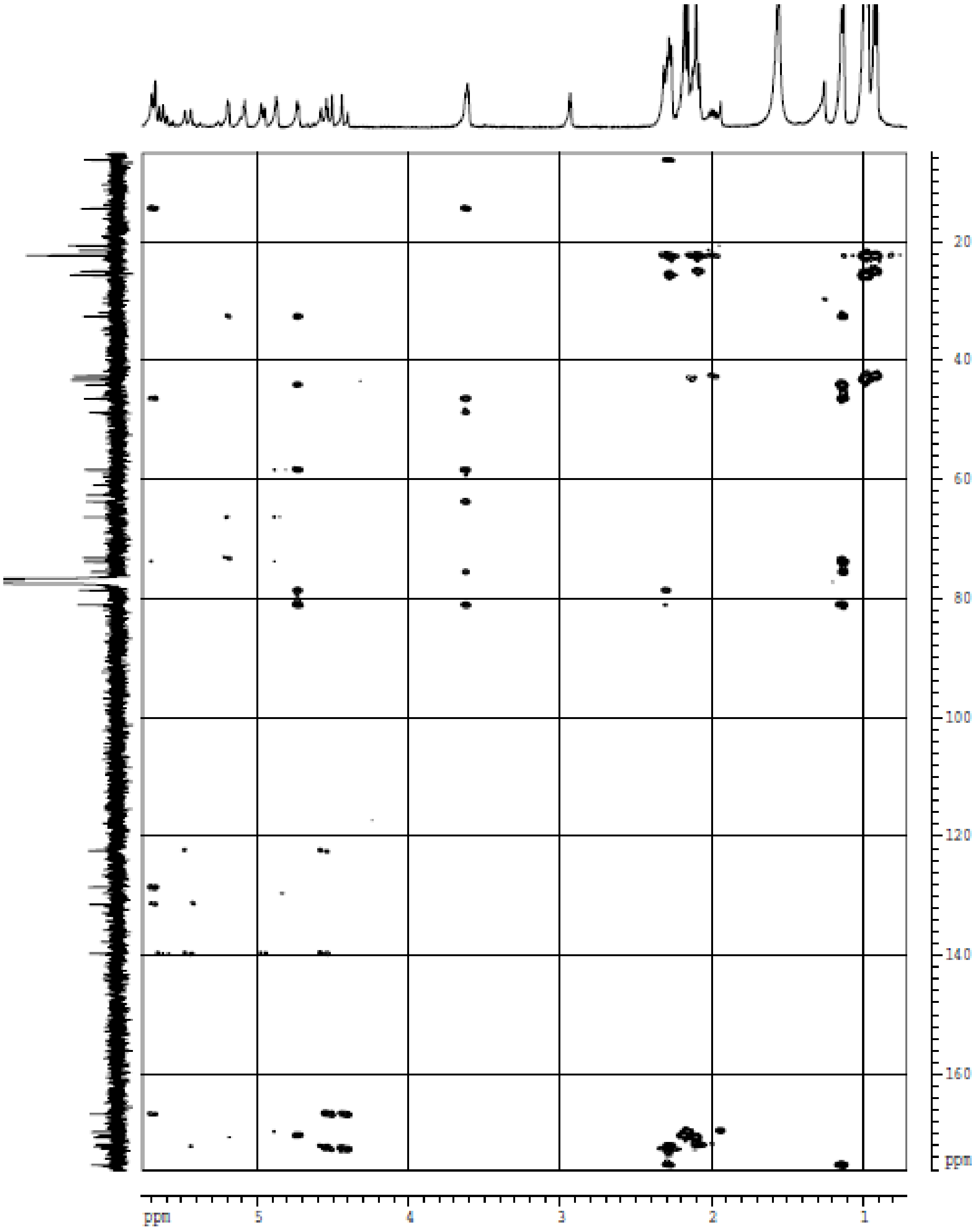

S48. NOESY spectrum of the new compound **6**.

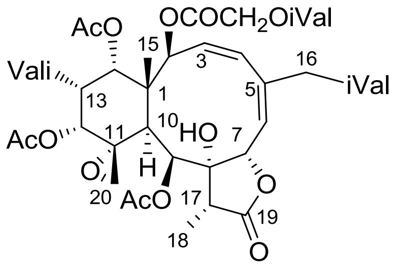

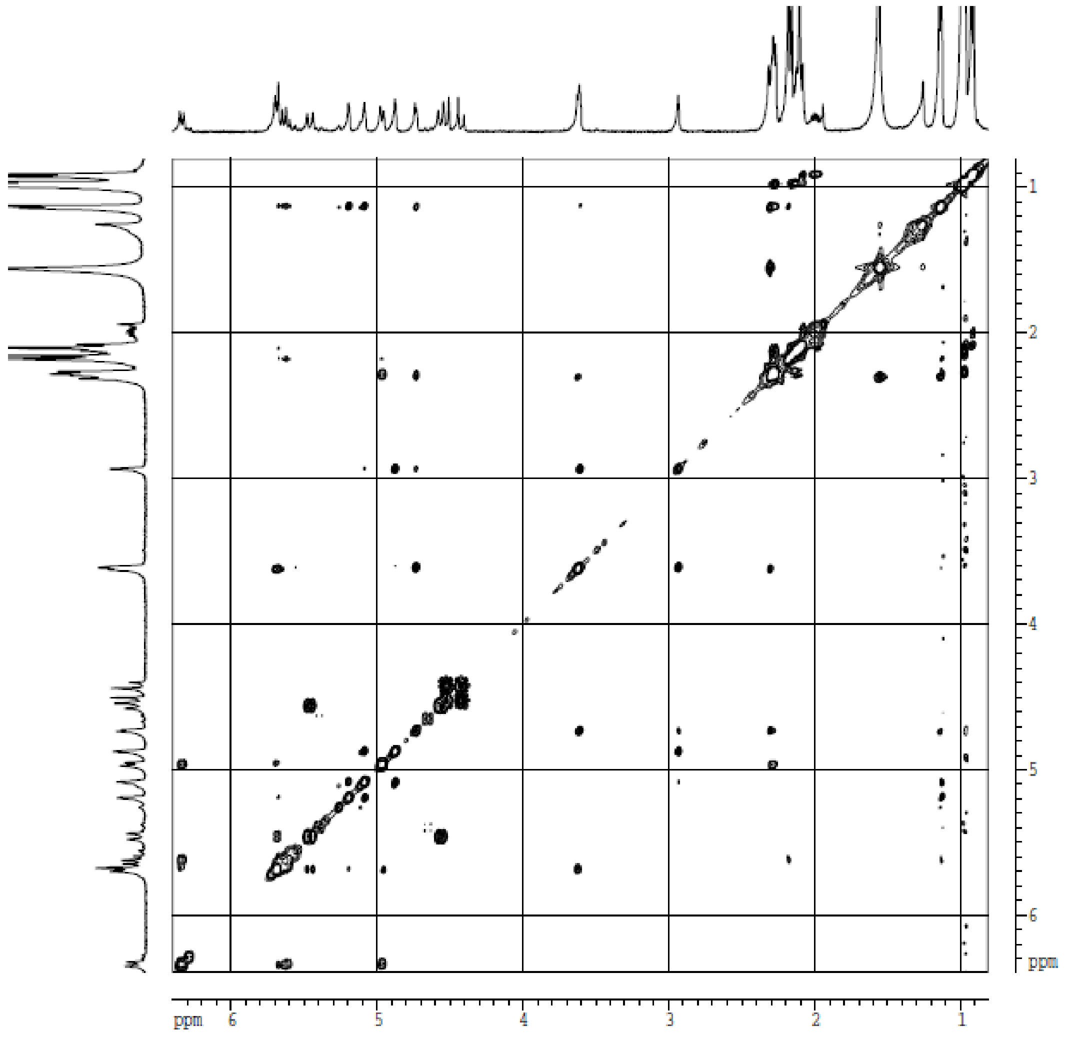

S49.

Figure S1. Four conformational isomers and populations of (1*R*,2*S*,7*S*,8*S*,9*S*,10*S*,11*R*,12*R*,14*S*,17*R*)-gemmacolide N (**1**) obtained by the B3LYP/6-31G(d) reoptimization of the seventy two MMFF conformers.

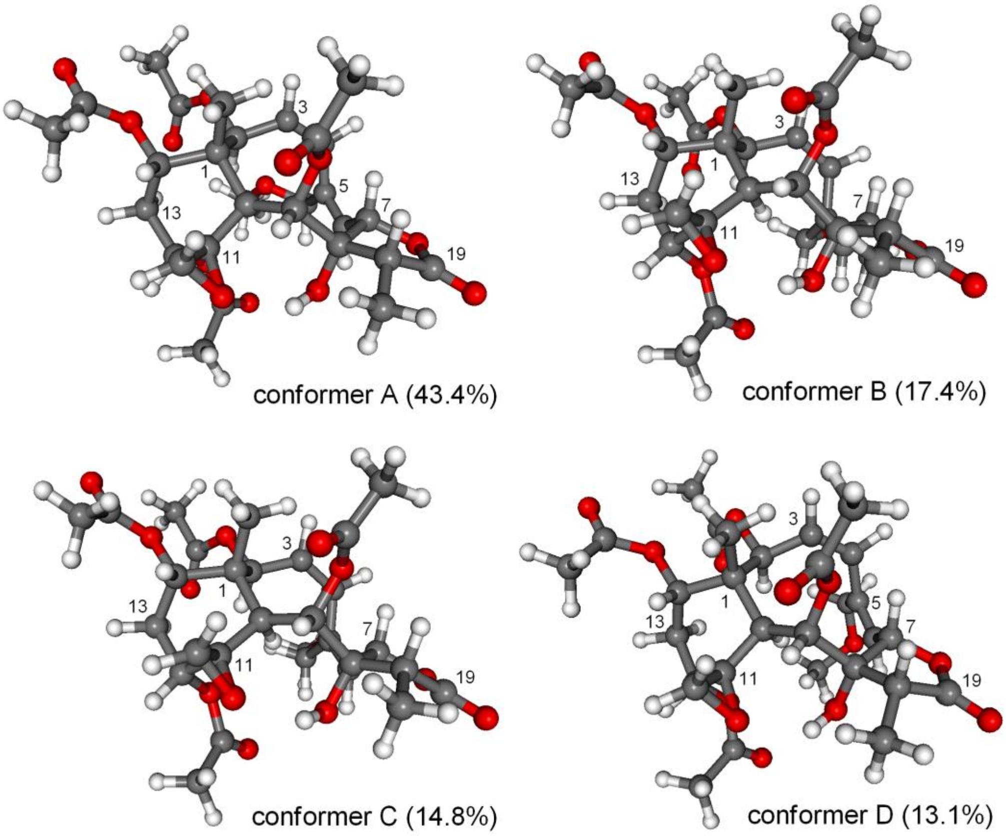

Table S1. Cartesian coordinates for the four most abundant conformers calculated at B3LYP/6-31G(d) level of theory.

Conformer A		Standard Orientation (Ångstroms)		
**I**	**Atom**	**X**	**Y**	**Z**
1	C	2.368168	0.050023	1.780589
2	C	1.002905	0.395716	2.409974
3	C	−0.017156	−0.624078	1.922037
4	C	−0.229819	−0.553399	0.392208
5	C	1.162324	−0.913203	−0.354561
6	C	2.243571	−1.105083	0.776386
7	H	0.858852	−0.165711	−3.324660
8	C	−1.603142	−1.227950	0.034963
9	C	−2.801519	−0.222421	0.023428
10	C	−2.838152	0.757824	−1.183862
11	C	−2.072050	2.032918	−1.008950
12	C	−0.948664	2.325181	−1.678785
13	C	−0.385940	1.371013	−2.677037
14	C	0.592043	0.478644	−2.488907
15	C	−4.172942	−0.926437	−0.126133
16	C	−5.040147	0.176914	−0.725339
17	O	−4.246886	1.090183	−1.344146
18	C	−0.251032	3.651086	−1.470397
19	O	1.130642	3.414769	−1.274576
20	C	1.189539	−2.216354	−1.174889
21	H	−0.376587	0.510154	0.204336
22	O	−6.240902	0.264571	−0.707024
23	C	−4.787683	−1.556049	1.121038
24	C	−0.186999	−1.859676	2.711971
25	O	−1.160041	−0.804762	2.786786
26	O	−2.783569	0.583197	1.189423
27	H	2.776043	0.931073	1.279076
28	O	−1.638779	−1.929883	−1.225419
29	O	0.649537	1.715521	1.954830
30	C	−0.163599	2.579676	2.637740
31	O	−0.478492	3.606792	2.088351
32	C	−0.569680	2.228540	4.053455
33	H	−1.232825	3.015190	4.413603
34	O	3.540657	−1.332806	0.195930
35	O	5.495005	−2.341140	0.074936
36	C	4.416920	−2.282818	0.618322
37	C	4.003473	−3.232391	1.727582
38	H	3.082162	−0.241214	2.559239
39	H	1.047709	0.374042	3.501944
40	H	1.950596	−2.014039	1.306626
41	H	−1.837511	−1.971077	0.796406

42	H	−2.520548	0.224541	−2.083306
43	H	−2.472829	2.724761	−0.274146
44	H	−0.860728	1.383883	−3.660514
45	C	1.496962	0.314740	−1.286114
46	H	−4.087491	−1.687652	−0.914357
47	H	−0.688226	4.160190	−0.600898
48	H	−0.397666	4.299172	−2.355144
49	C	1.828302	4.546164	−0.789522
50	H	0.564709	−2.145185	−2.063029
51	H	0.854044	−3.074217	−0.581588
52	H	2.213843	−2.404382	−1.504748
53	H	−5.804549	−1.888066	0.894064
54	H	−4.208642	−2.422036	1.459794
55	H	−4.853027	−0.828496	1.935329
56	H	−0.527211	−2.771557	2.222214
57	H	0.388687	−2.007669	3.625711
58	H	−2.644667	−0.002034	1.954649
59	O	2.800729	0.128105	−1.902669
60	H	1.519212	1.235871	−0.702822
61	H	1.425937	4.872490	0.180408
62	H	1.775078	5.388355	−1.498980
63	C	−1.772425	−3.285160	−1.181493
64	C	−1.906776	−3.855733	−2.570458
65	O	−1.761493	−3.933557	−0.157201
66	H	−1.079729	1.262708	4.099450
67	H	0.309661	2.179545	4.707247
68	H	4.863568	−3.860771	1.959241
69	H	3.693308	−2.699920	2.632841
70	H	3.172675	−3.873322	1.411438
71	H	−0.935630	−3.813597	−3.076840
72	H	−2.229108	−4.895496	−2.504936
73	C	3.846947	0.887305	−1.495471
74	C	5.102143	0.487535	−2.227407
75	O	3.786210	1.736299	−0.631164
76	H	4.894247	0.249434	−3.273639
77	H	5.838555	1.289487	−2.154411
78	H	5.499784	−0.412129	−1.741490
79	H	−2.614065	−3.272210	−3.166165
80	H	2.867990	4.236406	−0.668617

Table S1. *Cont.*

**Conformer B**		**Standard Orientation (Ångstroms)**		
**I**	**Atom**	**X**	**Y**	**Z**
1	C	2.538080	−0.108310	1.786058
2	C	1.174263	−0.252653	2.491057
3	C	0.334371	−1.251246	1.706145
4	C	0.033778	−0.752501	0.274793
5	C	1.422313	−0.622701	−0.547061
6	C	2.583895	−0.915553	0.480766
7	H	0.829693	0.880872	−3.174381
8	C	−1.232506	−1.499060	−0.274579
9	C	−2.567649	−0.745641	0.036059
10	C	−2.819736	0.543714	−0.796763
11	C	−2.232685	1.811563	−0.251307
12	C	−1.207445	2.458608	−0.825160
13	C	−0.600686	1.946487	−2.087475
14	C	0.505709	1.202592	−2.186673
15	C	−3.824725	−1.576161	−0.321322
16	C	−4.879990	−0.491892	−0.522585
17	O	−4.268503	0.684954	−0.819304
18	C	−0.658580	3.755635	−0.254333
19	O	−0.888105	4.855973	−1.128943
20	C	1.636310	−1.610338	−1.708437
21	H	−0.289234	0.276716	0.430194
22	O	−6.076989	−0.606439	−0.464208
23	C	−4.264755	−2.659031	0.660362
24	C	0.424722	−2.679854	2.067743
25	O	−0.711735	−1.895081	2.464660
26	O	−2.607582	−0.350722	1.397019
27	H	2.731381	0.947367	1.578431
28	O	−1.222870	−1.771703	−1.691470
29	O	0.550347	1.044603	2.468731
30	C	−0.384531	1.468664	3.380326
31	O	−0.953758	2.506754	3.155611
32	C	−0.595090	0.645425	4.633304
33	H	−1.371564	1.132092	5.223668
34	O	3.866725	−0.690575	−0.129052
35	O	5.980021	−1.179650	−0.508803
36	C	4.936541	−1.522327	−0.004986
37	C	4.770907	−2.837471	0.733617
38	H	3.343640	−0.460936	2.439891
39	H	1.288124	−0.598160	3.521669
40	H	2.501996	−1.979713	0.712843
41	H	−1.307237	−2.469318	0.215430
42	H	−2.490389	0.369598	−1.824472
43	H	−2.670925	2.179342	0.671937

44	H	−1.132718	2.182243	−3.010140
45	C	1.475336	0.858656	−1.079174
46	H	−3.673023	−2.023848	−1.313668
47	H	0.430060	3.696664	−0.149476
48	H	−1.087451	3.938088	0.741257
49	C	−2.229593	5.307262	−1.127778
50	H	0.969352	−1.400998	−2.542737
51	H	1.477951	−2.646759	−1.390606
52	H	2.662193	−1.512990	−2.072391
53	H	−5.233003	−3.058488	0.346237
54	H	−3.545692	−3.484638	0.696902
55	H	−4.390144	−2.249034	1.666871
56	H	0.220801	−3.440421	1.314428
57	H	1.063874	−2.997995	2.891640
58	H	−2.357663	−1.118559	1.940228
59	O	2.767054	1.126890	−1.681350
60	H	1.349002	1.536054	−0.233704
61	H	−2.544527	5.630087	−0.122766
62	H	−2.932369	4.536614	−1.477377
63	C	−1.134113	−3.076981	−2.074055
64	C	−1.256638	−3.206659	−3.570964
65	O	−0.958585	−3.996763	−1.304204
66	H	−0.897194	−0.378189	4.396005
67	H	0.326220	0.599549	5.226499
68	H	5.746313	−3.323251	0.761825
69	H	4.413413	−2.691657	1.758545
70	H	4.061945	−3.495707	0.219019
71	H	−2.075064	−2.589475	−3.951346
72	H	−0.331918	−2.856803	−4.044253
73	C	3.591665	2.015607	−1.070154
74	C	4.906443	2.102195	−1.797988
75	O	3.303664	2.621130	−0.058548
76	H	5.486250	1.201681	−1.560982
77	H	4.753395	2.130890	−2.880257
78	H	5.451349	2.986499	−1.464758
79	H	−1.414146	−4.253876	−3.831469
80	H	−2.273847	6.163325	−1.806486

Table S1. *Cont.*

**Conformer C**		**Standard Orientation (Ångstroms)**		
**I**	**Atom**	**X**	**Y**	**Z**
1	C	2.427858	−0.381657	1.808583
2	C	1.049648	−0.316934	2.497562
3	C	0.094868	−1.230883	1.741408
4	C	−0.131752	−0.753370	0.289534
5	C	1.269248	−0.817372	−0.519035
6	C	2.376817	−1.226511	0.527939
7	H	0.903643	0.682204	−3.191242
8	C	−1.474089	−1.362578	−0.248444
9	C	−2.710562	−0.440993	0.015019
10	C	−2.794760	0.833757	−0.872609
11	C	−2.081588	2.047448	−0.356865
12	C	−0.977758	2.552688	−0.924823
13	C	−0.404929	1.938665	−2.157647
14	C	0.604408	1.063978	−2.217131
15	C	−4.053022	−1.131796	−0.328083
16	C	−4.968177	0.057721	−0.604023
17	O	−4.216792	1.137875	−0.941592
18	C	−0.280367	3.766696	−0.357938
19	O	−0.241775	4.763990	−1.369957
20	C	1.368994	−1.851868	−1.654661
21	H	−0.334206	0.311269	0.405214
22	O	−6.171346	0.086501	−0.568313
23	C	−4.632085	−2.104859	0.695440
24	C	0.002811	−2.644071	2.158405
25	O	−1.029143	−1.707937	2.510258
26	O	−2.714339	0.011345	1.358695
27	H	2.765595	0.630951	1.573361
28	O	−1.487180	−1.685602	−1.654532
29	O	0.601631	1.048205	2.411274
30	C	−0.264593	1.638540	3.297402
31	O	−0.674919	2.738870	3.027028
32	C	−0.600825	0.903751	4.577496
33	H	−1.303783	1.519561	5.138673
34	O	3.682404	−1.185709	−0.075038
35	O	5.725497	−1.942121	−0.396904
36	C	4.639241	−2.138208	0.094869
37	C	4.298773	−3.399955	0.865690
38	H	3.170438	−0.820732	2.484388
39	H	1.108058	−0.630304	3.543090
40	H	2.157297	−2.264111	0.789320
41	H	−1.672462	−2.297561	0.274718
42	H	−2.460464	0.581755	−1.882271
43	H	−2.500201	2.500222	0.536921

44	H	−0.886769	2.217758	−3.095186
45	C	1.509692	0.632705	−1.085570
46	H	−3.941409	−1.643433	−1.294416
47	H	0.748606	3.508362	−0.052954
48	H	−0.804627	4.123636	0.540424
49	C	0.492647	5.901985	−0.969263
50	H	0.734939	−1.585237	−2.498151
51	H	1.088086	−2.854187	−1.312792
52	H	2.400814	−1.885417	−2.013263
53	H	−5.636102	−2.403438	0.381524
54	H	−4.015896	−3.006121	0.785886
55	H	−4.722195	−1.632846	1.678222
56	H	−0.288133	−3.402609	1.432163
57	H	0.589874	−3.006888	3.002394
58	H	−2.566920	−0.758759	1.935109
59	O	2.833529	0.724051	−1.672205
60	H	1.456568	1.341773	−0.258907
61	H	1.538312	5.648664	−0.731494
62	H	0.045496	6.386240	−0.086184
63	C	−1.550840	−3.004707	−1.991337
64	C	−1.678265	−3.170531	−3.484352
65	O	−1.491705	−3.911208	−1.188678
66	H	−1.045330	−0.074615	4.375689
67	H	0.299390	0.749419	5.184684
68	H	5.203424	−4.005517	0.921575
69	H	3.951756	−3.179790	1.880850
70	H	3.516550	−3.976044	0.358876
71	H	−2.438773	−2.496173	−3.887786
72	H	−0.725840	−2.916302	−3.963432
73	C	3.751578	1.526377	−1.077265
74	C	5.077945	1.422873	−1.782125
75	O	3.526940	2.200856	−0.093139
76	H	4.946155	1.431715	−2.867550
77	H	5.724399	2.243559	−1.468069
78	H	5.537007	0.466076	−1.504448
79	H	−1.927430	−4.207195	−3.713297
80	H	0.476208	6.605825	−1.805865

Table S1. *Cont.*

**Conformer D**		**Standard Orientation (Ångstroms)**		
**I**	**Atom**	**X**	**Y**	**Z**
1	C	2.393359	0.531767	1.561629
2	C	1.109232	0.533593	2.412252
3	C	0.179719	−0.576873	1.932421
4	C	−0.052377	−0.570311	0.403861
5	C	1.332389	−0.906521	−0.352306
6	C	2.471452	−0.753218	0.731397
7	H	0.850621	−0.817559	−3.375440
8	C	−1.392643	−1.331041	0.100057
9	C	−2.637827	−0.386122	0.024781
10	C	−2.815229	0.381463	−1.316270
11	C	−2.117174	1.700537	−1.453234
12	C	−1.044008	1.881140	−2.233126
13	C	−0.478627	0.757402	−3.031512
14	C	0.551726	−0.024324	−2.693321
15	C	−3.973035	−1.169143	0.092566
16	C	−4.943294	−0.226357	−0.614243
17	O	−4.248449	0.619578	−1.416148
18	C	−0.393769	3.244224	−2.439803
19	O	−0.968858	4.289120	−1.694632
20	C	1.488243	−2.334820	−0.907447
21	H	−0.257613	0.478874	0.177949
22	O	−6.144269	−0.205736	−0.528092
23	C	−4.472450	−1.605266	1.467957
24	C	0.071319	−1.800556	2.752874
25	O	−0.951006	−0.788385	2.800408
26	O	−2.575575	0.590156	1.048894
27	H	2.406374	1.406780	0.906455
28	O	−1.395195	−2.144672	−1.092812
29	O	0.478415	1.811776	2.238924
30	C	−0.201646	2.474565	3.229474
31	O	−0.746935	3.507882	2.936179
32	C	−0.197068	1.899996	4.631878
33	H	−0.797668	2.559833	5.258192
34	O	3.768281	−0.833660	0.117716
35	O	5.891359	−1.418979	0.083852
36	C	4.827800	−1.500789	0.651655
37	C	4.626628	−2.327600	1.907718
38	H	3.279228	0.605631	2.201023
39	H	1.336983	0.370479	3.468461
40	H	2.359421	−1.614683	1.395142
41	H	−1.590198	−2.015873	0.922086
42	H	−2.540548	−0.290373	−2.133492
43	H	−2.542403	2.535216	−0.906046

44	H	−0.977713	0.546977	−3.979836
45	C	1.470355	0.154728	−1.508339
46	H	−3.890364	−2.051216	−0.558894
47	H	−0.505317	3.513810	−3.500460
48	H	0.690399	3.170066	−2.250507
49	C	−0.406812	4.457477	−0.396643
50	H	0.870895	−2.498147	−1.788365
51	H	1.223096	−3.089657	−0.159125
52	H	2.529621	−2.491069	−1.201907
53	H	−5.470280	−2.040751	1.367211
54	H	−3.811670	−2.353723	1.918291
55	H	−4.556147	−0.749488	2.144457
56	H	−0.228780	−2.739669	2.289946
57	H	0.653965	−1.896043	3.668991
58	H	−2.456330	0.126935	1.895998
59	O	2.788449	0.090112	−2.109534
60	H	1.346609	1.150786	−1.082664
61	H	0.674458	4.651943	−0.459498
62	H	−0.576771	3.593354	0.254376
63	C	−1.461900	−3.495797	−0.928778
64	C	−1.593968	−4.190004	−2.260864
65	O	−1.403391	−4.052104	0.146706
66	H	−0.618145	0.890924	4.648178
67	H	0.819897	1.856510	5.039454
68	H	5.597132	−2.734104	2.192450
69	H	4.231435	−1.729479	2.735615
70	H	3.933294	−3.157507	1.730149
71	H	−2.364036	−3.711904	−2.872690
72	H	−0.647409	−4.118389	−2.808708
73	C	3.616499	1.153858	−1.943480
74	C	4.960181	0.879975	−2.563058
75	O	3.305868	2.165263	−1.347839
76	H	5.503222	0.189205	−1.906398
77	H	4.849789	0.400572	−3.539617
78	H	5.518054	1.812908	−2.655016
79	H	−1.833907	−5.241346	−2.098922
80	H	−0.901198	5.325445	0.045597

S50.

Figure S2. Experimental solution ECD spectrum of gemmacolide N (**1**) compared with the BH&HLYP/6-311G(d,p) ECD spectrum calculated for the lowest-energy conformational isomer (conformer A, 43.4%) of the (1*R*,2*S*,7*S*,8*S*,9*S*,10*S*,11*R*,12*R*,14*S*,17*R*)-enantiomer of **1**. Bars represent the rotational strength with the BH&HLYP/6-311G(d,p) method.

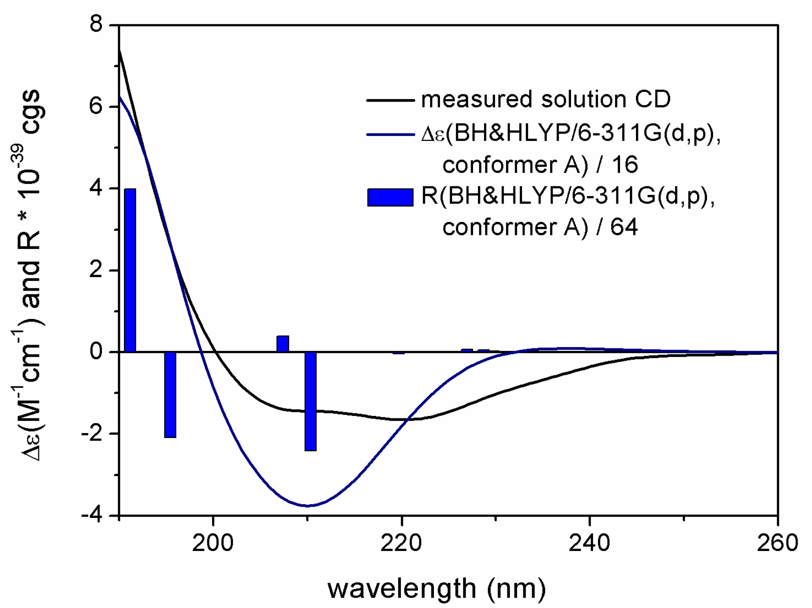

Figure S3. Experimental solution ECD spectrum of gemmacolide N (**1**) compared with the BH&HLYP/6-311G(d,p) ECD spectrum calculated for conformer B (17.4%) of the (1*R*,2*S*,7*S*,8*S*,9*S*,10*S*,11*R*,12*R*,14*S*,17*R*)-enantiomer of **1**. Bars represent the rotational strength with the BH&HLYP/6-311G(d,p) method.

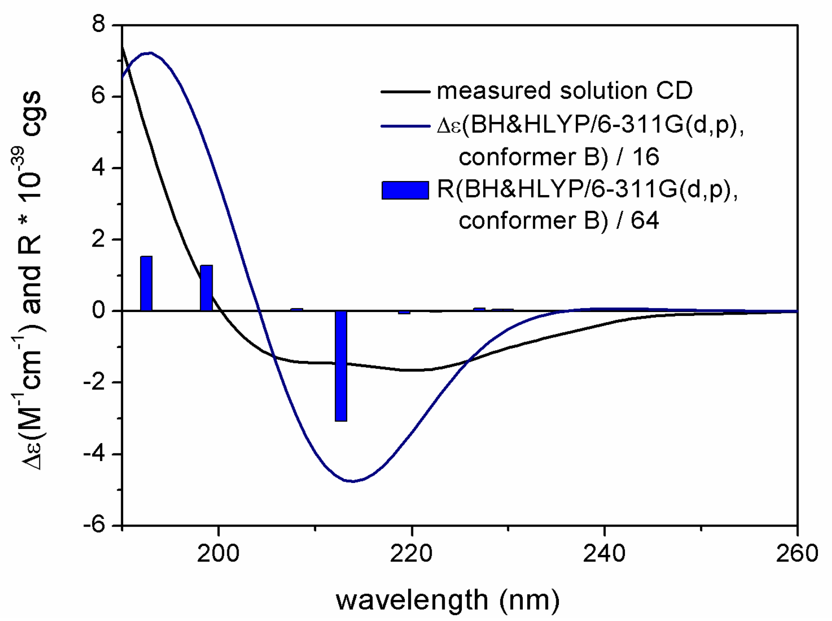

Figure S4. Experimental solution ECD spectrum of gemmacolide N (**1**) compared with the BH&HLYP/6-311G(d,p) ECD spectrum calculated for conformer C (14.8%) of the (1*R*,2*S*,7*S*,8*S*,9*S*,10*S*,11*R*,12*R*,14*S*,17*R*)-enantiomer of **1**. Bars represent the rotational strength with the BH&HLYP/6-311G(d,p) method.

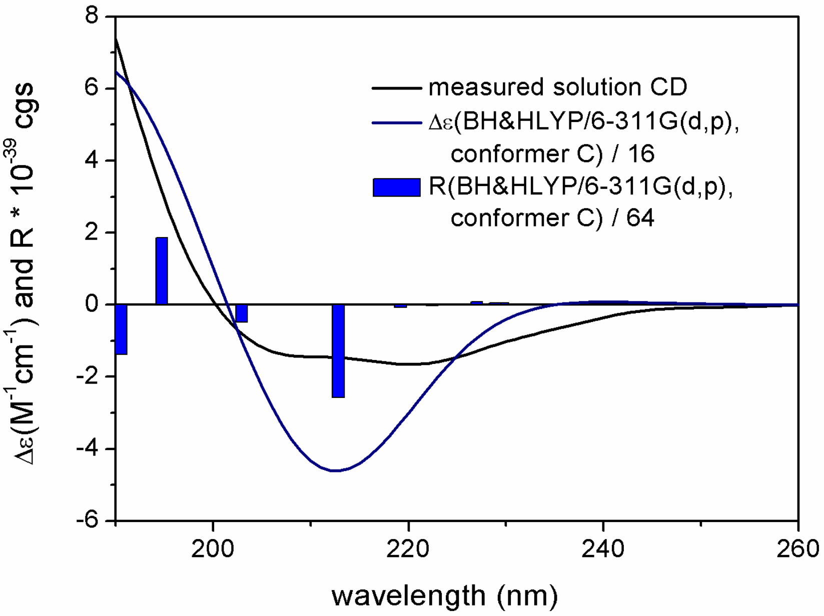

Figure S5. Experimental solution ECD spectrum of gemmacolide N (**1**) compared with the BH&HLYP/6-311G(d,p) ECD spectrum calculated for conformer D (13.1%) of the (1*R*,2*S*,7*S*,8*S*,9*S*,10*S*,11*R*,12*R*,14*S*,17*R*)-enantiomer of **1**. Bars represent the rotational strength with the BH&HLYP/6-311G(d,p) method.

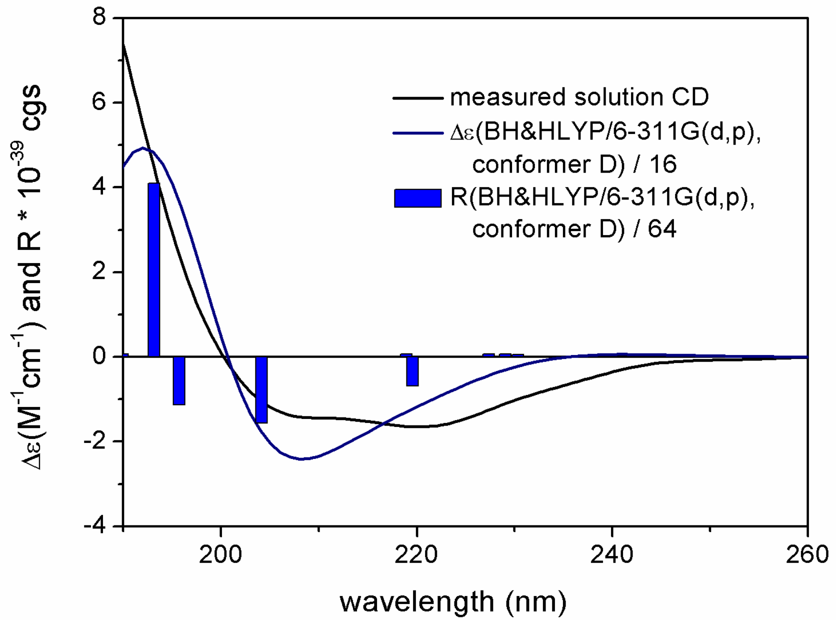

Supplement: Supplementary file 1 [file marinedrugs-09-01403-001.pdf]
